# Genetic basis of the highly efficient yeast *Kluyveromyces marxianus*: complete genome sequence and transcriptome analyses

**DOI:** 10.1186/s13068-015-0227-x

**Published:** 2015-03-18

**Authors:** Noppon Lertwattanasakul, Tomoyuki Kosaka, Akira Hosoyama, Yutaka Suzuki, Nadchanok Rodrussamee, Minenosuke Matsutani, Masayuki Murata, Naoko Fujimoto, Suprayogi, Keiko Tsuchikane, Savitree Limtong, Nobuyuki Fujita, Mamoru Yamada

**Affiliations:** Applied Molecular Bioscience, Graduate School of Medicine, Yamaguchi University, Ube, 755-8505 Japan; Department of Biological Chemistry, Faculty of Agriculture, Yamaguchi University, Yamaguchi, 753-8515 Japan; National Institute of Technology and Evaluation, Shibuya-ku, Tokyo 151-0066 Japan; Department of Medical Genome Sciences, The University of Tokyo, Chiba, 277-8562 Japan; Department of Microbiology, Faculty of Science, Kasetsart University, Bangkok, 10900 Thailand; Department of Biology, Faculty of Science, Chiang Mai University, Chiang Mai, 50200 Thailand

**Keywords:** *Kluyveromyces marxianus*, Thermotolerant yeast, Complete genome sequence, Transcriptome analysis, Xylose fermentation

## Abstract

**Background:**

High-temperature fermentation technology with thermotolerant microbes has been expected to reduce the cost of bioconversion of cellulosic biomass to fuels or chemicals. Thermotolerant *Kluyveromyces marxianus* possesses intrinsic abilities to ferment and assimilate a wide variety of substrates including xylose and to efficiently produce proteins. These capabilities have been found to exceed those of the traditional ethanol producer *Saccharomyces cerevisiae* or lignocellulose-bioconvertible ethanologenic *Scheffersomyces stipitis*.

**Results:**

The complete genome sequence of *K. marxianus* DMKU 3-1042 as one of the most thermotolerant strains in the same species has been determined. A comparison of its genomic information with those of other yeasts and transcriptome analysis revealed that the yeast bears beneficial properties of temperature resistance, wide-range bioconversion ability, and production of recombinant proteins. The transcriptome analysis clarified distinctive metabolic pathways under three different growth conditions, static culture, high temperature, and xylose medium, in comparison to the control condition of glucose medium under a shaking condition at 30°C. Interestingly, the yeast appears to overcome the issue of reactive oxygen species, which tend to accumulate under all three conditions.

**Conclusions:**

This study reveals many gene resources for the ability to assimilate various sugars in addition to species-specific genes in *K. marxianus*, and the molecular basis of its attractive traits for industrial applications including high-temperature fermentation. Especially, the thermotolerance trait may be achieved by an integrated mechanism consisting of various strategies. Gene resources and transcriptome data of the yeast are particularly useful for fundamental and applied researches for innovative applications.

**Electronic supplementary material:**

The online version of this article (doi:10.1186/s13068-015-0227-x) contains supplementary material, which is available to authorized users.

## Background

Along with rising concern about global warming and the rapid increase in fuel consumption, there is worldwide interest in the production of bioethanol from renewable resources [1]. For economically sustainable production of bioethanol, it is necessary to increase the types of biomass such as lignocellulosic materials that can be used without competing with food supplies. Accordingly, microbes that can efficiently convert various sugars in these kinds of biomass to ethanol must be developed.

A high-temperature fermentation (HTF) technology is expected to help reduce cooling cost, efficiently achieve simultaneous saccharification and fermentation, reduce the risk of contamination, and offer stable fermentation even in tropical countries [2,3]. Thermotolerant yeast *Kluyveromyces marxianus*, which is able to ferment various sugars, may be a suitable microbe for HTF with lignocellulosic hydrolysates [4,5].

*K. marxianus* is a haploid, homothallic, thermotolerant, hemiascomycetous yeast [6,7] and a close relative of *Kluyveromyces lactis*, a model Crabtree-negative yeast [8-11]. Both yeasts share the assimilating capability of lactose, which is absent from *Saccharomyces cerevisiae. K. marxianus* has a number of advantages over *K. lactis* or *S. cerevisiae*, including the intrinsic fermentation capability of various sugars at high temperatures [4,12,13], weak glucose repression that is preferable for mixed sugars such as hemicellulose hydrolysate, and fermentability of inulin [13,14]. However, its fermentation activity from xylose is extremely low compared to that of glucose. Recently, we developed a procedure that improves this disadvantageous trait (unpublished data) and increases the fermentation activity to slightly less than that of *Scheffersomyces stipitis* at around 30°C and a much higher activity at higher temperatures. Many biotechnological applications of *K. marxianus* have so far been achieved: production of various enzymes including heterologous proteins, aroma compounds or bioingredients, reduction of lactose content in food products, production of ethanol or single-cell protein, and bioremediation [13]. In addition, novel methods and genetic tools for genetic engineering have been developed on the basis of its high nonhomologous end-joining activity [15,16]. *K. marxianus* is thus a highly competent yeast for future developments. In order to facilitate such developments, its genomic information is essential. Draft genome sequences of three *K. marxianus* strains have been published [7,17,18], but no detailed analysis is available.

This study provides core information on *K. marxianus* DMKU 3-1042, which is one of the most thermotolerant strains in the same species isolated (3, unpublished data), including its ability to assimilate various sugars and the molecular basis of its thermotolerance and efficient protein productivity, in addition to the complete genome sequence.

## Results

### Genomic information and comparative genomics

The genome sequence of *K. marxianus* DMKU 3-1042 was precisely determined (less than one estimated error per chromosome) by nucleotide sequencing with three different sizes of shotgun libraries. Telomeric regions were further analyzed by transposon-insertion sequencing of corresponding fosmid clones. This strategy allowed us to determine the complete genome sequence of 11.0 Mb including all centromeric regions and boundary regions containing up to one to several sequence repeats (GGTGTACGGATTTGATTAGTTATGT) of telomeres. Optical mapping confirmed the genome organization except for three inverted regions, which were fixed in the final complete sequences (Additional file 1: Figure S1). There are eight chromosomes ranging in size from 0.9 to 1.7 Mb and a mitochondrial genome of 46 kb. The annotation process predicted 4,952 genes (Table 1), of which 98.0% were predicted to consist of a single exon (Additional file 2). The average gene density is 68.0% (Table 2). The average gene and protein lengths are 1.5 kb and 501 amino acids, respectively (Table 1).

**Table 1:** **General information of nuclear and mitochondrial genomes of** ***K. marxianus*** **DMKU 3-1042**

	**Length**	**CDS**	**Intron-containing CDS**	**tRNA**	**rDNA**	**na_length average**	**na_length maximum**	**aa_length average**	**aa_length maximum**
Total	10,966,467	4,952	172	202	8^b^				
Average						1,505		501	
Chromosome									
1	1,745,387	803	25	31	0	1,473	12,174	491	4,058
2	1,711,476	808	29	28	0	1,440	6,168	480	2,056
3	1,588,169	706	21	24	0	1,553	8,004	517	2,668
4	1,421,472	624	26	25	0	1,593	14,742	531	4,914
5	1,353,011^a^	611	20	30	6^b^	1,529	9,018	509	3,006
6	1,197,921	537	18	17	0	1,500	8,709	500	2,903
7	963,005	438	19	10	0	1,454	8,310	484	2,770
8	939,718	414	12	15	0	1,523	9,381	507	3,127
Mitochondrion	46,308	11	2	22	2	978	1,491	326	497

^a^The length does not include that of most of rDNA. ^b^Six rDNA copies in the genome sequence in database, but 140 rDNA copies by optical mapping. CDS, coding DNA sequence.

**Table 2:** **General characteristics of 11 hemiascomycetous yeast genomes**

**Species**	**Genome size (Mb)**	**Average G + C content (%)**	**Total CDS**	**Total tRNA genes**	**Average gene density (%)**	**Average G + C in CDS (%)**	**Source**
*Kluyveromyces marxianus*	10.97	40.12	4,952	202	68.00	41.69	This study
*Kluyveromyces lactis*	10.60	38.70	5,329	162	71.60	40.10	[20]
*Saccharomyces cerevisiae*	12.10	38.30	5,807	274	70.30	39.60	[20]
*Candida glabrata*	12.30	38.80	5,283	207	65.00	41.00	[20]
*Yarrowia lipolytica*	20.50	49.00	6,703	510	46.30	52.90	[20]
*Scheffersomyces stipitis*	15.40	41.10	5,841	-	55.90	42.70	[21]
*Ashbya gossypii*	9.12	51.70	4,776	220	77.10	52.80	[19]
*Ogataea parapolymorpha*	8.87	47.83	5,325	80	84.58	49.13	This study^a^
*Debaryomyces hansenii*	12.18	36.34	6,290	225	74.31	37.45	[20]
*Clavispora lusitaniae*	12.11	44.50	5,936	217	68.07	46.80	[22]
*Schizosaccharomyces pombe*	12.59	36.04	5,133	195	57.17	39.63	[23]

^a^Values were summarized by us using data from Joint Genome Institute (JGI, http://gold.jgi-psf.org). G + C, guanine + cytosine; CDS, coding DNA sequence.

**Figure 1:**
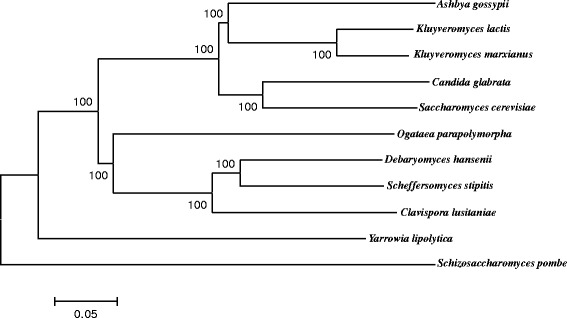
**Phylogenetic tree of 11 hemiascomycetous yeast genomes based on 1,361 concatenated amino acid sequences.** *K. marxianus* shares 1,552 genes with *K. lactis* NRRL Y-1140 (CR382121-CR382126), *Ashbya gossypii* (*Eremothecium gossypii*) ATCC 10895 (AE016814-AE016821), *Candida glabrata* CBS 138 (CR380947-CR380959), *S. cerevisiae* S288c (BK006934-BK006949), *Ogataea parapolymorpha* DL-1 (AEOI00000000), *Debaryomyces hansenii* CBS 767 (CR382133-CR382139), *S. stipitis* CBS 6054 (AAVQ00000000), *Clavispora lusitaniae* ATCC 42720 (AAFT00000000), *Yarrowia lipolytica* CLIB122 (CR382127-CR382132) and *Schizosaccharomyces pombe* 972 h- (CU329670-CU329672) as hemiascomycetous yeasts*,* of which complete or draft genome sequences are available [19-23]. A whole genome-wide phylogenetic tree with amino acid sequences deduced from the conserved 1,361 genes was constructed using the neighbor-joining algorithm of MEGA5.05 as previously reported [31]. The numbers at each branch indicate bootstrap values.

Eukaryotic orthologous groups (KOG) database analysis led to the assignment of protein functions of about 72.4% of predicted genes (Additional file 1: Table S1) and protein domains were predicted in 3,584 gene models*.* UniProt and KAAS assignments led to the assignment of homologous genes of about 86.4% of predicted genes and KEGG Orthology number of 50.5%, respectively. The yeast shares 1,552 genes with *K. lactis*, *Ashbya gossypii*, *Candida glabrata*, *S. cerevisiae*, *Ogataea parapolymorpha*, *Debaryomyces hansenii*, *S. stipitis*, *Clavispora lusitaniae*, *Yarrowia lipolytica*, and *Schizosaccharomyces pombe* as hemiascomycetous yeasts [19-23]. The phylogenetic tree exhibits the closest location of *K. marxianus* to *K. lactis* and closer to *A. gossypii*, *C. glabrata*, and *S. cerevisiae* in the 11 yeasts (Figure 1)*.* Consistent with this, *K. marxianus* shares 4,676; 3,826; 3,672; and 3,853 genes with *K. lactis*, *A. gossypii*, *C. glabrata*, and *S. cerevisiae*, respectively. On the other hand, there are 193 genes specific for *K. marxianus* (Additional file 1: Table S2), which may be responsible for its species-specific characteristics, of which two thirds of the genes could not be assigned by the KOG database (Additional file 1: Table S3). There are 422 genes shared only between *K. marxianus* and *K. lactis* (Additional file 1: Table S4), which may be related to their genus-specific characteristics, such as production of β-galactosidase [24], assimilation of a wide variety of inexpensive substrates [25], efficient productivity of heterologous proteins [26-28], and synthesis of a killer toxin against certain ascomycetous yeasts [29,30].

The two most attractive traits of *K. marxianus* for fermentation applications are thermotolerance and pentose assimilation capability, which are also found in *O. parapolymorpha* and *S. stipitis*, respectively. The number of genes that are shared between the two thermotolerant yeasts but absent from *K. lactis* is 30, including genes for three siderophore-iron transporters and three vacuolar proteins (Additional file 1: Table S5). Notably, there are 27 putative sugar transporters in the *K. marxianus* genome (Additional file 1: Table S6). Like *S. stipitis*, the initial xylose catabolism after its uptake in *K. marxianus* is accomplished by three genes, *XYL1*, *XYL2*, and *XKS1*, which are involved in the conversion of xylose to xylulose-5-phosphate as an intermediate in the pentose phosphate pathway (PPP). Genes for utilization of various other sugars and alcohol dehydrogenases are listed (Table 3).

**Table 3:** **Genes for utilization of sugars at their initial catabolism and genes for alcohol dehydrogenases in** ***K. marxianus***

	**Product**	**UniProt gene**	**Sugar**
KMLA_60412	Hexokinase	HXK2 (RAG5)	Glucose, fructose, mannose
KLMA_10763	Glucose-6-phosphate dehydrogenase	RAG2	
KLMA_50384	Mannose 6-phosphate isomerase	PMI40	Mannose
KLMA_20333	Galactokinase	GAL1	Galactose
KLMA_20331	Galactose-1-phosphate uridylyltransferase	GAL7	
KLMA_30099	Phosphoglucomutase	GAL5 (PGM2)	
KLMA_10683	Xylose reductase	XYL1	Xylose
KLMA_70044	Xylitol dehydrogenase	XYL2	
KLMA_80066	Xylulokinase	XKS1	
KLMA_30577	Arabinose dehydrogenase [NADP^+^ dependent]	ARA1	Arabinose
KLMA_40310	Arabinose dehydrogenase [NAD dependent]	ARA2	
KLMA_80176	Xylose/arabinose reductase	YJR096W	
KLMA_10558	D-Arabitol-2-dehydrogenase	ARD2	
KLMA_10157	Probable ribokinase	RBK1	D-Ribose
KLMA_10176	Ribose-phosphate pyrophosphokinase 5	PRS5	
KLMA_10783	Sorbose reductase	SOU1	Mannitol, glucitol, L-sorbose
KLMA_10649	D-Lactate dehydrogenase [cytochrome], mitochondrial	DLD1	Lactate
KLMA_40583	D-Lactate dehydrogenase [cytochrome] 1, mitochondrial	DLD1	
KLMA_50301	D-Lactate dehydrogenase [cytochrome], mitochondrial	DLD1	
KLMA_60482	D-Lactate dehydrogenase [cytochrome] 2, mitochondrial	DLD2	
KLMA_10179	Glycerol-3-phosphate dehydrogenase [NAD(+)] 1	GPD1	Glycerol
KLMA_30722	Glycerol-3-phosphate dehydrogenase, mitochondrial	GUT2	
KLMA_60361	Glycerol uptake protein 1	GUP1	
KLMA_80411	Glycerol uptake/efflux facilitator protein	FPS1	
KLMA_80412	Glycerol kinase	GUT1	
KLMA_10427	Galactose/lactose metabolism regulatory protein GAL80	GAL80	Lactose
KLMA_20830	Lactose permease	LAC12	
KLMA_30010	Lactose permease	LAC12	
KLMA_30728	Lactose permease	LAC12	
KLMA_30011	Beta-glucosidase	-	Cellobiose
KLMA_20184	Endo-1,3(4)-beta-glucanase 1	DSE4	1,3-β-D-Glucan
KLMA_50517	Endo-1,3(4)-beta-glucanase 2	ACF2	
KLMA_10518	Inulinase	INU1	Sucrose, raffinose, inulin
KLMA_40102	Alcohol dehydrogenase 1	ADH1	
KLMA_40220	Alcohol dehydrogenase 2	ADH2	
KLMA_80306	Alcohol dehydrogenase 3	ADH3	
KLMA_20005	Alcohol dehydrogenase 4a	ADH4a	
KLMA_20158	Alcohol dehydrogenase 4b	ADH4b	
KLMA_40624	Alcohol dehydrogenase	ADH	
KLMA_80339	Alcohol dehydrogenase 6	ADH6	

NAD, nicotinamide adenine dinucleotide; NADP, nicotinamide adenine dinucleotide phosphate.

Chromosomal segments including 4,277 genes, which retain the ancestral gene groupings, were found between *K. marxianus* and *K. lactis* (Additional file 1: Figure S2). The average of mapped segments to the chromosome is 57.9%, and the *K. marxianus* chromosome best covered by *K. lactis* chromosomal segments is chromosome 6, with 60.8% coverage.

### Ribosomal DNA (rDNA) copy number and thermotolerance

Optical mapping allowed us to estimate at least 140 copies of the rDNA gene as a cluster on chromosome 5 (Additional file 1: Figure S1), which occupies 67.5% (0.9 Mb) of the chromosome. To examine the relationship between the rDNA copy number and its thermotolerance among *K. marxianus* strains, strains exhibiting different growth at different temperatures (Additional file 1: Figures S3, S4) were subjected to a test to determine the copy number of rDNA (Additional file 1: Table S7). As a result, the rDNA copy number is not correlated to the thermotolerance of the yeast, and at least 31 copies of rDNA are sufficient to support its thermotolerance.

### Genes regulated under a static condition

The alteration of genome-wide gene expression was analyzed by transcription start site sequencing (TSS Seq) under four different conditions (Additional file 3): shaking condition in yeast extract peptone dextrose (YPD) medium at 30°C (30D) or 45°C (45D), static condition in YPD medium at 30°C (30DS), and shaking condition in yeast extract peptone xylose (YPX) medium at 30°C (30X) and was expressed as the ratio of 30DS/30D, 45D/30D, and 30X/30D using 30D as a control condition, which was evaluated by a statistical test (FDR < 0.05) (Additional file 1: Table S8) and summarized (Additional file 1: Figure S5).

The growth of *K. marxianus* under the static condition is much slower than that under the shaking condition [4,12]. Under the 30DS condition, there were 159 significantly upregulated genes (Additional file 1: Table S8), and the top-five significantly enriched GO terms were ribosome biogenesis, ribonucleoprotein complex biogenesis, rRNA processing, rRNA metabolic process, and noncoding RNA processing (Additional file 1: Table S9); their individual gene details are shown in Additional file 1: Table S10. Interestingly, 55% of upregulated gene products are located in the nucleus (Additional file 1: Figure S6), some of which are factors for ribosome biogenesis, ATP-dependent RNA helicases, RNA polymerases subunits, nucleolar complex proteins, components of exosome complex, DNA polymerase subunits, and chromatin assembly factor 1 subunits. Conversely, there were 154 significantly downregulated genes (Additional file 1: Table S8), and their most significantly enriched GO terms were ascospore formation, sexual sporulation, sexual sporulation resulting in the formation of a cellular spore, cell development, and reproductive process in single-celled organisms (Additional file 1: Tables S11, S12). The largest population consists of 43 genes for membrane proteins including 15 transporters for amino acids and other metabolites. Taken together, under the static condition, *K. marxianus* may increase the turnover of RNAs and proteins in addition to suppression of transporters and spore formation that depends on mitochondrial respiration activity [32].

Under the static condition, the oxygen level in cells may become low as cells proliferate so that the condition may affect oxygen-requiring biosynthetic pathways, such as those for heme, sterols, unsaturated fatty acids, pyrimidine, and deoxyribonucleotides [33]. As expected, almost all genes related to ergosterol biosynthesis, sterol biosynthesis, unsaturated fatty acids production, pyrimidine synthesis, and ribonucleotide reductase were largely upregulated under the 30DS condition (Additional file 1: Figure S7). However, unlike *S. cerevisiae*, the expression of *MDL1* for a putative mitochondrial heme carrier was not significantly altered [34]. In addition, *NPT1* for nicotinate phosphoribosyl transferase (Npt1) involved in the nicotinamide adenine dinucleotide phosphate (NAD) salvage pathway, *CYC7* for cytochrome *c* and *AAC* for ADP/ATP carrier in mitochondrial inner membrane were upregulated. The ADP/ATP carrier functions to exchange cytoplasmic adenosine diphosphate (ADP) for mitochondrial adenosine triphosphate (ATP) under aerobic conditions and vice versa under anaerobic conditions [33]. Taken together, these results suggest that the enhanced expression of most genes for several oxygen-dependent biosynthetic pathways or some genes related to the production and management of energy is crucial for the cellular metabolism of *K. marxianus* under the static condition. Notably, almost all genes described above were upregulated not only under the 30DS condition but also under the 45D condition, suggesting that cells suffer from oxygen deficiency under the two conditions.

**Figure 2:**
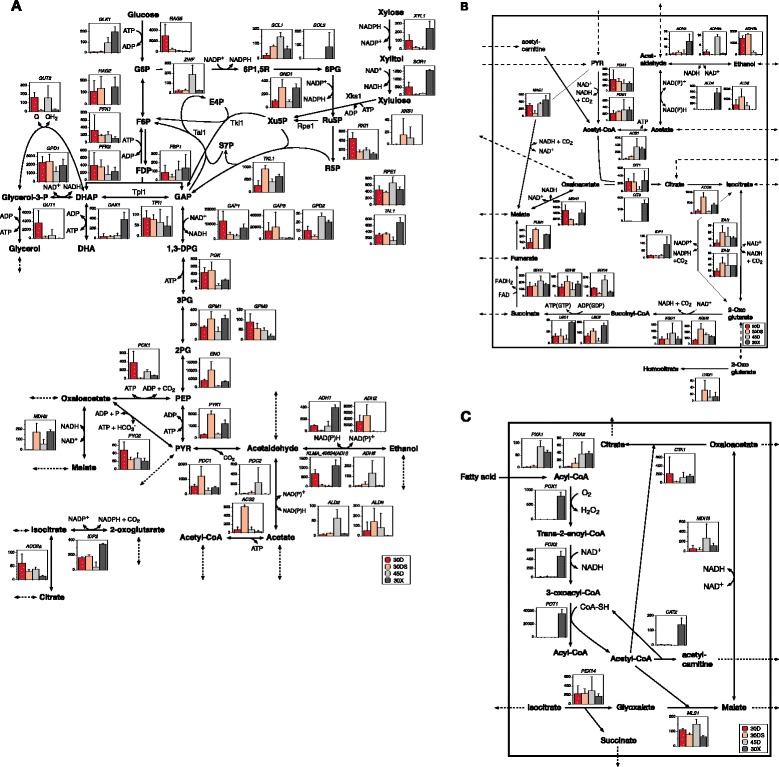
**Transcript abundance of genes related to central metabolic pathways under different conditions in** ***K. marxianus.*** The transcript abundance was determined by TSS analysis. The Y axis of each column graph shows the transcript abundance of each gene as TSS-tag ppm. Columns from left to right in each graph represent 30D (strong red), 30DS (soft orange), 45D (gray), and 30X (dark gray). Empty columns mean gene expression below the detectable level. **(A)** Central metabolic pathway. **(B)** Mitochondrial metabolic pathway. **(C)** Peroxisomal metabolic pathway. Abbreviations are as follows: G6P, glucose-6-phosphate; F6P, fructose-6-phosphate; FDP, fructose 1,6 bisphosphate; DHAP, dihydroxyacetone phosphate; DHA, dihydroxyacetone; GAP, glyceraldehyde-3-phosphate; 1,3-DPG, 1,3-bisphosphoglycerate; 3PG, 3-phosphoglycerate; 2PG, 2-phosphoglycerate; PEP, phosphoenolpyruvate; PYR, pyruvate; 6P1,5R, 6-phospho-D-glucono-1,5-lactone; 6PG, 6-phosphogluconate; Ru5P, ribulose-5-phosphate; R5P, ribose-5-phosphate; Xul5P, xylulose-5-phosphate; E4P, erythrose-4-phosphate; S7P, sedoheptulose-7-phosphate; NAD, nicotinamide adenine dinucleotide; NADP, nicotinamide adenine dinucleotide phosphate; Q, quinone; ATP, adenosine triphosphate; ADP, adenosine diphosphate.

**Figure 3:**
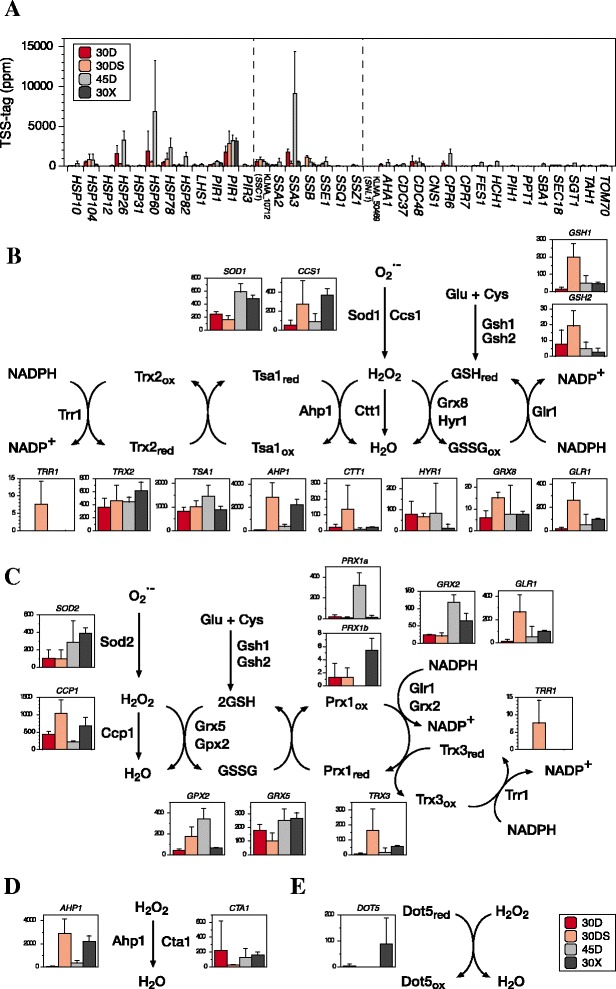
**Transcript abundance of genes related to oxidative stress response in** ***K. marxianus*** **. (A)** Transcript abundance of heat shock genes and related genes listed in Additional file 1: Table S18 is represented as TSS-tag ppm. Enzyme reactions to scavenge ROS in **(B)** cytoplasm, **(C)** mitochondria, **(D)** peroxisome, and **(E)** nucleus are shown. Each column in the graph shows the transcript abundance of each gene as described in Figure 2.

Metabolic changes were further analyzed by KEGG assignment. A number of genes for glycolysis after 1,3-bisphosphoglycerate, PPP, and tricarboxylic acid (TCA) cycle were relatively upregulated (Figure 2A,B). Enhanced PPP may provide nicotinamide adenine dinucleotide phosphate (NADPH) to cope with reactive oxygen species (ROS) generated under the condition, consistent with upregulation of ROS-scavenging genes (see Figure 3). Enhancement of expression of genes related to the pathway from 2-phosphoglycerate to acetyl-CoA via acetaldehyde may indicate the possibility that cells under a static condition tend to increase in acetyl-CoA and NADPH production in the process of oxidation of acetaldehyde. The oxaloacetate-malate shuttle may contribute to the oxidation of NADH in cytoplasm. Many genes for the respiratory chain were downregulated, though compensatorily most of the chaperone-coding genes for cytochrome *c* oxidase were upregulated (Additional file 1: Figure S8).

### Genes regulated under a high-temperature condition

To clarify the thermotolerant mechanism of *K. marxianus*, it is necessary to consider the expressional alteration of whole genomic genes at high temperature. Under the 45D condition, 508 genes were significantly downregulated (Additional file 1: Table S8) and the top-five significant GO terms were carbohydrate metabolic process, isoleucine biosynthetic process, small molecule metabolic process, monosaccharide metabolic process, and branched chain family amino acid biosynthetic process (Additional file 1: Tables S13, S14). Conversely, in 199 upregulated genes (Additional file 1: Table S8), the most significant GO terms were noncoding RNA processing, ribosome biogenesis, rRNA processing, ribonucleoprotein complex biogenesis, and rRNA metabolic process (Additional file 1: Tables S15, S16), and 45 genes were related to translation, transcription, DNA replication and repair, and protein and RNA degradations. Interestingly, *LYS21* for homocitrate synthase, which is linked to the key process of DNA damage repair in a nucleus [35] in addition to its involvement in lysine biosynthesis in the cytoplasm, was upregulated. Several genes for homologous recombination and nonhomologous end joining, which function in the repair of DNA double-stranded breaks, were also upregulated (Additional file 1: Figure S9). Therefore, it is assumed that *K. marxianus* copes with high temperatures by reducing central metabolic activities and reinforcing the synthesis and degradation of proteins and DNA repair.

In further analysis of the subcellular localization of products (Additional file 1: Figure S6), 21% of all upregulated genes are located in the nucleolus or nucleus (Additional file 1: Table S17), and are related to 18S rRNA preprocessing, 60S ribosomal subunit biogenesis, transcription process, and pre-rRNA processing (Additional file 1: Tables S15, S16). Conversely, products of several downregulated genes exist in the nucleolus, which are involved in 40S ribosomal subunit biogenesis and pre-18S rRNA processing (Additional file 1: Tables S13, S14). Notably, seven genes for mitochondrial ribosome subunits were downregulated. In addition, genes for DNA repair in nuclei and mitochondria, including a DNA damage sensor or chromosome transmission fidelity protein, were significantly upregulated (Additional file 1: Table S16).

Genes for glycolysis were remarkably downregulated, except for *GLK1* and *FBP1*, under the 45D condition (Figure 2A), which is consistent with relatively slow growth speed and low ethanol productivity at high temperatures [4]. Conversely, *ZWF* and *SOL1* in PPP in addition to *GLK1* were upregulated, indicating an increase in NADPH amount. In TCA cycle, downregulation of *CIT1*, *LSC2*, *FUM1*, and *MDH1* and upregulation of *IDH1*, *IDH2*, *KGD1*, and *KGD2* were found, which might lead to the accumulation of intermediates (Figure 2B and Additional file 1: Table S16). Most mitochondrial genomic genes were selectively expressed (Additional file 1: Figure S8). Additionally, some genes for succinate dehydrogenase (Sdh) and *bc*1 complex were upregulated, whereas some genes for NADH dehydrogenase (Ndh), Coenzyme Q biosynthesis, and cytochrome *c* oxidase including heme *a* synthesis were downregulated (Additional file 1: Figure S8). These findings allow us to speculate that *K. marxianus* scavenges H_2_O_2_ by the pathway of Sdh-*bc*1 complex-cytochrome *c* peroxidase and prevents the production of ROS by reduction of gene expression for Ndh and Coenzyme Q biosynthesis at high temperatures.

Heat shock proteins (Hsps) and chaperones are expected to be crucial for survival at high temperatures. The transcription of *HSP26*, *HSP60*, *HSP78*, *HSP82*, *SSA3*, and *CPR6* was enhanced under the 45D condition (Figure 3A and Additional file 1: Table S18), suggesting that both mitochondrial and cytoplasmic compartments need such Hsps at that temperature.

### Genes regulated under a xylose-utilizing condition

Under the 30X condition, the top-five significant GO terms of significantly upregulated genes were fatty acid catabolic process, monocarboxylic acid catabolic process, cellular lipid catabolic process, fatty acid β-oxidation, and fatty acid oxidation (Additional file 1: Tables S19, S20). Conversely, the most significant GO terms of significantly downregulated genes were α-amino acid metabolic process, carboxylic acid metabolic process, lysine biosynthetic process, small molecule biosynthetic process, and oxoacid metabolic process (Additional file 1: Tables S21, S22). Therefore, the 30X condition may stimulate the degradation of lipid in peroxisome and keep a low level of amino acid synthesis, which is consistent with the slow growth of the yeast in xylose [4].

**Figure 4:**
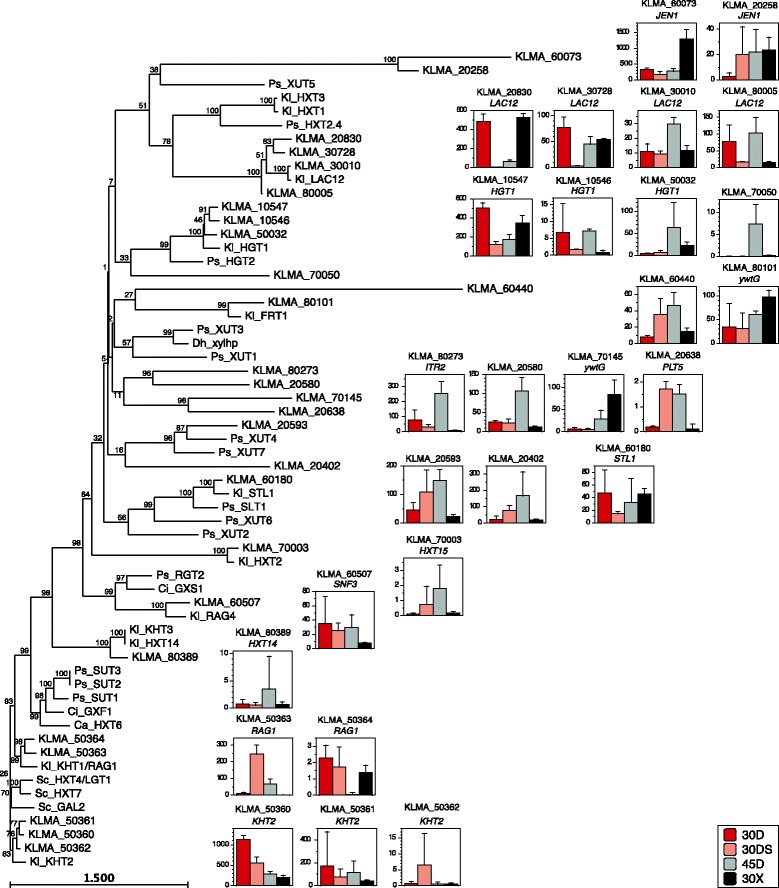
**Phylogenetic tree of predicted sugar transporters with transcript abundance in** ***K. marxianus*** **.** The phylogenetic tree was constructed with amino acid sequences of predicted sugar transporters by the neighbor-joining algorithm using CLC Sequence Viewer. Each column in the graph shows the transcript abundance of each gene as described in Figure 2. The accession numbers of amino acid sequences of sugar transporters are as follows: Ps_HGT2, XP_001382755; Sc_HXT7, NP_010629; Sc_GAL2, NP_013182; Dh_xylhp, AY347871; Ci_GXS1, GN107181; Ci_GXF1, GN107179; Ps_SUT1, XP_001387898; Ps_SUT2, XP_001384295; Ps_SUT3, XP_001386019; Ps_HXT2.4, XP_001387757; Ps_XUT1, XP_001385583; Ps_XUT2, XP_001387242; Ps_XUT3, XP_001387138; Ps_XUT4, XP_001386715; Ps_XUT5, XP_001385962; Ps_XUT6, XP_001386589; Ps_XUT7, XP_001387067; Ps_RGT2, XP_001386588; Ps_SLT1, XP_001383774; Kl_KHT1/RAG1, XP_453656; Kl_KHT2, GN107317; Kl_KHT3, XP_454897; Kl_FRT1, XP_454356; Kl_HGT1, XP_451484; Kl_HXT1, XP_455078; Kl_HXT14, XP_454897; Kl_HXT2, XP_453960; Kl_HXT3, XP_453088; Sc_HXT4/LGT1, NP_011960; Kl_STL1, XP_456249; Kl_RAG4, XP_455315; Kl_LAC12, XP_452193; Ca_HXT6, XP_719472.

The phylogenetic tree of sugar transporters revealed that KLMA_50360, KLMA_50361, KLMA_50362, KLMA_50363, and KLMA_50364 share high similarity with xylose transporters predicted in *S. stipitis* (Figure 4) [21]. Unlike *S. stipitis*, however, these genes were not induced specifically under the 30X condition. Genes of several transporters exhibited specific induction under the 30X or 30D condition, suggesting that KLMA_60073, KLMA_80101, and KLMA_70145 are involved in xylose uptake.

Genes for the initial catabolism of xylose, PPP, the conversion of PEP to ethanol, the mitochondrial conversion of acetaldehyde to acetyl-CoA and TCA cycle were relatively upregulated (Figure 2A,B). A part of the ethanol produced in the cytoplasm may thus be consumed by conversion to acetyl-CoA in mitochondria through the ethanol-acetaldehyde shuttle followed by TCA cycle, which is consistent with low ethanol productivity in xylose medium [4]. In contrast, *ADH2* and *ADH4b* for alcohol dehydrogenases, *GUT1* for glycerol kinase and *GUT2* for the glycerol-3-phosphate shuttle were downregulated. TSS results also indicate the generation of acetyl-CoA from the fatty acids through the peroxisomal β-oxidation pathway (Figure 2C), indicating the possibility that fatty acids could be a subsidiary intracellular carbon source in xylose medium. Such supply of acetyl-CoA in xylose medium might result in more NADH production and generate more ATP, which is required for phosphorylation of xylulose and dihydroxyacetone. Additional ATP may also be supplied as a result of the *DAK1* upregulation in the cytoplasm (Figure 2A).

## Discussion

As the first step to understanding the genetic basis of the highly efficient yeast *K. marxianus*, complete genome sequence and transcriptome analyses of its most thermotolerant strain were performed. The former analysis revealed many gene resources for the ability to assimilate various sugars in addition to species-specific genes. The latter clarified the molecular basis of attractive traits of the yeast. All information obtained here about the yeast will be useful for fundamental and applied research for innovative applications.

The thermotolerance as an attractive trait was investigated under the 45D condition, which revealed that *K. marxianus* seems to drastically change metabolic pathways from those under the 30D condition, that is, the enhancement of PPP and the attenuation of TCA cycle after the fumarate-producing step. The changes lead to the speculation that the former provides NADPH for scavenging ROS and that the latter deals with H_2_O_2_ via the electron transfer from Sdh to cytochrome *c* peroxidase (Figures 2 and 3). Consistent with these conjectures, a higher temperature generates more ROS, which causes DNA damage [36]. Notably, the findings of the upregulation of genes for DNA double-stranded break repair and removal of uracil in DNA molecules suggest the occurrence of enhancement of double-stranded break or deamination of cytosines in DNA at high temperatures. In addition, ATP synthesis via oxidative phosphorylation may be greatly reduced due to the repression of *ATP3* for the gamma subunit of ATP synthase. The existence of additional strategies is guessed from the TSS analysis data for survival at high temperature: alteration of ribosome biogenesis including pre-rRNA processing presumably for stable and efficient protein synthesis, reduction of mitochondrial ribosome biogenesis probably for saving energy, reinforcement of checkpoints of DNA replication and spindle assembly, minimization of electron leakage in respiratory chain by reduction of NADH dehydrogenase, and Coenzyme Q or enhanced expression of Hsps and chaperones. Taken together, the thermotolerance of *K. marxianus* is likely achieved by systematic mechanisms consisting of various strategies. Especially, the yeast would mainly acquire ATP from glycolysis rather than TCA cycle at high temperatures, which could prevent the generation of ROS by minimization of mitochondrial activity.

Under a static condition, the growth and ethanol production of *K. marxianus* were low compared to those under a shaking condition [4], probably due to low ATP yield in mitochondria, which may be related to the enhanced expression of *AAC. K. lactis* bearing a null mutation of *AAC2* exhibits growth defect on glycerol, galactose, maltose, and raffinose [37]. In addition, the expression of *RAG5* for hexokinase was relatively low. In *K. lactis*, *RAG5* mutations [38] abolish the expression of *RAG1* for a low-affinity glucose transporter [39] and decrease the level of the 2.0-kb mRNA species of *HGT1* for a high-affinity glucose transporter [40]. Furthermore, NADH would be accumulated in the cytoplasm because of the downregulation of *GAP1* for glyceraldehyde-3-phosphate dehydrogenase. Contrarily, respiratory genes kept their transcriptional levels similar to those under the 30D condition (Additional file 1: Figure S8). Such situations may raise reactive oxygen species from the respiratory chain to cause oxidative stress. Consistently, cytoplasmic oxidative stress response genes were relatively strongly expressed (Figure 3B), especially glutathione-related genes depicted at the cytoplasm side were highly induced. Almost *HSP*s, however, were not upregulated under the 30DS condition as under the 30D condition (Figure 3A). These metabolic activities may lead the low level of cell proliferation under a static condition.

Regulation of gene expression in response to the level of oxygen is achieved via several transcription factors. Under a static condition, *K. marxianus* seems to increase glucose metabolism and shift to fermentation, implying a connection between the oxygen- and glucose-sensing pathways. In *S. cerevisiae* and *K. lactis*, the transcription factor Hap1 mediates the induction of genes involved in their respiration, lipid metabolism, and oxidative stress response [11]. *K. lactis* Hap1 negatively regulates fermentation [41]. In contrast, the *HAP1* expression in *K. marxianus* was upregulated under the 30DS condition, and consistently, several genes related to ATP synthase and chaperones for respiratory chain components were upregulated (Additional file 1: Figure S8). These lines of evidence and the enhanced expression of genes for glycolytic pathway suggest differences in regulation of oxygen-responsive genes from those in *S. cerevisiae* and *K. lactis*.

The oxidative stress-response genes were found to be highly induced under the three conditions tested (Figures 2C and 4B,C,D,E), indicating that ROS is accumulated in the cytoplasm, mitochondria, and peroxisome under the 30DS and 30X conditions and in the cytoplasm and mitochondria under the 45D condition. Notably, Ahp1 in addition to Cta1 and Dot5 may be responsible for H_2_O_2_ detoxification in the peroxisome and nucleus, respectively.

Xylose assimilation capability as the second trait was examined under the 30X condition. It is known that *K. marxianus* tends to suffer from cofactor imbalance in xylose medium [42,43], and thus, its growth strongly depends on mitochondrial respiratory activity [44]. Interconvertibility of NAD^+^ species for maintaining the redox balance is essential for growth efficiency and metabolite excretion [45], but the yeast is incapable of directly converting NAD^+^ and NADPH into NADP^+^ and NADH owing to lack of transhydrogenases [46]. Instead, redox-balancing mechanisms between the cytoplasm and mitochondria are probably used to resolve the NADH/NADPH imbalance. Reoxidation of cytosolic NADPH and its strong connection to oxidative stress in *K. lactis* have been reported [47,48]. In *S. cerevisiae*, five cytosolic-mitochondrial redox shuttles have been proposed [49]. Of these, genes for enzymes related to ethanol-acetaldehyde, citrate-oxoglutarate, and oxaloacetate-malate shuttles were relatively upregulated under the 30X condition (Figures 2A,B). The GABA shunt from 2-oxoglutarate to succinate that has been proposed in *S. cerevisiae* and *S. stipitis* [21,50] may not be so important due to the low expression of related genes (Additional file 1: Figure S10), suggesting that *K. marxianus* uses different shuttles for resolving the cofactor imbalance from those of the two yeasts. In addition to TCA cycle intermediates by these shuttles, acetyl-CoA might be transferred from peroxisome on the basis of TSS data. Eventually, mitochondrial activity may be enhanced, which tends to increase the leakage of electrons to generate ROS, which is consistent with elevation of expression of oxidative stress-response genes.

The last trait is efficient protein productivity, which meets the demand for fast growth and high yield biomass [51]. The yeast has been exploited as a cell factory to obtain valuable enzymes, showing retention of activity over a large temperature range [52]. TSS results under the 30D and 30X conditions reveal high expression of *INU1* for inulinase, which is useful for the production of recombinant proteins in culture medium, as described in previous studies [26,27,53]. These useful characteristics may allow simultaneous production of ethanol and valuable proteins, thus, reducing the cost of ethanol production.

## Conclusions

The complete sequences of *K. marxianus* DMKU 3-1042 nuclear and mitochondrial genomes have been determined, which reveal many genes for the cells to cope with a high temperature and to assimilate a wide variety of sugars including xylose and arabinose in addition to species-specific genes. The present study thus provides the molecular basis of attractive traits of the yeast for industrial applications including high-temperature fermentation, and information of its gene resources and transcriptome data, which are particularly useful for fundamental and applied researches for innovative applications.

## Methods

### Strains, media, and culture conditions

The yeast strain used in this work was *K. marxianus* DMKU 3-1042 strain, which has been deposited in the NITE Biological Resource Center (NBRC) under the deposit numbers NITE BP-283 and NBRC 104275. Media used were YP (1% *w*/*v* yeast extract and 2% *w*/*v* peptone) supplemented with one of two different carbon sources: YPD, with 2% *w*/*v* glucose, or YPX, with 2% *w*/*v* xylose.

### Genome sequencing, assembly, and annotation

Two plasmid libraries with average insert sizes of 3 and 5 kb were generated in pTS1 (Nippon Gene, Tokyo, Japan) and pUC118 (Takara Bio. Inc., Otsu, Shiga, Japan) plasmid vectors, respectively, while a fosmid library with an average insert size of 40 kb was constructed in pCC1FOS (EPICENTRE, Illumina Inc., San Diego, CA, USA). Shotgun sequencing was performed on an ABI 3730*xl* DNA Analyzer (Applied Biosystems Co., Thermo Fisher Scientific, Inc., Foster City, CA, USA). Gaps were closed by the sequencing of gap-spanning PCR products. Telomeric regions were further analyzed by transposon-insertion sequencing of corresponding fosmid clones with Template Generation System II (Finnzymes, Thermo Fisher Scientific, Inc., Foster City, CA, USA). Genome assemblies were validated by optical mapping (OpGen, Gaithersburg, MD, USA). The genome was finally assembled into nine ungapped contigs corresponding to eight chromosomes and a mitochondrion with and average coverage of 11.1x. The mean error rate was estimated to be less than 6 × 10^−8^.

The rRNA-encoding regions were identified by a BLASTN program using the ribosomal sequences of *K. marxianus*, while the tRNA-coding regions were predicted by the ARAGORN program [54]. Proteins-coding gene prediction was performed by combining the Glimmer 3.02 program with a self-training dataset and six-frame prediction by using *in silico* Molecular Cloning (in silico biology, Inc., Yokohama, Kanagawa, Japan) and manual identification [55,56]. Intron prediction was performed by the AUGUSTUS program using the coding DNA sequences (CDSs) of *Ashbya gossypii* as the reference for pattern learning and manual correction of each CDS [57,58]. Functional annotation of the predicted CDSs was performed by BLASTP searching against the nonredundant (nr) database [59] with an E-value threshold of 10^−10^. Protein domains were predicted using the InterProScan program against various domain libraries (Prints, Prosite, PFAM, ProDom, SMART). Protein functions were assigned by KOG database [60]. Assignment of UniProt number was performed by a BLASTP program against the UniProt database [61] with an E-value threshold of 10^−10^. UniProt gene name, cellular localization, and Gene Ontology were assigned based on UniProt database information. The assignment of KO number of KEGG Orthology was performed by the KAAS program [62,63]. Individual annotations were then summarized according to KEGG Orthology and KEGG metabolic pathways.

### Transcriptome analysis in *K. marxianus*

Cells grown in 100-ml Erlenmeyer flasks in 30 ml YPD medium at 30°C, 160 rpm for 18 h were inoculated at the initial OD_660_ of about one into sequential batch culture, which was conducted in 300-ml Erlenmeyer flasks with 100 ml YPD at 30°C or 45°C under the shaking condition (30D and 45D) or the static condition at 30°C (30DS) or with 100 ml YPX at 30°C under the shaking condition (30X). The cells were further cultivated under each condition for 6 h and immediately subjected to RNA isolation. Each culture condition for TSS Seq experiments is under the same condition as performed previously [4]. Total RNA from cells was isolated by the hot phenol method [64] and was purified using an RNeasy Midi Kit (QIAGEN, Hilden, Germany) with RNase free DNase I (QIAGEN) according to the manufacturer’s instructions. Transcriptional starting site (TSS) Seq analysis was performed using the extracted total RNA, providing precise information on TSSs and their expression levels in a high-throughput manner [65]. TSS-tag counts were divided by the total number of uniquely and perfectly (with no mismatch) mapped TSS-tags to calculate TSS-tag ppm (parts per million). Experiments on each culture condition and TSS Seq analysis were performed in triplicate. Statistical testing was performed by edgeR of R package using the TSS-tag counts data of TSS analysis with a cutoff value as a false discovery rate smaller than 0.05 indicating significantly changed genes [66]. The GO enrichment test was performed by topGO of R package [67]. The TSS data was submitted to the Gene Expression Omnibus database (GEO, http://www.ncbi.nlm.nih.gov/geo/) under the accession number GSE66600.

### Nucleotide sequence accession numbers

The complete genome sequence of *K. marxianus* DMKU 3-1042 has been deposited in DDBJ/EMBL/GenBank under accession no. AP012213-AP012221.

### Quantitative real-time PCR (qPCR) analysis

Primers for qPCR were designed by using Primer Express software version 3.0 (Applied Biosystems Co.). A pair of primers, Km-rDNA-F1: 5’-GATCGGGTGGTGTTTTTCTTATG-3’ and Km-rDNA-R1: 5’-TCCCCCCAGAACCCAAAG-3’, was designed to amplify the 18S rDNA gene. The reaction produced a 71-bp PCR product. Probe for 18S rDNA, Km-rDNA-probe1: 5’-CCCACTCGGCACCTTACGAGAAATCA-3’ was labeled at the 5’-end with 6-carboxyfluorescein (FAM) as a reporter and at the 3’-end with dihydrocyclopyrroloindole tripeptide minor groove binder (MGB) as a quencher. Real-time PCR was performed using a TaqMan® Universal Master Mix II (Applied Biosystems Co.) and Applied Biosystems 7300 Real-Time PCR system (Applied Biosystems Co.). The 18S rDNA was amplified using genomic DNA isolated from 10 strains of *K. marxianus*. The *C*_*T*_ value was determined by the instrument’s software and adjusted manually as necessary. Concentration and DNA quality were measured by using Qubit™ dsDNA HS Assay Kits (Invitrogen Ltd., Paisley, UK) with Qubit® Fluorometer and by gel electrophoresis and converted to the number of copies by using the molecular weight of the DNA. The equation *C*_*T*_ = *m* (log quantity) + b from the equation for a line (y = mx + b) was constructed by plotting the standard curve of log quantity versus its corresponding *C*_*T*_ value. The 18S rDNA copy numbers were determined by the absolute quantitation method, by which total copies were first calculated using the following equation: total 18S rDNA copies = 10^([*CT* − b]/*m*)^. The number of 18S rDNA copies per genome was then determined by the following equation: 18S rDNA copies per genome = (Total copies of 18S rDNA)/(Total copy of genomic DNA). The genome size of 11.0 Mb of *K. marxianus* DMKU 3-1042 was used for all calculations.

## Additional files

Additional file 1:
**Supplementary information.** A file containing 10 supplementary figures and 22 supplementary tables. Additional file 2:
**Gene annotation.** A file that contains locus tags and their corresponding products. Additional file 3:
**Transcriptome analysis results.** A file that contains locus tags, their corresponding products and TSS-tag count in part per million (ppm) as used under the different conditions described in the study.

## Abbreviations

1,3-DPG: 1,3-bisphosphoglycerate
2PG: 2-phosphoglycerate
3PG: 3-phosphoglycerate
6P1,5R: 6-phospho-D-glucono-1,5-lactone
6PG: 6-phosphogluconate
ADP: Adenosine diphosphate
ATP: Adenosine triphosphate
DHA: Dihydroxyacetone
DHAP: Dihydroxyacetone phosphate
E4P: Erythrose-4-phosphate
F6P: Fructose-6-phosphate
FDP: Fructose 1,6 bisphosphate
G6P: Glucose-6-phosphate
GAP: Glyceraldehyde-3-phosphate
Hsp: Heat shock protein
HTF: High-temperature fermentation
KOG: Eukaryotic orthologous groups
NAD: Nicotinamide adenine dinucleotide
NADP: Nicotinamide adenine dinucleotide phosphate
NBRC: NITE Biological Resource Center
Ndh: NADH dehydrogenase
NR: Nonredundant
PEP: Phosphoenolpyruvate
PPP: Pentose phosphate pathway
PYR: Pyruvate
qPCR: quantitative real-time PCR
R5P: Ribose-5-phosphate
rDNA: ribosomal DNA
ROS: Reactive oxygen species
Ru5P: Ribulose-5-phosphate
S7P: Sedoheptulose-7-phosphate
Sdh: Succinate dehydrogenase
TSS Seq: Transcription start site sequencing
Xul5P: Xylulose-5-phosphate
