# Supplementary material for: Genetic basis of the highly efficient yeast Kluyveromyces marxianus: complete genome sequence and transcriptome analyses
Source: Biotechnol Biofuels. 2015 Mar 18;8:47. doi: 10.1186/s13068-015-0227-x (PMC4381506; doi:10.1186/s13068-015-0227-x)
Supplement: Additional file 1: — Supplementary information. A file containing 10 supplementary figures and 22 supplementary tables. [file 13068_2015_227_MOESM1_ESM.docx]

Supplementary Information

Genetic basis of the highly efficient yeast *Kluyveromyces marxianus*: complete genome sequence and transcriptome analyses

**Authors:** Noppon Lertwattanasakul^1,5,7^, Tomoyuki Kosaka^2,7^, Akira Hosoyama^3,7^, Yutaka Suzuki^4,7^, Nadchanok Rodrussamee^1,6^, Minenosuke Matsutani^2^, Masayuki Murata^1^, Naoko Fujimoto^1^, Suprayogi^1^, Keiko Tsuchikane^3^, Savitree Limtong^5^, Nobuyuki Fujita^3^ and Mamoru Yamada^1,2,§^

**Affiliation:**

^1^ Applied Molecular Bioscience, Graduate School of Medicine, Yamaguchi University, Ube 755-8505, Japan

^2^ Department of Biological Chemistry, Faculty of Agriculture, Yamaguchi University, Yamaguchi 753-8515, Japan

^3^ National Institute of Technology and Evaluation, Shibuya-ku, Tokyo 151-0066, Japan

^4^ Department of Medical Genome Sciences, the University of Tokyo, Chiba 277-8562, Japan

^5^ Department of Microbiology, Faculty of Science, Kasetsart University, Bangkok 10900, Thailand

^6^ Department of Biology, Faculty of Science, Chiang Mai University, Chiang Mai 50200, Thailand

^7^ These authors contributed equally to this study.

^§^ **Correspondence to:**

Mamoru Yamada, Ph.D.
Professor,

Applied Molecular Bioscience, Graduate School of Medicine, Yamaguchi University, Ube 755-8505, Japan and Department of Biological Chemistry, Faculty of Agriculture, Yamaguchi University, Yamaguchi 753-8515, Japan

Email: m-yamada@yamaguchi-u.ac.jp

**Figure S1** For each chromosome, the upper image represents data from optical mapping while the lower image depicts data from nucleotide sequencing. Asterisks indicate inverted regions. The green highlighting on Chromosome 5 represents the highly repetitive rDNA region.

**Figure S2** Conserved chromosomal segments found between *K. marxianus* and *K. lactis.* Complete genome sequence of *K. lactis* was mapped onto that of *K. marxianus*. Alignment against whole chromosomes was done using the Promer from the MUMmer package (Kurtz et al. *Genome Biology*, 5:R12, 2004). Color bars represent the *K. lactis* chromosomes: red, chromosome 1; blue, chromosome 2; green, chromosome 3; magenta, chromosome 4; light blue, chromosome 5; yellow, chromosome 6.

**A**

**B**

**C**

**D**

**Figure S3** Growth curve comparison among several *K. marxianus* strains under different temperature conditions. Strains tested are DMKU 3-1042, NBRC 1777, NBRC 0219, NBRC 0272, NBRC 0482, NBRC 0541, NBRC 0690, NBRC 1735, NBRC 1963 and NBRC 10005. Cells were grown in YPD medium at 40°C **(A)**, 45°C **(B)**, 46°C **(C)** and 47°C **(D)** under a shaking condition (160 rpm).

**A**

**B**

**C**

**Figure S4** Spot test of several *K. marxianus* strains. Cells were grown in YPD medium to about 10^7^ cells/ml, aliquots of 10-fold serial dilutions of cells were spotted onto YPD agar plates and the plates were incubated at 30°C **(A)**, 45°C **(B)** and 48°C **(C)** for 3 days.

**A**

**B**

**C**

**Figure S5** TSS analysis data summarized by logFC/logCPM plot of 30°C xylose shaking (30X)/30°C glucose shaking (30D) **(A)**, 30°C glucose static (30DS)/30D **(B)** and 45°C glucose shaking (45D)/30D **(C)**. logFC is legalistic value of fold change (FC). logCPM is legalistic value of count per million (CPM). Each spot indicates one gene. Red spots are genes that were significantly changed in expression. Yellow spots are outlier genes. Blue lines represent log_2_ = 1 or -1.

**A**

**B**

**Figure S6** Subcellular localization of products from significantly up-regulated or down-regulated genes under different conditions in *K. marxianus*. Total RNA was prepared from cells grown batch-wise on YPD or YPX medium under four conditions. **(A)** The up-regulated or down-regulated genes are classified according to subcellular localizations of their products. **(B)** The number in **(A)** is expressed as a percentage. The precise numbers and percentages of **(A)** and **(B)** are listed in Table S17. The remaining minor subcellular locations are combined and expressed as membrane (several locations) and non-membrane (several locations). Up and down indicate significantly up- and down-regulated genes, respectively, under these conditions compared to 30D. The subcellular localization of each gene product was assigned by UniProt and listed in Tables S10, S12, S14, S16, S20 and S22.

**Figure S7** Transcript abundance represented by TSS-tag ppm of each genes related to the oxygen-dependent biosynthetic pathways under different conditions in *K. marxianus.* Empty column means the gene expression below detectable level.

**Figure S8** Transcript abundance represented by TSS-tag ppm of each genes related to the ATP synthase, respiratory chain components and their chaperones under different conditions in *K. marxianus.* Empty column means the gene expression below detectable level.

**B**

**A**

**Figure S9** Transcript abundance represented by TSS-tag ppm of each genes related to homologous recombination and non-homologous end-joining under different conditions in *K. marxianus.* The transcript abundance by TSS-tag ppm of genes related to homologous recombination **(A)** and non-homologous end-joining **(B)** under different conditions were shown. Empty column means the gene expression below detectable level.

**Figure S10** Transcript abundance represented by TSS-tag ppm of each genes related to GABA shunt under different conditions in *K. marxianus.* Empty column means the gene expression below detectable level.

**Table S1** KOG assignment summary of *K. marxianus* genomic genes

| Total CDS | 4954 |
| --- | --- |
| assigned KOG | 3587 |
| assigned KOG percent (%) | 72.4 |
| assigned KOG function | 2893 |
| assigned KOG function percent (%) | 58.4 |
| KOG categories |  |
| Amino acid transport and metabolism | 179 |
| Carbohydrate transport and metabolism | 151 |
| Cell cycle control | 116 |
| Cell motility | 6 |
| Cell wall/membrane/envelope biogenesis | 33 |
| Chromatin structure and dynamics | 76 |
| Coenzyme transport and metabolism | 63 |
| Cytoskeleton | 60 |
| Defense mechanisms | 25 |
| Energy production and conversion | 175 |
| Extracellular structures | 6 |
| Inorganic ion transport and metabolism | 94 |
| Intracellular trafficking | 217 |
| Lipid transport and metabolism | 127 |
| Nuclear structure | 29 |
| Nucleotide transport and metabolism | 71 |
| Posttranslational modification | 331 |
| Replication | 137 |
| RNA processing and modification | 196 |
| Secondary metabolites biosynthesis | 60 |
| Signal transduction mechanisms | 251 |
| Transcription | 205 |
| Translation | 285 |
| Function unknown | 263 |
| General function prediction only | 431 |
| no hit | 1367 |

**Table S2** Unique genes in *K. marxianus*

| Locus_tag | Product |
| --- | --- |
| KLMA_10002 | hypothetical protein |
| KLMA_10003 | hypothetical protein |
| KLMA_10013 | hypothetical protein |
| KLMA_10017 | hypothetical protein |
| KLMA_10019 | hypothetical protein |
| KLMA_10040 | uncharacterized protein YDR524C-B |
| KLMA_10041 | hypothetical protein |
| KLMA_10061 | DUF3128 super family |
| KLMA_10067 | SCP super family |
| KLMA_10146 | Zn2/Cys6 DNA-binding domain |
| KLMA_10217 | proline-rich protein LAS17 |
| KLMA_10233 | hypothetical protein |
| KLMA_10242 | RRM |
| KLMA_10314 | silent chromatin protein ESC1 |
| KLMA_10355 | hypothetical protein |
| KLMA_10376 | ribosomal_L22 |
| KLMA_10423 | hypothetical protein |
| KLMA_10432 | NADB_Rossmann super family |
| KLMA_10443 | hypothetical protein |
| KLMA_10466 | erv26 super family |
| KLMA_10529 | hypothetical protein |
| KLMA_10574 | chromatin modification-related protein EAF7 |
| KLMA_10637 | hypothetical protein |
| KLMA_10652 | hypothetical protein |
| KLMA_10664 | smg4_UPF3 super family |
| KLMA_10676 | zinc finger |
| KLMA_10696 | GAL4 |
| KLMA_10701 | ribosomal_L13e super family |
| KLMA_10770 | hypothetical protein |
| KLMA_10773 | hypothetical protein |
| KLMA_10806 | hypothetical protein |
| KLMA_10829 | hypothetical protein |
| KLMA_10837 | hypothetical protein |
| KLMA_20001 | conserved hypothetical membrane protein |
| KLMA_20002 | flocculation protein FLO5 |
| KLMA_20055 | hypothetical protein |
| KLMA_20103 | hypothetical protein |
| KLMA_20104 | hypothetical protein |
| KLMA_20154 | uncharacterized protein YMR086W |
| KLMA_20163 | hypothetical protein |
| KLMA_20219 | BNI1-related protein 1 |
| KLMA_20261 | hypothetical protein |
| KLMA_20328 | hypothetical protein |
| KLMA_20349 | SWR1-complex protein 3 |
| KLMA_20369 | mediator of RNA polymerase II transcription subunit 15 |
| KLMA_20380 | hypothetical protein |
| KLMA_20393 | zinc finger protein STP4 |
| KLMA_20428 | flocculation protein FLO11 |
| KLMA_20441 | cell wall integrity and stress response component 4 |
| KLMA_20465 | chitin biosynthesis protein CHS5 |
| KLMA_20496 | hypothetical protein |
| KLMA_20538 | protein CAF130 |
| KLMA_20567 | hypothetical protein |
| KLMA_20572 | flocculation suppression protein |
| KLMA_20606 | hypothetical protein |
| KLMA_20625 | hypothetical protein |
| KLMA_20633 | hypothetical protein |
| KLMA_20689 | protein FYV8 |
| KLMA_20758 | protein DSE2 |
| KLMA_20782 | hypothetical protein |
| KLMA_20820 | hypothetical protein |
| KLMA_30002 | chol_sulfatase |
| KLMA_30003 | transposon Ty1-NL1 Gag polyprotein |
| KLMA_30005 | transposon Ty1-NL1 Gag polyprotein |
| KLMA_30007 | transposon Ty1-NL1 Gag polyprotein |
| KLMA_30020 | AP-1 accessory protein LAA1 |
| KLMA_30079 | centromere DNA-binding protein complex CBF3 subunit |
| KLMA_30115 | guanine nucleotide-binding protein subunit beta-like |
| KLMA_30116 | hypothetical protein |
| KLMA_30122 | DASH complex subunit HSK3 |
| KLMA_30178 | myosin tail region-interacting protein MTI1 |
| KLMA_30208 | c reductase complex |
| KLMA_30226 | cytochrome c oxidase copper chaperone |
| KLMA_30239 | plasma membrane proteolipid 3 |
| KLMA_30309 | protein SPA2 |
| KLMA_30317 | hypothetical protein |
| KLMA_30330 | homeodomain super family |
| KLMA_30368 | dentin matrix acidic phosphoprotein 1 |
| KLMA_30382 | nuclear localization sequence-binding protein |
| KLMA_30502 | putative guanine nucleotide-exchange factor SED4 |
| KLMA_30555 | conserved hypothetical transmembrane protein |
| KLMA_30609 | cell wall integrity sensor MID2 |
| KLMA_30667 | herpes_gp2 |
| KLMA_40001 | TY2B-F |
| KLMA_40049 | hansenula MRAKII killer toxin-resistant protein |
| KLMA_40079 | uncharacterized endoplamic reticulum membrane |
| KLMA_40106 | uncharacterized protein YDR210W |
| KLMA_40116 | hypothetical protein |
| KLMA_40117 | transcriptional regulator NRG1 |
| KLMA_40132 | transcription activator |
| KLMA_40149 | hypothetical protein |
| KLMA_40192 | translation machinery-associated protein 7 |
| KLMA_40200 | probable inorganic polyphosphate/ATP-NAD kinase |
| KLMA_40213 | MOG interacting and ectopic P-granules protein |
| KLMA_40227 | POU domain |
| KLMA_40240 | H/ACA ribonucleoprotein complex subunit 1 |
| KLMA_40299 | transposon Ty2-LR1 Gag-Pol polyprotein |
| KLMA_40304 | hypothetical protein |
| KLMA_40308 | hypothetical protein |
| KLMA_40366 | hypothetical protein |
| KLMA_40374 | negative regulator of RAS-cAMP pathway |
| KLMA_40377 | hypothetical protein |
| KLMA_40389 | conserved hypothetical membrane protein |
| KLMA_40446 | conserved hypothetical membrane protein |
| KLMA_40557 | PHO85 cyclin-8 |
| KLMA_40626 | flocculation protein FLO9 |
| KLMA_40631 | hypothetical protein |
| KLMA_50002 | choline-sulfatase conserved domain |
| KLMA_50003 | hypothetical protein |
| KLMA_50198 | nested antisense gene NAG1 |
| KLMA_50203 | verprolin |
| KLMA_50264 | 40S ribosomal protein S14 |
| KLMA_50320 | conserved hypothetical protein |
| KLMA_50324 | uncharacterized protein YPL250W-A |
| KLMA_50335 | transposon Ty1-H Gag-Pol polyprotein |
| KLMA_50343 | tubulin-specific chaperone C |
| KLMA_50397 | gti1_Pac2 super family conserved domain |
| KLMA_50409 | flo11 super family |
| KLMA_50423 | transposon Ty2-LR1 Gag-Pol polyprotein |
| KLMA_50425 | uncharacterized membrane protein YPR109W |
| KLMA_50456 | hypothetical protein |
| KLMA_50460 | mediator of RNA polymerase II transcription subunit 22 |
| KLMA_50515 | hypothetical protein |
| KLMA_50521 | hypothetical protein |
| KLMA_50529 | Hyaluronic acid-binding protein 4 |
| KLMA_50597 | dolichyl-diphosphooligosaccharide--protein glycosyltransferase |
| KLMA_50598 | subunit OST4 |
| KLMA_60035 | serine/threonine-protein kinase ste20 |
| KLMA_60043 | hypothetical protein |
| KLMA_60045 | conserved hypothetical membrane protein |
| KLMA_60069 | 40S ribosomal protein S0 |
| KLMA_60084 | mer2 super family protein |
| KLMA_60123 | RCR super family protein |
| KLMA_60124 | RCR super family protein |
| KLMA_60142 | N-glycosylation protein EOS1 |
| KLMA_60169 | hypothetical protein |
| KLMA_60215 | hypothetical protein |
| KLMA_60244 | protein RTM1 |
| KLMA_60269 | lysine-rich arabinogalactan protein 19 |
| KLMA_60270 | hypothetical protein |
| KLMA_60341 | SWI/SNF chromatin-remodeling complex subunit SNF5 |
| KLMA_60363 | hypothetical protein |
| KLMA_60368 | hypothetical protein |
| KLMA_60458 | mediator of RNA polymerase II transcription subunit |
| KLMA_70008 | conserved hypothetical protein |
| KLMA_70009 | flocculation protein FLO9 |
| KLMA_70019 | protein PET20 |
| KLMA_70030 | protein SIC1 |
| KLMA_70089 | conserved hypothetical membrane protein |
| KLMA_70107 | hypothetical protein |
| KLMA_70112 | conserved hypothetical protein |
| KLMA_70156 | conserved hypothetical protein |
| KLMA_70220 | conserved hypothetical protein |
| KLMA_70233 | uncharacterized protein YLR211C |
| KLMA_70275 | hypothetical protein |
| KLMA_70309 | conserved hypothetical protein |
| KLMA_70361 | conserved hypothetical protein |
| KLMA_70364 | conserved hypothetical protein |
| KLMA_70390 | suppressor protein SRP40 |
| KLMA_70391 | hypothetical protein |
| KLMA_70402 | succinate dehydrogenase assembly factor 2 |
| KLMA_70408 | zinc finger protein YPR022C |
| KLMA_70421 | DNA-directed RNA polymerase III subunit RPC8 |
| KLMA_70425 | uncharacterized protein MBB1 |
| KLMA_70428 | conserved hypothetical protein |
| KLMA_70453 | ice-structuring glycoprotein |
| KLMA_70458 | uncharacterized protein ywnB |
| KLMA_70461 | YKL222C |
| KLMA_70463 | hypothetical protein |
| KLMA_70464 | conserved hypothetical membrane protein |
| KLMA_80001 | hypothetical protein |
| KLMA_80053 | DNA-directed RNA polymerase II subunit RPB1 |
| KLMA_80069 | alpha-agglutinin |
| KLMA_80115 | transcriptional regulator CRZ1 |
| KLMA_80162 | vacuolar segregation protein 7 |
| KLMA_80187 | conserved hypothetical protein containing HMG-box super family |
| KLMA_80191 | meiotically up-regulated gene 70 protein |
| KLMA_80192 | conserved hypothetical membrane brotien |
| KLMA_80223 | hypothetical protein |
| KLMA_80289 | uncharacterized protein YBR108W |
| KLMA_80297 | general transcriptional corepressor CYC8 |
| KLMA_80304 | hypothetical protein |
| KLMA_80326 | hypothetical protein |
| KLMA_80378 | hypothetical protein |
| KLMA_80394 | protein KRE1 |
| KLMA_80423 | flocculation protein FLO9 |
| KLMA_80424 | uncharacterized oligopeptide transporter C1840.12 |
| KLMA_80428 | hypothetical protein |
| KLMA_80429 | hypothetical protein |
| KLMA_90005 | probable intron-encoded endonuclease aI5 |
| KLMA_90006 | Intron-encoded DNA endonuclease aI4 |
| KLMA_90008 | cytochrome c oxidase subunit 1 |
| KLMA_90009 | ATP synthase protein 8 |

**Table S3** KOG categories of genes specific for *K. marxianus*

| KOG category | Number |
| --- | --- |
| Carbohydrate transport and metabolism, Posttranslational modification, protein turnover, chaperones | 1 |
| Cell cycle control, cell division, chromosome partitioning | 1 |
| Cell cycle control, cell division, chromosome partitioning, Posttranslational modification, protein turnover, chaperones | 1 |
| Cell wall/membrane/envelope biogenesis | 1 |
| Chromatin structure and dynamics, Transcription | 1 |
| Cytoskeleton | 2 |
| Energy production and conversion | 3 |
| Function unknown | 5 |
| General function prediction only | 4 |
| Intracellular trafficking, secretion, and vesicular transport | 2 |
| Nuclear structure | 1 |
| Posttranslational modification, protein turnover, chaperones | 2 |
| RNA processing and modification | 3 |
| Signal transduction mechanisms | 11 |
| Signal transduction mechanisms, Carbohydrate transport and metabolism | 1 |
| Signal transduction mechanisms, Cytoskeleton | 2 |
| Signal transduction mechanisms, Lipid transport and metabolism | 1 |
| Transcription | 13 |
| Translation, ribosomal structure and biogenesis | 4 |
| non-categorized gene (KOG) | 134 |
| Total | 193 |

**Table S4** Genes shared only between *K. marxianus* and *K. lactis*

| Locus_tag | Product |
| --- | --- |
| KLMA_10001 | RVT_2 super family |
| KLMA_10014 | mating-type protein ALPHA1 |
| KLMA_10015 | mating-type protein ALPHA2 |
| KLMA_10016 | mating-type protein ALPHA3 |
| KLMA_10023 | hypothetical protein |
| KLMA_10026 | hypothetical protein |
| KLMA_10027 | hypothetical protein |
| KLMA_10028 | hypothetical protein |
| KLMA_10033 | hypothetical protein |
| KLMA_10056 | dnaQ_like_exo super family |
| KLMA_10057 | hypothetical protein |
| KLMA_10076 | protein PSP1 |
| KLMA_10100 | G1/S-specific cyclin CLN1 |
| KLMA_10104 | FH2 super family |
| KLMA_10119 | GYF super family |
| KLMA_10128 | hypothetical protein |
| KLMA_10141 | ANTH_AP180_CALM |
| KLMA_10142 | hypothetical protein |
| KLMA_10143 | mating-type protein A2 |
| KLMA_10144 | hypothetical protein |
| KLMA_10147 | RNase_H2_suC super family |
| KLMA_10160 | probable DNA-binding protein SNT1 |
| KLMA_10170 | SMC_prok_B |
| KLMA_10171 | CRT10 super family[ |
| KLMA_10174 | hypothetical protein |
| KLMA_10183 | zinc finger |
| KLMA_10197 | flocculation protein FLO5 |
| KLMA_10226 | hypothetical protein |
| KLMA_10232 | rhoGAP_fMSB1 |
| KLMA_10236 | SNF2_N |
| KLMA_10239 | hypothetical protein |
| KLMA_10246 | hypothetical protein |
| KLMA_10249 | cylicin-2 |
| KLMA_10277 | hypothetical protein |
| KLMA_10318 | RRM |
| KLMA_10327 | hypothetical protein |
| KLMA_10329 | RRM |
| KLMA_10346 | hypothetical protein |
| KLMA_10366 | hypothetical protein |
| KLMA_10367 | hypothetical protein |
| KLMA_10379 | RSC chromatin remodeling complex subunit RSC8 |
| KLMA_10430 | RRM |
| KLMA_10438 | hypothetical protein |
| KLMA_10469 | hypothetical protein |
| KLMA_10472 | hypothetical protein |
| KLMA_10502 | rtt102p super family |
| KLMA_10525 | atg31 super family |
| KLMA_10528 | pH-response regulator protein palI/RIM9 |
| KLMA_10538 | hypothetical protein |
| KLMA_10539 | inorganic pyrophosphatase |
| KLMA_10544 | glycoside hydrolase |
| KLMA_10555 | hypothetical protein |
| KLMA_10567 | hypothetical protein |
| KLMA_10579 | protein ZDS2 |
| KLMA_10584 | hypothetical protein |
| KLMA_10596 | hemocyanin |
| KLMA_10603 | increased rDNA silencing protein 4 |
| KLMA_10608 | hypothetical protein |
| KLMA_10646 | AFT super family |
| KLMA_10647 | hypothetical protein |
| KLMA_10697 | hypothetical protein |
| KLMA_10738 | PH domain-containing protein YHR131C |
| KLMA_10740 | esterase_lipase super family |
| KLMA_10743 | F-box protein COS111 |
| KLMA_10750 | hypothetical protein |
| KLMA_10756 | hypothetical protein |
| KLMA_10759 | hypothetical protein |
| KLMA_10779 | hypothetical protein |
| KLMA_10784 | MADS_MEF2_like |
| KLMA_10820 | hypothetical protein |
| KLMA_10826 | hypothetical protein |
| KLMA_10828 | hypothetical protein |
| KLMA_10835 | flocculation protein FLO5 |
| KLMA_20003 | conserved hypothetical membrane protein |
| KLMA_20010 | protein crtK |
| KLMA_20014 | hypothetical protein |
| KLMA_20016 | PCI super family |
| KLMA_20027 | hypothetical protein |
| KLMA_20036 | transcriptional activator HAP3 |
| KLMA_20059 | regulatory protein MIG1 |
| KLMA_20064 | hypothetical conserved protein |
| KLMA_20065 | DUF2410 super family |
| KLMA_20071 | DUP super family |
| KLMA_20072 | hypothetical conserved protein |
| KLMA_20073 | hypothetical conserved protein |
| KLMA_20080 | crossover junction endonuclease EME1 |
| KLMA_20117 | regulatory protein ADR1 |
| KLMA_20118 | rad9_Rad53_bind super family |
| KLMA_20128 | ribosome assembly protein 3 |
| KLMA_20144 | hap4 transcription factor |
| KLMA_20157 | probable serine/threonine-protein kinase HSL1 |
| KLMA_20160 | hypothetical protein |
| KLMA_20164 | hypothetical protein |
| KLMA_20166 | hypothetical protein |
| KLMA_20177 | hypothetical protein |
| KLMA_20178 | hypothetical protein |
| KLMA_20183 | conserved hypothetical protein |
| KLMA_20186 | conserved hypothetical protein |
| KLMA_20238 | uncharacterized protein YGL108C |
| KLMA_20266 | hypothetical protein |
| KLMA_20271 | zinc finger DNA binding domain |
| KLMA_20287 | hypothetical protein |
| KLMA_20302 | oligoribonuclease |
| KLMA_20303 | basic-leucine zipper (bZIP) transcription factor |
| KLMA_20315 | hypothetical protein |
| KLMA_20334 | hypothetical protein |
| KLMA_20338 | hypothetical protein |
| KLMA_20357 | sds3 super family |
| KLMA_20361 | hypothetical protein |
| KLMA_20365 | hypothetical protein |
| KLMA_20371 | hypothetical protein |
| KLMA_20381 | hypothetical protein |
| KLMA_20384 | hypothetical protein |
| KLMA_20416 | hypothetical protein |
| KLMA_20426 | p-loop NTPase super family |
| KLMA_20456 | conserved hypothetical protein |
| KLMA_20458 | putative uncharacterized protein YAL004W |
| KLMA_20477 | chromosome segregation ATPases |
| KLMA_20506 | hypothetical protein |
| KLMA_20516 | hypothetical protein |
| KLMA_20522 | pheromone-regulated membrane protein 3 |
| KLMA_20524 | bZIP_1 super family |
| KLMA_20533 | protein SAN1 |
| KLMA_20550 | DUF3020 super family |
| KLMA_20557 | SMC_prok_A |
| KLMA_20588 | hypothetical protein |
| KLMA_20599 | GAL4-like Zn2Cys6 binuclear cluster DNA-binding domain |
| KLMA_20601 | rho-type GTPase-activating protein 1 |
| KLMA_20641 | transcription activator MSS11 |
| KLMA_20668 | transcription initiation factor TFIID subunit 13 |
| KLMA_20684 | hypothetical protein |
| KLMA_20686 | hypothetical protein |
| KLMA_20697 | hypothetical protein |
| KLMA_20700 | serine/threonine-protein kinase PAK1 |
| KLMA_20704 | CBM_21 super family |
| KLMA_20705 | hypothetical protein |
| KLMA_20716 | ATPase |
| KLMA_20730 | hypothetical protein |
| KLMA_20746 | hypothetical protein |
| KLMA_20749 | uncharacterized protein YHR146W |
| KLMA_20754 | CRE-binding bZIP protein SKO1 |
| KLMA_20756 | bud neck protein 5 |
| KLMA_20772 | hypothetical protein |
| KLMA_20776 | hypothetical protein |
| KLMA_20780 | chaperone_DMP super family |
| KLMA_20819 | hypothetical protein |
| KLMA_20838 | hypothetical protein |
| KLMA_30004 | transposon Ty2-F/Ty2-GR2 Gag-Pol polyprotein |
| KLMA_30006 | transposon Ty2-F/Ty2-GR2 Gag-Pol polyprotein |
| KLMA_30008 | transposon Ty2-F/Ty2-GR2 Gag-Pol polyprotein |
| KLMA_30021 | mating-type protein A2 |
| KLMA_30022 | mating-type protein A1 |
| KLMA_30023 | protein SLA2 |
| KLMA_30036 | UDP-N-acetylenolpyruvoylglucosamine reductase |
| KLMA_30055 | bZIP_1 super family |
| KLMA_30058 | serine/threonine-protein kinase STE20 |
| KLMA_30077 | vacuolar protein sorting-associated protein 38 |
| KLMA_30121 | probable kinetochore protein SPC24 |
| KLMA_30129 | hypothetical protein |
| KLMA_30144 | negative regulator of sporulation MDS3 |
| KLMA_30166 | protein SIP4 |
| KLMA_30167 | hypothetical protein |
| KLMA_30186 | mediator of RNA polymerase II transcription subunit 2 |
| KLMA_30194 | phosphatidylserine decarboxylase proenzyme 2 |
| KLMA_30200 | central kinetochore subunit OKP1 |
| KLMA_30217 | mRNA-binding protein PUF3 |
| KLMA_30218 | ADIPOR-like receptor IZH3 |
| KLMA_30296 | protein BCK2 |
| KLMA_30315 | hypothetical protein |
| KLMA_30320 | glyco_transf_15 super family protein |
| KLMA_30338 | protein ICY2 |
| KLMA_30339 | ATP synthase subunit b |
| KLMA_30343 | central kinetochore subunit MCM21 |
| KLMA_30345 | hypothetical protein |
| KLMA_30351 | INCENP_ARK-bind super family protein |
| KLMA_30353 | hypothetical protein |
| KLMA_30354 | YTH1[COG5084] |
| KLMA_30356 | transcriptional regulatory protein LGE1 |
| KLMA_30369 | hypothetical protein |
| KLMA_30381 | hypothetical protein |
| KLMA_30399 | conserved hypothetical membrane protein |
| KLMA_30445 | conserved hypothetical protein |
| KLMA_30462 | protein NIP100 |
| KLMA_30474 | nucleolar protein NET1 |
| KLMA_30481 | glyoxalase super family protein |
| KLMA_30482 | glyoxalase super family protein |
| KLMA_30538 | hypothetical protein |
| KLMA_30544 | suppressor of mar1-1 protein |
| KLMA_30566 | transcription factor BAF1 |
| KLMA_30572 | meiosis induction protein kinase IME2/SME1 |
| KLMA_30576 | halotolerance protein 9 |
| KLMA_30614 | hypothetical protein |
| KLMA_30616 | transcriptional activator HAA1 |
| KLMA_30624 | conserved hypothetical transmembrane protein |
| KLMA_30630 | conserved hypothetical membrane protein |
| KLMA_30633 | conserved hypothetical membrane protein |
| KLMA_30635 | GAL4 |
| KLMA_30637 | bud site selection protein 8 |
| KLMA_30642 | conserved hypothetical transmembrane protein |
| KLMA_30651 | e3 SUMO-protein ligase SIZ1 |
| KLMA_30665 | ACBP super family protein |
| KLMA_30682 | nitrogen regulatory protein GLN3 |
| KLMA_30688 | conserved hypothetical protein |
| KLMA_30702 | hypothetical protein |
| KLMA_30718 | hypothetical protein |
| KLMA_30730 | hypothetical protein |
| KLMA_40017 | protein MSO1 |
| KLMA_40024 | probable serine/threonine-protein kinase YNR047W |
| KLMA_40058 | hypothetical protein |
| KLMA_40061 | cell wall protein YLR040C |
| KLMA_40077 | uncharacterized protein YMR124W |
| KLMA_40100 | meiotic activator RIM4 |
| KLMA_40108 | hypothetical protein |
| KLMA_40112 | serine/threonine-protein kinase ppk29 |
| KLMA_40127 | chaperone protein htpG |
| KLMA_40136 | protein CSF1 |
| KLMA_40146 | hypothetical protein |
| KLMA_40174 | uncharacterized cell wall protein YDR134C |
| KLMA_40187 | non-homologous end-joining protein 1 |
| KLMA_40201 | zinc finger protein YPR013C |
| KLMA_40204 | transcriptional regulatory protein UME6 |
| KLMA_40209 | hypothetical protein |
| KLMA_40211 | protein WHI3 |
| KLMA_40212 | solute carrier family 2 |
| KLMA_40215 | cell division control protein 13 |
| KLMA_40226 | pre-mRNA-splicing factor SPP381 |
| KLMA_40230 | hypothetical protein |
| KLMA_40232 | protein GZF3 |
| KLMA_40250 | hypothetical protein |
| KLMA_40260 | hypothetical protein |
| KLMA_40271 | zinc finger protein |
| KLMA_40272 | v-type proton ATPase catalytic subunit A |
| KLMA_40292 | hypothetical protein |
| KLMA_40311 | RNA polymerase II transcriptional coactivator SUB1 |
| KLMA_40314 | hypothetical protein |
| KLMA_40315 | hypothetical protein |
| KLMA_40317 | m-phase inducer phosphatase |
| KLMA_40326 | hypothetical protein |
| KLMA_40341 | hypothetical protein |
| KLMA_40345 | ubiquitin-like-specific protease 2 |
| KLMA_40348 | hypothetical protein |
| KLMA_40352 | hypothetical protein |
| KLMA_40358 | protein ASI2 |
| KLMA_40370 | hypothetical protein |
| KLMA_40383 | hypothetical protein |
| KLMA_40386 | hypothetical protein |
| KLMA_40421 | hypothetical protein |
| KLMA_40447 | weak acid resistance protein 1 |
| KLMA_40457 | zinc finger protein SFP1 |
| KLMA_40464 | beta tubulin |
| KLMA_40469 | hypothetical protein |
| KLMA_40484 | hypothetical protein |
| KLMA_40514 | hypothetical protein |
| KLMA_40541 | hypothetical protein |
| KLMA_40564 | hypothetical protein |
| KLMA_40582 | hypothetical protein |
| KLMA_40613 | zinc-regulated protein 8 |
| KLMA_40617 | putaive regulatory protein |
| KLMA_40621 | hypothetical protein |
| KLMA_40622 | hypothetical protein |
| KLMA_40629 | transposon Ty1-H Gag-Pol polyprotein |
| KLMA_40630 | transposon Ty2-F/Ty2-GR2 Gag-Pol polyprotein |
| KLMA_50004 | transposon Ty2-F/Ty2-GR2 Gag-Pol polyprotein |
| KLMA_50006 | peroxisome assembly protein 22 |
| KLMA_50010 | nuclear division defective protein 1 |
| KLMA_50027 | kinetochore protein SLK19 |
| KLMA_50064 | target of rapamycin complex 2 subunit BIT61,HbrB super family conserved domain |
| KLMA_50066 | hypothetical protein |
| KLMA_50078 | DNA polymerase epsilon subunit C |
| KLMA_50091 | COG5647 (Cullin |
| KLMA_50092 | hypothetical protein |
| KLMA_50097 | hypothetical protein |
| KLMA_50111 | protein SCD5 |
| KLMA_50114 | ras_like_GTPase super family |
| KLMA_50125 | sterol regulatory element-binding protein ECM22 |
| KLMA_50129 | probable phosphatidylinositol-4-phosphate 5-kinase MSS4 |
| KLMA_50138 | zinc finger |
| KLMA_50158 | chromatin structure-remodeling complex protein RSC14 |
| KLMA_50171 | THO complex subunit MFT1 |
| KLMA_50180 | hypothetical protein |
| KLMA_50199 | hypothetical protein |
| KLMA_50218 | protein MBR1 |
| KLMA_50238 | inositol hexakisphosphate kinase 1 |
| KLMA_50251 | J protein JJJ2 |
| KLMA_50252 | mitochondrial membrane protein FMP33 |
| KLMA_50256 | cyclin-dependent kinase inhibitor FAR1 |
| KLMA_50268 | hypothetical protein |
| KLMA_50275 | F-box domain |
| KLMA_50292 | conserved hypothetical membrane protein |
| KLMA_50298 | DSL1 super family conserved domain |
| KLMA_50300 | hypothetical protein |
| KLMA_50307 | predicted solute binding protein |
| KLMA_50330 | lactose regulatory protein LAC9 |
| KLMA_50359 | hypothetical protein |
| KLMA_50372 | uncharacterized protein YIR003W |
| KLMA_50386 | nuclear localization sequence-binding protein |
| KLMA_50392 | protein VAB2 |
| KLMA_50427 | pre-mRNA-processing factor 31 |
| KLMA_50451 | acyl-coenzyme A:6-aminopenicillanic-acid-acyltransferase 40 kDa form |
| KLMA_50461 | kinetochore-associated protein DSN1 |
| KLMA_50463 | conserved hypothetical membrane protein |
| KLMA_50479 | hypothetical protein |
| KLMA_50481 | uncharacterized protein YGR130C |
| KLMA_50494 | structure-specific endonuclease subunit SLX4 |
| KLMA_50509 | SH3 super family |
| KLMA_50512 | RNA polymerase I-specific transcription initiation factor RRN5 |
| KLMA_50541 | conserved hypothetical membrane protein |
| KLMA_50545 | conserved hypothetical membrane protein |
| KLMA_50555 | something about silencing protein 4 |
| KLMA_50561 | protein ATC1/LIC4 |
| KLMA_50594 | conserved hypothetical protein |
| KLMA_50599 | vacuolar import and degradation protein 27 |
| KLMA_50602 | PAPA-1 super family conserved domain |
| KLMA_50623 | protein BNI4 |
| KLMA_60001 | DUP super family |
| KLMA_60007 | flocculation protein FLO9 |
| KLMA_60016 | histone chaperone ASF1 |
| KLMA_60030 | hypothetical protein |
| KLMA_60044 | kelch repeat-containing protein 1 |
| KLMA_60049 | hypothetical protein |
| KLMA_60053 | spo12 super family protein |
| KLMA_60081 | mvb12 super family protein |
| KLMA_60097 | probable transcription factor HMS1 |
| KLMA_60105 | hypothetical protein |
| KLMA_60161 | hypothetical protein |
| KLMA_60162 | flavoprotein super family protein |
| KLMA_60170 | UBX domain-containing protein 4 |
| KLMA_60190 | splicing factor MUD2 |
| KLMA_60199 | H2A super family protein |
| KLMA_60202 | hypothetical protein |
| KLMA_60205 | conserved hypothetical phospatase and actin regulator 2 |
| KLMA_60207 | ISWI one complex protein 3 |
| KLMA_60217 | HLH[cd00083] |
| KLMA_60242 | E3 ubiquitin-protein ligase DMA2 |
| KLMA_60245 | mRNA-decapping enzyme subunit 2 |
| KLMA_60254 | H/ACA ribonucleoprotein complex non-core subunit NAF1 |
| KLMA_60266 | NDT80_PhoG super family protein |
| KLMA_60267 | protein FYV6 |
| KLMA_60273 | hypothetical protein |
| KLMA_60299 | GCR1_C super family protein |
| KLMA_60316 | uncharacterized transcriptional regulatory protein YKL038W |
| KLMA_60329 | HMGB-UBF_HMG-box containing protein |
| KLMA_60330 | hypothetical protein |
| KLMA_60332 | transcriptional activator/repressor MOT3 |
| KLMA_60345 | target of rapamycin complex 1 subunit TCO89 |
| KLMA_60352 | hypothetical protein |
| KLMA_60355 | ULP1-interacting protein 4 |
| KLMA_60367 | hypothetical membrane protein |
| KLMA_60404 | hypothetical protein |
| KLMA_60453 | hypothetical protein |
| KLMA_60456 | transcription factor NRM1 |
| KLMA_60460 | inheritance of peroxisomes protein 1 |
| KLMA_60466 | uncharacterized protein YMR206W |
| KLMA_60467 | hypothetical protein |
| KLMA_60490 | uncharacterized protein YDL129W |
| KLMA_60498 | uncharacterized protein YDL186W |
| KLMA_60525 | kilA-N super family protein |
| KLMA_60545 | hypothetical protein |
| KLMA_70004 | conserved hypothetical protein |
| KLMA_70023 | pheromone-regulated membrane protein 4 |
| KLMA_70035 | hypothetical protein |
| KLMA_70036 | hypothetical protein |
| KLMA_70062 | covalently-linked cell wall protein 14 |
| KLMA_70066 | conserved hypothetical membrane protein |
| KLMA_70085 | central kinetochore subunit MCM16 |
| KLMA_70090 | conserved hypothetical protein |
| KLMA_70094 | hypothetical protein |
| KLMA_70109 | bud site selection protein 4 |
| KLMA_70121 | assembly-complementing factor 4 |
| KLMA_70133 | retrograde regulation protein 3 |
| KLMA_70151 | 37S ribosomal protein MRP21 |
| KLMA_70164 | protein DAL82 |
| KLMA_70181 | hypothetical protein |
| KLMA_70187 | PHO85 cyclin-6 |
| KLMA_70198 | 54S ribosomal protein IMG2 |
| KLMA_70204 | conserved hypothetical protein |
| KLMA_70208 | hypothetical protein |
| KLMA_70221 | hypothetical protein |
| KLMA_70230 | hypothetical protein |
| KLMA_70242 | conserved hypothetical protein |
| KLMA_70267 | hypothetical protein |
| KLMA_70294 | hypothetical protein |
| KLMA_70303 | probable 6-phosphofructo-2-kinase/fructose-2,6-biphosphatase |
| KLMA_70323 | uncharacterized protein YOL036W |
| KLMA_70324 | transcriptional activator of sulfur metabolism |
| KLMA_70352 | mitotic spindle-associated protein SHE1 |
| KLMA_70356 | hypothetical protein |
| KLMA_70379 | nucleus export protein BRR6 |
| KLMA_70393 | hypothetical protein |
| KLMA_70394 | paxillin-like protein 1 |
| KLMA_70420 | conserved hypothetical membrane protein |
| KLMA_70430 | hypothetical protein |
| KLMA_70431 | hypothetical protein |
| KLMA_70460 | hypothetical protein |
| KLMA_80002 | transposon Ty2-LR1 Gag-Pol polyprotein |
| KLMA_80003 | transposon Ty2-DR2 Gag-Pol polyprotein |
| KLMA_80008 | conserved hypothetical protein |
| KLMA_80012 | conserved hypothetical protein |
| KLMA_80040 | aminoglycoside antibiotic sensitivity protein 3 |
| KLMA_80056 | conserved hypothetical protein |
| KLMA_80078 | conserved hypothetical protein |
| KLMA_80082 | hypothetical protein |
| KLMA_80136 | conserved hypothetical protein containing PIG-H super family |
| KLMA_80143 | hypothetical protein |
| KLMA_80164 | nucleoporin NUP159 |
| KLMA_80194 | protein PXR1 |
| KLMA_80209 | stress response protein NST1 |
| KLMA_80231 | [PSI+] induction protein 2 |
| KLMA_80257 | uncharacterized protein YKL054C |
| KLMA_80266 | hypothetical protein |
| KLMA_80274 | conserved hypothetical protein |
| KLMA_80284 | vacuolar import and degradation protein 24 |
| KLMA_80288 | hypothetical protein |
| KLMA_80303 | protein OPY2 |
| KLMA_80307 | hypothetical protein |
| KLMA_80320 | conserved hypothetical protein |
| KLMA_80338 | RNA-binding protein PIN4 |
| KLMA_80341 | transcriptional regulatory protein DOT6 |
| KLMA_80376 | uncharacterized protein YBL081W |
| KLMA_80402 | probable 26S proteasome complex subunit SEM1 |
| KLMA_80409 | autophagy-related protein 10 |
| KLMA_80426 | flocculation protein FLO5 |

**Table S5** Ortholog genes shared between *K. marxianus* and *O. parapolymorpha*, which are absent from *K. lactis*

| *K. marxianus* | *O. parapolymorpha* | product |
| --- | --- | --- |
| KLMA_10011 | HPODL_1713 | probable transporter SEO1 |
| KLMA_10012 | HPODL_1714 | uncharacterized protein C11D3.14c |
| KLMA_10060 | HPODL_2230 | selR super family |
| KLMA_10319 | HPODL_2009 | serine/threonine-protein kinase SKY1 |
| KLMA_10342 | HPODL_4082 | dihydrosphingosine 1-phosphate phosphatase LCB3 |
| KLMA_10592 | HPODL_3133 | enoate reductase 1 |
| KLMA_10631 | HPODL_3986 | 26S proteasome regulatory subunit RPN13 |
| KLMA_10824 | HPODL_1198 | Sit1p |
| KLMA_10834 | HPODL_0821 | repressible acid phosphatase |
| KLMA_30012 | HPODL_0089 | siderophore iron transporter mirA |
| KLMA_30276 | HPODL_2565 | siderophore iron transporter 1 |
| KLMA_40279 | HPODL_1232 | hypothetical protein |
| KLMA_40364 | HPODL_2244 | uncharacterized oxidoreductase YIR035C |
| KLMA_40424 | HPODL_0230 | uncharacterized MFS-type transporter C530.15c |
| KLMA_40491 | HPODL_3179 | homoserine dehydrogenase |
| KLMA_50087 | HPODL_2310 | sec sixty-one protein homolog |
| KLMA_50190 | HPODL_3676 | acyl-CoA-binding protein, ACBP |
| KLMA_50243 | HPODL_2732 | DNA replication complex GINS protein PSF1 |
| KLMA_50379 | HPODL_1677 | high affinity potassium transporter |
| KLMA_50391 | HPODL_2886 | prefoldin subunit 2 |
| KLMA_50447 | HPODL_2932 | vacuolar ATPase assembly integral membrane protein VMA21 |
| KLMA_60004 | HPODL_1024 | arylsulfotrans |
| KLMA_60156 | HPODL_2503 | actin-related protein 2/3 complex subunit 4 |
| KLMA_60402 | HPODL_3431 | pisatin demethylase |
| KLMA_60497 | HPODL_3764 | v-type proton ATPase catalytic subunit A |
| KLMA_60558 | HPODL_1717 | aminotriazole resistance protein |
| KLMA_70012 | HPODL_4148 | 1-aminocyclopropane-1-carboxylate oxidase |
| KLMA_70013 | HPODL_1025 | vacuolar basic amino acid transporter 2 |
| KLMA_70310 | HPODL_0880 | mitochondrial respiratory chain complexes assembly protein AFG3 |
| KLMA_80201 | HPODL_3639 | ubiquitin carboxyl-terminal hydrolase 15 |

**Table S6** Sugar transporters predicted in *K. marxianus*

| Locus_tag | Product | UniProt number | UniProt gene | KEGG Orthology |
| --- | --- | --- | --- | --- |
| KLMA_10546 | high-affinity glucose transporter | P49374 | HGT1 |  |
| KLMA_10547 | high-affinity glucose transporter | P49374 | HGT1 |  |
| KLMA_20258 | putative sialic acid transporter | P36035 | JEN1 | K08178 |
| KLMA_20402 | probable metabolite transport protein YBR241C | P38142 |  |  |
| KLMA_20580 | probable metabolite transport protein YDR387C | Q04162 |  |  |
| KLMA_20593 | uncharacterized mitochondrial outer membrane protein YDR381C-A | Q3E6R5 |  |  |
| KLMA_20638 | putative polyol transporter 2 | Q8VZ80 | PLT5 |  |
| KLMA_20830 | lactose permease | P07921 | LAC12 |  |
| KLMA_30010 | lactose permease | P07921 | LAC12 |  |
| KLMA_30728 | lactose permease | P07921 | LAC12 |  |
| KLMA_50032 | high-affinity glucose transporter | P49374 | HGT1 |  |
| KLMA_50360 | hexose transporter 2 | P53387 | KHT2 | K08139 |
| KLMA_50361 | hexose transporter 2 | P53387 | KHT2 | K08139 |
| KLMA_50362 | hexose transporter | P53387 | KHT2 |  |
| KLMA_50363 | low-affinity glucose transporter | P18631 | RAG1 | K08139 |
| KLMA_50364 | low-affinity glucose transporter | P18631 | RAG1 | K08139 |
| KLMA_60073 | carboxylic acid transporter protein homolog | P36035 | JEN1 | K08178 |
| KLMA_60180 | sugar transporter STL1 | P39932 | STL1 |  |
| KLMA_60440 | uncharacterized membrane protein YJR124C | P47159 |  |  |
| KLMA_60507 | high-affinity glucose transporter SNF3 | P10870 | SNF3 | K08139 |
| KLMA_70003 | hexose transporter HXT9 | P54854 | HXT15 |  |
| KLMA_70050 | probable metabolite transport protein YFL040W | P43562 |  |  |
| KLMA_70145 | conserved hypothetical membrane protein | C0SPB2 | ywtG |  |
| KLMA_80005 | high-affinity glucose transporter | P07921 | LAC12 |  |
| KLMA_80101 | conserved hypothetical protein containing the Major Facilitator Superfamily (MFS) domain | C0SPB2 | ywtG | K06609 |
| KLMA_80273 | myo-inositol transporter 2 | P30606 | ITR2 | K08150 |
| KLMA_80389 | hexose transporter HXT14 | P42833 | HXT14 |  |

**Table S7** rDNA copy number analysis of several *K. marxianus* strains

| Strain | rDNA copy number | Spot test | |
| --- | --- | --- | --- |
|  | (per genome) | 45°C | 48°C |
| DMKU 3-1042 | 205 | + + + + + | + + + + + |
| NBRC 0219 | 31 | + + + + + | + + + + + |
| NBRC 0272 | 56 | + + + | + |
| NBRC 0482 | 50 | + + + + + | + + + + |
| NBRC 0541 | 90 | + + | - |
| NBRC 0690 | 80 | + + + | - |
| NBRC 1735 | 88 | + | - |
| NBRC 1777 | 89 | + + + + + | + + + + + |
| NBRC 1963 | 276 | - | - |
| NBRC 10005 | 149 | + + | + |

+, the level of growth (see Figure S4)

-, no growth

**Table** **S8** Numbers of genes significantly changed in expression under different conditions (FDR < 0.05).

| Condition | 30DS/30D | 45D/30D | 30X/30D |
| --- | --- | --- | --- |
| Total gene number (4,839) | 313 | 707 | 168 |
| Up-regulated | 159 | 199 | 89 |
| Down-regulated | 154 | 508 | 79 |

**Table S9** GO terms enriched in significantly up-regulated genes under 30DS condition

| GO.ID | Term | Annotated  gene^a^ | Significant^b^ | Expected^c^ | P-value^d^ | Genes |
| --- | --- | --- | --- | --- | --- | --- |
| GO:0042254 | ribosome biogenesis | 270 | 59 | 9.67 | < 1e-30 | ALB1, BMS1, BRX1, BUD23, CAM1, CBF5, CGR1, CIC1, DBP10, DBP3, DBP7, DHR2, DIP2, DRS1, EFG1, ESF1, FUN12, IMP4, KRI1, LCP5, MAK5, MRT4, NMD3, NOC2, NOC3, NOG2, NOP1, NOP15, NOP2, NOP4, NOP56, NOP7, NSA1, NSA2, NSR1, PNO1, PUF6, PXR1, REI1, REX4, RLI1, RPF2, RPL8B, RRP14, RRP4, RRP42, RRP45, RRP5, RRP8, RSA3, SAS10, SDO1, SKI6, SPB1, SSF1, TIF6, TSR2, UTP15, UTP25 |
| GO:0022613 | ribonucleoprotein complex biogenesis | 317 | 59 | 11.35 | 4.7e-30 | ALB1, BMS1, BRX1, BUD23, CAM1, CBF5, CGR1, CIC1, DBP10, DBP3, DBP7, DHR2, DIP2, DRS1, EFG1, ESF1, FUN12, IMP4, KRI1, LCP5, MAK5, MRT4, NMD3, NOC2, NOC3, NOG2, NOP1, NOP15, NOP2, NOP4, NOP56, NOP7, NSA1, NSA2, NSR1, PNO1, PUF6, PXR1, REI1, REX4, RLI1, RPF2, RPL8B, RRP14, RRP4, RRP42, RRP45, RRP5, RRP8, RSA3, SAS10, SDO1, SKI6, SPB1, SSF1, TIF6, TSR2, UTP15, UTP25 |
| GO:0006364 | rRNA processing | 191 | 43 | 6.84 | 1.1e-24 | BMS1, BUD23, CBF5, CGR1, DBP10, DBP3, DBP7, DHR2, DIP2, EFG1, ESF1, FUN12, IMP4, KRI1, LCP5, MAK5, MRT4, NOC3, NOP1, NOP15, NOP2, NOP4, NOP56, NOP7, NSA1, NSA2, NSR1, PXR1, REX4, RLI1, RPF2, RRP14, RRP4, RRP42, RRP45, RRP5, SAS10, SKI6, SPB1, TIF6, TSR2, UTP15, UTP25 |
| GO:0016072 | rRNA metabolic process | 196 | 43 | 7.02 | 3.3e-24 | BMS1, BUD23, CBF5, CGR1, DBP10, DBP3, DBP7, DHR2, DIP2, EFG1, ESF1, FUN12, IMP4, KRI1, LCP5, MAK5, MRT4, NOC3, NOP1, NOP15, NOP2, NOP4, NOP56, NOP7, NSA1, NSA2, NSR1, PXR1, REX4, RLI1, RPF2, RRP14, RRP4, RRP42, RRP45, RRP5, SAS10, SKI6, SPB1, TIF6, TSR2, UTP15, UTP25 |
| GO:0034470 | ncRNA processing | 259 | 46 | 9.28 | 7.5e-22 | BMS1, BUD23, CBF5, CGR1, DBP10, DBP3, DBP7, DHR2, DIP2, EFG1, ELP6, ESF1, FUN12, IMP4, KRI1, LCP5, MAK5, MRT4, NOC3, NOP1, NOP15, NOP2, NOP4, NOP56, NOP7, NSA1, NSA2, NSR1, PUS4, PXR1, REX4, RLI1, RPF2, RRP14, RRP4, RRP42, RRP45, RRP5, SAS10, SKI6, SPB1, TIF6, TRM82, TSR2, UTP15, UTP25 |
| GO:0034660 | ncRNA metabolic process | 305 | 47 | 10.92 | 1.2e-19 | BMS1, BUD23, CBF5, CGR1, DBP10, DBP3, DBP7, DHR2, DIP2, EFG1, ELP6, ESF1, FUN12, ILS1, IMP4, KRI1, LCP5, MAK5, MRT4, NOC3, NOP1, NOP15, NOP2, NOP4, NOP56, NOP7, NSA1, NSA2, NSR1, PUS4, PXR1, REX4, RLI1, RPF2, RRP14, RRP4, RRP42, RRP45, RRP5, SAS10, SKI6, SPB1, TIF6, TRM82, TSR2, UTP15, UTP25 |
| GO:0044085 | cellular component biogenesis | 685 | 63 | 24.53 | 5.8e-15 | ALB1, BMS1, BRX1, BUD23, CAC2, CAM1, CBF5, CGR1, CIC1, DBP10, DBP3, DBP7, DHR2, DIP2, DRS1, EFG1, ESF1, FUN12, IMP4, KLMA_60262, KRI1, LCP5, MAK5, MRT4, NMD3, NOC2, NOC3, NOG2, NOP1, NOP15, NOP2, NOP4, NOP56, NOP7, NSA1, NSA2, NSR1, PNO1, PTP2, PUF6, PXR1, REI1, REX4, RLF2, RLI1, RPF2, RPL8B, RRP14, RRP4, RRP42, RRP45, RRP5, RRP8, RSA3, SAS10, SDO1, SKI6, SPB1, SSF1, TIF6, TSR2, UTP15, UTP25 |
| GO:0006396 | RNA processing | 405 | 47 | 14.5 | 1.6e-14 | BMS1, BUD23, BUD31, CBF5, CGR1, DBP10, DBP3, DBP7, DHR2, DIP2, EFG1, ELP6, ESF1, FUN12, IMP4, KRI1, LCP5, MAK5, MRT4, NOC3, NOP1, NOP15, NOP2, NOP4, NOP56, NOP7, NSA1, NSA2, NSR1, PUS4, PXR1, REX4, RLI1, RPF2, RRP14, RRP4, RRP42, RRP45, RRP5, SAS10, SKI6, SPB1, TIF6, TRM82, TSR2, UTP15, UTP25 |
| GO:0042273 | ribosomal large subunit biogenesis | 45 | 14 | 1.61 | 1.8e-10 | ALB1, BRX1, CIC1, MRT4, NOP15, PUF6, REI1, RLI1, RPF2, RRP14, RRP5, RRP8, SSF1, TIF6 |
| GO:0071840 | cellular component organization or biogenesis | 1424 | 79 | 51 | 3.6e-07 | ALB1, BMS1, BRX1, BUD23, CAC2, CAM1, CBF5, CGR1, CIC1, COX18, CTF4, DBP10, DBP3, DBP7, DHR2, DIP2, DNA2, DRS1, EFG1, ESF1, FUN12, HIR1, IMP4, KAP123, KLMA_50458, KLMA_60262, KRI1, LCP5, MAK5, MDH2, MDJ2, MRPL35, MRT4, NMD3, NOC2, NOC3, NOG2, NOP1, NOP15, NOP2, NOP4, NOP56, NOP7, NPR3, NSA1, NSA2, NSR1, PDS5, PNO1, POL12, PTP2, PUF6, PXR1, REI1, REX4, RFA1, RLF2, RLI1, RPF2, RPL8B, RRP14, RRP4, RRP42, RRP45, RRP5, RRP8, RSA3, RSM22, SAS10, SDO1, SKI6, SPB1, SPC105, SSF1, TIF6, TIM17, TSR2, UTP15, UTP25 |
| GO:0000466 | maturation of 5.8S rRNA from tricistronic rRNA transcript (SSU-rRNA, 5.8S rRNA, LSU-rRNA) | 46 | 11 | 1.65 | 3.7e-07 | BUD23, DIP2, KRI1, RPF2, RRP4, RRP42, RRP45, RRP5, SAS10, SKI6, TIF6 |
| GO:0042255 | ribosome assembly | 29 | 9 | 1.04 | 4.0e-07 | BMS1, BRX1, MRT4, NOC2, NSR1, RPF2, SDO1, SSF1, TIF6 |
| GO:0000460 | maturation of 5.8S rRNA | 47 | 11 | 1.68 | 4.7e-07 | BUD23, DIP2, KRI1, RPF2, RRP4, RRP42, RRP45, RRP5, SAS10, SKI6, TIF6 |
| GO:0016070 | RNA metabolic process | 923 | 58 | 33.06 | 1.0e-06 | BMS1, BUD23, BUD31, CAM1, CBF5, CGR1, DBP10, DBP3, DBP7, DHR2, DIP2, EFG1, ELP6, ESF1, FEN1, FUN12, HIR1, ILS1, IMP4, KLMA_20616, KRI1, LCP5, MAK5, MRT4, NOC3, NOP1, NOP15, NOP2, NOP4, NOP56, NOP7, NSA1, NSA2, NSR1, POL1, PUS4, PXR1, RBA50, REX4, RLI1, RPA34, RPC25, RPC37, RPF2, RRP14, RRP4, RRP42, RRP45, RRP5, SAS10, SKI6, SPB1, SPT6, TIF6, TRM82, TSR2, UTP15, UTP25 |
| GO:0090304 | nucleic acid metabolic process | 1152 | 66 | 41.26 | 3.4e-06 | BMS1, BUD23, BUD31, CAC2, CAM1, CBF5, CGR1, CTF4, DBP10, DBP3, DBP7, DHR2, DIP2, DNA2, EFG1, ELP6, ESF1, FEN1, FUN12, HIR1, ILS1, IMP4, KLMA_20616, KRI1, LCP5, MAK5, MRT4, NOC3, NOP1, NOP15, NOP2, NOP4, NOP56, NOP7, NSA1, NSA2, NSR1, PDS5, POL1, POL12, PUS4, PXR1, RBA50, REX4, RFA1, RLF2, RLI1, RNR1, RPA34, RPC25, RPC37, RPF2, RRP14, RRP4, RRP42, RRP45, RRP5, SAS10, SKI6, SPB1, SPT6, TIF6, TRM82, TSR2, UTP15, UTP25 |
| GO:0000469 | cleavage involved in rRNA processing | 39 | 9 | 1.4 | 6.3e-06 | BUD23, DIP2, KRI1, RRP4, RRP42, RRP45, RRP5, SAS10, SKI6 |
| GO:0006139 | nucleobase-containing compound metabolic process | 1330 | 72 | 47.63 | 7.3e-06 | BMS1, BUD23, BUD31, CAC2, CAM1, CBF5, CDC21, CGR1, CTF4, DAS2, DBP10, DBP3, DBP7, DCD1, DHR2, DIP2, DNA2, DUT1, EFG1, ELP6, ESF1, FEN1, FUN12, HIR1, ILS1, IMP4, KLMA_20616, KRI1, LCP5, MAK5, MEF2, MRT4, NOC3, NOP1, NOP15, NOP2, NOP4, NOP56, NOP7, NSA1, NSA2, NSR1, PDS5, POL1, POL12, PUS4, PXR1, RBA50, REX4, RFA1, RLF2, RLI1, RNR1, RPA34, RPC25, RPC37, RPF2, RRP14, RRP4, RRP42, RRP45, RRP5, SAS10, SKI6, SPB1, SPT6, TIF6, TRM82, TSR2, URK1, UTP15, UTP25 |
| GO:0090501 | RNA phosphodiester bond hydrolysis | 40 | 9 | 1.43 | 7.8e-06 | BUD23, DIP2, KRI1, RRP4, RRP42, RRP45, RRP5, SAS10, SKI6 |
| GO:0000462 | maturation of SSU-rRNA from tricistronic rRNA transcript (SSU-rRNA, 5.8S rRNA, LSU-rRNA) | 44 | 9 | 1.58 | 1.8e-05 | BUD23, DHR2, DIP2, FUN12, KRI1, RRP5, SAS10, TSR2, UTP15 |
| GO:0042274 | ribosomal small subunit biogenesis | 67 | 11 | 2.4 | 1.9e-05 | BUD23, DHR2, DIP2, FUN12, KRI1, NSR1, RRP14, RRP5, SAS10, TSR2, UTP15 |
| GO:0030490 | maturation of SSU-rRNA | 48 | 9 | 1.72 | 3.8e-05 | BUD23, DHR2, DIP2, FUN12, KRI1, RRP5, SAS10, TSR2, UTP15 |
| GO:0009157 | deoxyribonucleoside monophosphate biosynthetic process | 3 | 3 | 0.11 | 4.5e-05 | CDC21, DCD1, DUT1 |
| GO:0009162 | deoxyribonucleoside monophosphate metabolic process | 3 | 3 | 0.11 | 4.5e-05 | CDC21, DCD1, DUT1 |
| GO:0009176 | pyrimidine deoxyribonucleoside monophosphate metabolic process | 3 | 3 | 0.11 | 4.5e-05 | CDC21, DCD1, DUT1 |
| GO:0009177 | pyrimidine deoxyribonucleoside monophosphate biosynthetic process | 3 | 3 | 0.11 | 4.5e-05 | CDC21, DCD1, DUT1 |
| GO:0006725 | cellular aromatic compound metabolic process | 1395 | 72 | 49.96 | 4.9e-05 | BMS1, BUD23, BUD31, CAC2, CAM1, CBF5, CDC21, CGR1, CTF4, DAS2, DBP10, DBP3, DBP7, DCD1, DHR2, DIP2, DNA2, DUT1, EFG1, ELP6, ESF1, FEN1, FUN12, HIR1, ILS1, IMP4, KLMA_20616, KRI1, LCP5, MAK5, MEF2, MRT4, NOC3, NOP1, NOP15, NOP2, NOP4, NOP56, NOP7, NSA1, NSA2, NSR1, PDS5, POL1, POL12, PUS4, PXR1, RBA50, REX4, RFA1, RLF2, RLI1, RNR1, RPA34, RPC25, RPC37, RPF2, RRP14, RRP4, RRP42, RRP45, RRP5, SAS10, SKI6, SPB1, SPT6, TIF6, TRM82, TSR2, URK1, UTP15, UTP25 |
| GO:0009147 | pyrimidine nucleoside triphosphate metabolic process | 7 | 4 | 0.25 | 5.1e-05 | CDC21, DAS2, DUT1, URK1 |
| GO:0009129 | pyrimidine nucleoside monophosphate metabolic process | 13 | 5 | 0.47 | 5.6e-05 | CDC21, DAS2, DCD1, DUT1, URK1 |
| GO:0009130 | pyrimidine nucleoside monophosphate biosynthetic process | 13 | 5 | 0.47 | 5.6e-05 | CDC21, DAS2, DCD1, DUT1, URK1 |
| GO:0034641 | cellular nitrogen compound metabolic process | 1463 | 74 | 52.39 | 7.2e-05 | ASP1, BMS1, BUD23, BUD31, CAC2, CAM1, CBF5, CDC21, CGR1, CTF4, DAS2, DBP10, DBP3, DBP7, DCD1, DHR2, DIP2, DNA2, DUT1, EFG1, ELP6, ESF1, FEN1, FUN12, HIR1, ILS1, IMP4, KLMA_20616, KRI1, LCP5, MAK5, MEF2, MRT4, NOC3, NOP1, NOP15, NOP2, NOP4, NOP56, NOP7, NSA1, NSA2, NSR1, PDS5, POL1, POL12, PSD1, PUS4, PXR1, RBA50, REX4, RFA1, RLF2, RLI1, RNR1, RPA34, RPC25, RPC37, RPF2, RRP14, RRP4, RRP42, RRP45, RRP5, SAS10, SKI6, SPB1, SPT6, TIF6, TRM82, TSR2, URK1, UTP15, UTP25 |
| GO:0046483 | heterocycle metabolic process | 1414 | 72 | 50.64 | 8.2e-05 | BMS1, BUD23, BUD31, CAC2, CAM1, CBF5, CDC21, CGR1, CTF4, DAS2, DBP10, DBP3, DBP7, DCD1, DHR2, DIP2, DNA2, DUT1, EFG1, ELP6, ESF1, FEN1, FUN12, HIR1, ILS1, IMP4, KLMA_20616, KRI1, LCP5, MAK5, MEF2, MRT4, NOC3, NOP1, NOP15, NOP2, NOP4, NOP56, NOP7, NSA1, NSA2, NSR1, PDS5, POL1, POL12, PUS4, PXR1, RBA50, REX4, RFA1, RLF2, RLI1, RNR1, RPA34, RPC25, RPC37, RPF2, RRP14, RRP4, RRP42, RRP45, RRP5, SAS10, SKI6, SPB1, SPT6, TIF6, TRM82, TSR2, URK1, UTP15, UTP25 |
| GO:0009262 | deoxyribonucleotide metabolic process | 8 | 4 | 0.29 | 9.8e-05 | CDC21, DCD1, DUT1, RNR1 |
| GO:0009263 | deoxyribonucleotide biosynthetic process | 8 | 4 | 0.29 | 9.8e-05 | CDC21, DCD1, DUT1, RNR1 |
| GO:1901360 | organic cyclic compound metabolic process | 1454 | 73 | 52.07 | 0.00012 | BMS1, BUD23, BUD31, CAC2, CAM1, CBF5, CDC21, CGR1, CTF4, DAS2, DBP10, DBP3, DBP7, DCD1, DHR2, DIP2, DNA2, DUT1, EFG1, ELP6, ESF1, FEN1, FUN12, HIR1, ILS1, IMP4, KLMA_20616, KRI1, LCP5, MAK5, MEF2, MRT4, NCP1, NOC3, NOP1, NOP15, NOP2, NOP4, NOP56, NOP7, NSA1, NSA2, NSR1, PDS5, POL1, POL12, PUS4, PXR1, RBA50, REX4, RFA1, RLF2, RLI1, RNR1, RPA34, RPC25, RPC37, RPF2, RRP14, RRP4, RRP42, RRP45, RRP5, SAS10, SKI6, SPB1, SPT6, TIF6, TRM82, TSR2, URK1, UTP15, UTP25 |
| GO:0009219 | pyrimidine deoxyribonucleotide metabolic process | 4 | 3 | 0.14 | 0.00017 | CDC21, DCD1, DUT1 |
| GO:0009221 | pyrimidine deoxyribonucleotide biosynthetic process | 4 | 3 | 0.14 | 0.00017 | CDC21, DCD1, DUT1 |
| GO:0009265 | 2'-deoxyribonucleotide biosynthetic process | 4 | 3 | 0.14 | 0.00017 | CDC21, DCD1, DUT1 |
| GO:0009394 | 2'-deoxyribonucleotide metabolic process | 4 | 3 | 0.14 | 0.00017 | CDC21, DCD1, DUT1 |
| GO:0019692 | deoxyribose phosphate metabolic process | 4 | 3 | 0.14 | 0.00017 | CDC21, DCD1, DUT1 |
| GO:0046385 | deoxyribose phosphate biosynthetic process | 4 | 3 | 0.14 | 0.00017 | CDC21, DCD1, DUT1 |
| GO:0090305 | nucleic acid phosphodiester bond hydrolysis | 101 | 12 | 3.62 | 0.00021 | BUD23, DIP2, DNA2, FEN1, KRI1, REX4, RRP4, RRP42, RRP45, RRP5, SAS10, SKI6 |
| GO:0070925 | organelle assembly | 60 | 9 | 2.15 | 0.00023 | BMS1, BRX1, MRT4, NOC2, NSR1, RPF2, SDO1, SSF1, TIF6 |
| GO:0006220 | pyrimidine nucleotide metabolic process | 17 | 5 | 0.61 | 0.00024 | CDC21, DAS2, DCD1, DUT1, URK1 |
| GO:0006221 | pyrimidine nucleotide biosynthetic process | 17 | 5 | 0.61 | 0.00024 | CDC21, DAS2, DCD1, DUT1, URK1 |
| GO:0006807 | nitrogen compound metabolic process | 1621 | 78 | 58.05 | 0.00025 | ARG1, ASP1, BMS1, BUD23, BUD31, CAC2, CAM1, CBF5, CDC21, CGR1, CTF4, CYS3, DAS2, DBP10, DBP3, DBP7, DCD1, DHR2, DIP2, DNA2, DUT1, EFG1, ELP6, ESF1, FEN1, FUN12, HIR1, ILS1, IMP4, KLMA_20616, KRI1, LCP5, LYS21, MAK5, MEF2, MRT4, NIT3, NOC3, NOP1, NOP15, NOP2, NOP4, NOP56, NOP7, NSA1, NSA2, NSR1, PDS5, POL1, POL12, PSD1, PUS4, PXR1, RBA50, REX4, RFA1, RLF2, RLI1, RNR1, RPA34, RPC25, RPC37, RPF2, RRP14, RRP4, RRP42, RRP45, RRP5, SAS10, SKI6, SPB1, SPT6, TIF6, TRM82, TSR2, URK1, UTP15, UTP25 |
| GO:0071042 | nuclear polyadenylation-dependent mRNA catabolic process | 12 | 4 | 0.43 | 0.00062 | RRP4, RRP42, RRP45, SKI6 |
| GO:0071047 | polyadenylation-dependent mRNA catabolic process | 12 | 4 | 0.43 | 0.00062 | RRP4, RRP42, RRP45, SKI6 |
| GO:0009148 | pyrimidine nucleoside triphosphate biosynthetic process | 6 | 3 | 0.21 | 0.00083 | CDC21, DAS2, URK1 |
| GO:0016078 | tRNA catabolic process | 13 | 4 | 0.47 | 0.00087 | RRP4, RRP42, RRP45, SKI6 |
| GO:0070651 | nonfunctional rRNA decay | 13 | 4 | 0.47 | 0.00087 | RRP4, RRP42, RRP45, SKI6 |
| GO:0071038 | nuclear polyadenylation-dependent tRNA catabolic process | 13 | 4 | 0.47 | 0.00087 | RRP4, RRP42, RRP45, SKI6 |
| GO:0071051 | polyadenylation-dependent snoRNA 3'-end processing | 13 | 4 | 0.47 | 0.00087 | RRP4, RRP42, RRP45, SKI6 |
| GO:0010467 | gene expression | 1331 | 65 | 47.67 | 0.00106 | ATP25, BMS1, BUD23, BUD31, CAM1, CBF5, CGR1, CIC1, DBP10, DBP3, DBP7, DHR2, DIP2, EFG1, ELP6, ESF1, FUN12, HIR1, ILS1, IMP4, KRI1, LCP5, MAK5, MEF2, MRPL35, MRT4, NMA111, NOC3, NOP1, NOP15, NOP2, NOP4, NOP56, NOP7, NSA1, NSA2, NSR1, PUS4, PXR1, RBA50, REX4, RLI1, RPA34, RPC25, RPC37, RPF2, RPL28, RPL8B, RRP14, RRP4, RRP42, RRP45, RRP5, RSM22, SAS10, SKI6, SPB1, SPT6, SSM4, TAE1, TIF6, TRM82, TSR2, UTP15, UTP25 |
| GO:0000447 | endonucleolytic cleavage in ITS1 to separate SSU-rRNA from 5.8S rRNA and LSU-rRNA from tricistronic rRNA transcript (SSU-rRNA, 5.8S rRNA, LSU-rRNA) | 23 | 5 | 0.82 | 0.00109 | BUD23, DIP2, KRI1, RRP5, SAS10 |
| GO:0000478 | endonucleolytic cleavage involved in rRNA processing | 23 | 5 | 0.82 | 0.00109 | BUD23, DIP2, KRI1, RRP5, SAS10 |
| GO:0000479 | endonucleolytic cleavage of tricistronic rRNA transcript (SSU-rRNA, 5.8S rRNA, LSU-rRNA) | 23 | 5 | 0.82 | 0.00109 | BUD23, DIP2, KRI1, RRP5, SAS10 |
| GO:0043633 | polyadenylation-dependent RNA catabolic process | 14 | 4 | 0.5 | 0.00119 | RRP4, RRP42, RRP45, SKI6 |
| GO:0043634 | polyadenylation-dependent ncRNA catabolic process | 14 | 4 | 0.5 | 0.00119 | RRP4, RRP42, RRP45, SKI6 |
| GO:0070481 | nuclear-transcribed mRNA catabolic process, non-stop decay | 14 | 4 | 0.5 | 0.00119 | RRP4, RRP42, RRP45, SKI6 |
| GO:0071029 | nuclear ncRNA surveillance | 14 | 4 | 0.5 | 0.00119 | RRP4, RRP42, RRP45, SKI6 |
| GO:0071046 | nuclear polyadenylation-dependent ncRNA catabolic process | 14 | 4 | 0.5 | 0.00119 | RRP4, RRP42, RRP45, SKI6 |
| GO:0006226 | dUMP biosynthetic process | 2 | 2 | 0.07 | 0.00127 | DCD1, DUT1 |
| GO:0006231 | dTMP biosynthetic process | 2 | 2 | 0.07 | 0.00127 | CDC21, DCD1 |
| GO:0044211 | CTP salvage | 2 | 2 | 0.07 | 0.00127 | DAS2, URK1 |
| GO:0046073 | dTMP metabolic process | 2 | 2 | 0.07 | 0.00127 | CDC21, DCD1 |
| GO:0046078 | dUMP metabolic process | 2 | 2 | 0.07 | 0.00127 | DCD1, DUT1 |
| GO:0090502 | RNA phosphodiester bond hydrolysis, endonucleolytic | 24 | 5 | 0.86 | 0.00134 | BUD23, DIP2, KRI1, RRP5, SAS10 |
| GO:0000459 | exonucleolytic trimming involved in rRNA processing | 15 | 4 | 0.54 | 0.00158 | RRP4, RRP42, RRP45, SKI6 |
| GO:0000467 | exonucleolytic trimming to generate mature 3'-end of 5.8S rRNA from tricistronic rRNA transcript (SSU-rRNA, 5.8S rRNA, LSU-rRNA) | 15 | 4 | 0.54 | 0.00158 | RRP4, RRP42, RRP45, SKI6 |
| GO:0006273 | lagging strand elongation | 15 | 4 | 0.54 | 0.00158 | DNA2, FEN1, POL1, POL12 |
| GO:0031125 | rRNA 3'-end processing | 15 | 4 | 0.54 | 0.00158 | RRP4, RRP42, RRP45, SKI6 |
| GO:0090503 | RNA phosphodiester bond hydrolysis, exonucleolytic | 15 | 4 | 0.54 | 0.00158 | RRP4, RRP42, RRP45, SKI6 |
| GO:0022618 | ribonucleoprotein complex assembly | 79 | 9 | 2.83 | 0.00180 | BMS1, BRX1, MRT4, NOC2, NSR1, RPF2, SDO1, SSF1, TIF6 |
| GO:0072528 | pyrimidine-containing compound biosynthetic process | 26 | 5 | 0.93 | 0.00196 | CDC21, DAS2, DCD1, DUT1, URK1 |
| GO:0034427 | nuclear-transcribed mRNA catabolic process, exonucleolytic, 3'-5' | 16 | 4 | 0.57 | 0.00204 | RRP4, RRP42, RRP45, SKI6 |
| GO:0043928 | exonucleolytic nuclear-transcribed mRNA catabolic process involved in deadenylation-dependent decay | 16 | 4 | 0.57 | 0.00204 | RRP4, RRP42, RRP45, SKI6 |
| GO:0070478 | nuclear-transcribed mRNA catabolic process, 3'-5' exonucleolytic nonsense-mediated decay | 16 | 4 | 0.57 | 0.00204 | RRP4, RRP42, RRP45, SKI6 |
| GO:0071826 | ribonucleoprotein complex subunit organization | 82 | 9 | 2.94 | 0.00235 | BMS1, BRX1, MRT4, NOC2, NSR1, RPF2, SDO1, SSF1, TIF6 |
| GO:0009451 | RNA modification | 53 | 7 | 1.9 | 0.00251 | BUD23, CBF5, ELP6, LCP5, NOP56, PUS4, SPB1 |
| GO:0000291 | nuclear-transcribed mRNA catabolic process, exonucleolytic | 17 | 4 | 0.61 | 0.00260 | RRP4, RRP42, RRP45, SKI6 |
| GO:0034475 | U4 snRNA 3'-end processing | 9 | 3 | 0.32 | 0.00322 | RRP4, RRP45, SKI6 |
| GO:0006335 | DNA replication-dependent nucleosome assembly | 3 | 2 | 0.11 | 0.00373 | CAC2, RLF2 |
| GO:0009211 | pyrimidine deoxyribonucleoside triphosphate metabolic process | 3 | 2 | 0.11 | 0.00373 | CDC21, DUT1 |
| GO:0034723 | DNA replication-dependent nucleosome organization | 3 | 2 | 0.11 | 0.00373 | CAC2, RLF2 |
| GO:0000027 | ribosomal large subunit assembly | 19 | 4 | 0.68 | 0.00401 | BRX1, MRT4, RPF2, SSF1 |
| GO:0016075 | rRNA catabolic process | 19 | 4 | 0.68 | 0.00401 | RRP4, RRP42, RRP45, SKI6 |
| GO:0072527 | pyrimidine-containing compound metabolic process | 31 | 5 | 1.11 | 0.00438 | CDC21, DAS2, DCD1, DUT1, URK1 |
| GO:0000154 | rRNA modification | 10 | 3 | 0.36 | 0.00448 | BUD23, LCP5, NOP56 |
| GO:0071025 | RNA surveillance | 20 | 4 | 0.72 | 0.00487 | RRP4, RRP42, RRP45, SKI6 |
| GO:0071027 | nuclear RNA surveillance | 20 | 4 | 0.72 | 0.00487 | RRP4, RRP42, RRP45, SKI6 |
| GO:0034661 | ncRNA catabolic process | 21 | 4 | 0.75 | 0.00585 | RRP4, RRP42, RRP45, SKI6 |
| GO:0000463 | maturation of LSU-rRNA from tricistronic rRNA transcript (SSU-rRNA, 5.8S rRNA, LSU-rRNA) | 11 | 3 | 0.39 | 0.00600 | RPF2, RRP5, TIF6 |
| GO:0031126 | snoRNA 3'-end processing | 22 | 4 | 0.79 | 0.00696 | RRP4, RRP42, RRP45, SKI6 |
| GO:0006241 | CTP biosynthetic process | 4 | 2 | 0.14 | 0.00728 | DAS2, URK1 |
| GO:0009208 | pyrimidine ribonucleoside triphosphate metabolic process | 4 | 2 | 0.14 | 0.00728 | DAS2, URK1 |
| GO:0009209 | pyrimidine ribonucleoside triphosphate biosynthetic process | 4 | 2 | 0.14 | 0.00728 | DAS2, URK1 |
| GO:0010138 | pyrimidine ribonucleotide salvage | 4 | 2 | 0.14 | 0.00728 | DAS2, URK1 |
| GO:0032262 | pyrimidine nucleotide salvage | 4 | 2 | 0.14 | 0.00728 | DAS2, URK1 |
| GO:0034473 | U1 snRNA 3'-end processing | 4 | 2 | 0.14 | 0.00728 | RRP45, SKI6 |
| GO:0042256 | mature ribosome assembly | 4 | 2 | 0.14 | 0.00728 | SDO1, TIF6 |
| GO:0044206 | UMP salvage | 4 | 2 | 0.14 | 0.00728 | DAS2, URK1 |
| GO:0046036 | CTP metabolic process | 4 | 2 | 0.14 | 0.00728 | DAS2, URK1 |
| GO:0000054 | ribosomal subunit export from nucleus | 35 | 5 | 1.25 | 0.00746 | BUD23, NMD3, NOC2, RLI1, TIF6 |
| GO:0000288 | nuclear-transcribed mRNA catabolic process, deadenylation-dependent decay | 35 | 5 | 1.25 | 0.00746 | KLMA_20616, RRP4, RRP42, RRP45, SKI6 |
| GO:0033750 | ribosome localization | 35 | 5 | 1.25 | 0.00746 | BUD23, NMD3, NOC2, RLI1, TIF6 |
| GO:0033753 | establishment of ribosome localization | 35 | 5 | 1.25 | 0.00746 | BUD23, NMD3, NOC2, RLI1, TIF6 |
| GO:0071166 | ribonucleoprotein complex localization | 35 | 5 | 1.25 | 0.00746 | BUD23, NMD3, NOC2, RLI1, TIF6 |
| GO:0071426 | ribonucleoprotein complex export from nucleus | 35 | 5 | 1.25 | 0.00746 | BUD23, NMD3, NOC2, RLI1, TIF6 |
| GO:0071428 | rRNA-containing ribonucleoprotein complex export from nucleus | 35 | 5 | 1.25 | 0.00746 | BUD23, NMD3, NOC2, RLI1, TIF6 |
| GO:0000470 | maturation of LSU-rRNA | 12 | 3 | 0.43 | 0.00779 | RPF2, RRP5, TIF6 |
| GO:0034472 | snRNA 3'-end processing | 12 | 3 | 0.43 | 0.00779 | RRP4, RRP45, SKI6 |
| GO:0000184 | nuclear-transcribed mRNA catabolic process, nonsense-mediated decay | 23 | 4 | 0.82 | 0.00819 | RRP4, RRP42, RRP45, SKI6 |
| GO:0043170 | macromolecule metabolic process | 1877 | 81 | 67.22 | 0.00854 | ATP25, BMS1, BUD23, BUD31, CAC2, CAM1, CBF5, CGR1, CIC1, CTF4, DBP10, DBP3, DBP7, DHR2, DIP2, DNA2, DPH2, EFG1, ELP6, ESF1, FAS2, FEN1, FUN12, HIR1, HPM1, ILS1, IMP4, KLMA_20616, KRI1, LCP5, MAK5, MEF2, MRPL35, MRT4, NMA111, NOC3, NOP1, NOP15, NOP2, NOP4, NOP56, NOP7, NSA1, NSA2, NSR1, PDS5, PGU1, POL1, POL12, PTP2, PUS4, PXR1, RBA50, REX4, RFA1, RLF2, RLI1, RNR1, RPA34, RPC25, RPC37, RPF2, RPL28, RPL8B, RRP14, RRP4, RRP42, RRP45, RRP5, RSM22, SAS10, SKI6, SPB1, SPT6, SSM4, TAE1, TIF6, TRM82, TSR2, UTP15, UTP25 |
| GO:0016180 | snRNA processing | 13 | 3 | 0.47 | 0.00986 | RRP4, RRP45, SKI6 |

^a^The number of GO term annotated genes in the *K. marxianus* genome.

^b^The number of GO term annotated genes, which were significantly (FDR < 0.05) expressed under the condition.

^c^The expected value of Fisher's exact test.

^d^The P-value of Fisher's exact test.

**Table S10** Summary of significantly up-regulated genes under the 30DS condition

| Locus_tag | logFC | logCPM | FDR | Product | UniProt gene | KO number | Localization | Specific^a^ |
| --- | --- | --- | --- | --- | --- | --- | --- | --- |
| KLMA_80413 | 18.7 | 9.5 | 0.000 | 60S ribosomal protein L8-B | RPL8B | K02936 | Cytoplasm. | C |
| KLMA_10180 | 17.4 | 7.3 | 0.006 | argininosuccinate synthase | ARG1 | K01940 | Cytoplasm. | 30X |
| KLMA_10496 | 16.4 | 5.9 | 0.000 | rRNA-processing protein EFG1 | EFG1 |  | Nucleus, nucleolus (By similarity). | S |
| KLMA_70354 | 15.9 | 6.2 | 0.002 | ADP,ATP carrier protein | AAC | K05863 | Mitochondrion inner membrane; Multi-pass membrane protein. | 45D |
| KLMA_30371 | 15.5 | 5.8 | 0.030 | calcium/calmodulin-dependent protein kinase II | CMK2 | K00908 |  | 45D |
| KLMA_80051 | 15.3 | 4.8 | 0.002 | phosphoglucomutase YMR278W | PGM3 | K01835 | Cytoplasm. Nucleus. | S |
| KLMA_10353 | 14.4 | 3.9 | 0.000 | mitochondrial import inner membrane translocase subunit TIM17 | TIM17 |  | Mitochondrion inner membrane; Multi-pass membrane protein. | S |
| KLMA_10516 | 14.4 | 4.0 | 0.000 | polygalacturonase | PGU1 | K01184 |  | C |
| KLMA_60474 | 14.2 | 4.5 | 0.001 | putative methyltransferase BUD23 | BUD23 |  | Cytoplasm. Nucleus. | C |
| KLMA_10225 | 14.1 | 5.3 | 0.000 | GTP-binding nuclear protein GSP1/Ran | GSP1 | K07936 | Nucleus (By similarity). | 30X |
| KLMA_50396 | 14.0 | 3.6 | 0.005 | uncharacterized mitochondrial carrier YIL006W | YIA6 | K15115 | Mitochondrion inner membrane; Multi-pass membrane protein. | S |
| KLMA_50089 | 13.7 | 3.3 | 0.014 | methyltransferase-like protein YBR261C | TAE1 |  | Cytoplasm. | S |
| KLMA_40373 | 13.5 | 7.0 | 0.006 | hypothetical protein | OM45 |  | Mitochondrion outer membrane. | 30X |
| KLMA_20354 | 13.4 | 2.9 | 0.023 | cystathionine gamma-lyase | CYS3 | K01758 | Cytoplasm. | S |
| KLMA_20381 | 13.4 | 2.9 | 0.019 | hypothetical protein | OSW5 |  | Membrane; Multi-pass membrane protein (By similarity). | S |
| KLMA_10783 | 13.1 | 7.0 | 0.005 | sorbose reductase SOU1 | SOU1 |  |  | C |
| KLMA_60493 | 13.1 | 3.8 | 0.026 | homocitrate synthase | LYS21 | K01655 | Mitochondrion (Potential). | C |
| KLMA_10267 | 13.1 | 5.2 | 0.000 | SAP super family | AIM34 |  | Mitochondrion membrane; Single-pass membrane protein (By similarity). | 30X |
| KLMA_50281 | 13.1 | 2.6 | 0.017 | mitochondrial inner membrane protein COX18 | COX18 |  | Mitochondrion inner membrane; Multi-pass membrane protein. | S |
| KLMA_30641 | 13.0 | 6.2 | 0.013 | probable hydrolase NIT3 | NIT3 |  |  | C |
| KLMA_70421 | 12.9 | 2.4 | 0.047 | DNA-directed RNA polymerase III subunit RPC8 | RPC25 | K03022 | Nucleus. | S |
| KLMA_40101 | 12.8 | 2.4 | 0.002 | protein RMD11 | NPR3 |  |  | S |
| KLMA_50217 | 12.7 | 2.2 | 0.008 | serine hydrolase YJU3 | YJU3 |  | Cytoplasm. Endoplasmic reticulum. Lipid droplet. Mitochondrion outer membrane. | S |
| KLMA_30482 | 12.7 | 7.9 | 0.000 | glyoxalase super family protein |  |  |  | 30X |
| KLMA_40309 | 12.5 | 2.0 | 0.011 | endoplasmic reticulum transmembrane protein 1 | YET1 |  | Endoplasmic reticulum membrane; Multi-pass membrane protein. | S |
| KLMA_20616 | 11.9 | 4.4 | 0.019 | uncharacterized protein YDR370C |  |  | Cytoplasm. | 45D |
| KLMA_10226 | 11.9 | 1.4 | 0.022 | hypothetical protein |  |  |  | S |
| KLMA_R308 | 11.7 | 8.6 | 0.005 | Lys-tRNA |  |  |  | 45D |
| KLMA_30499 | 11.6 | 1.1 | 0.027 | bud site selection protein 31 | BUD31 | K12873 | Nucleus (Potential). | S |
| KLMA_20496 | 11.5 | 2.5 | 0.013 | hypothetical protein |  | K11098 |  | C |
| KLMA_80158 | 11.5 | 1.0 | 0.027 | mitotic exit network interactor 1 | HPM1 |  | Cytoplasm. Nucleus. | S |
| KLMA_30167 | 11.4 | 2.1 | 0.001 | hypothetical protein |  |  |  | C |
| KLMA_20730 | 11.3 | 0.8 | 0.042 | hypothetical protein |  |  |  | S |
| KLMA_40347 | 11.1 | 1.4 | 0.032 | protein SSM4 | SSM4 |  | Endoplasmic reticulum membrane; Multi-pass membrane protein. Nucleus inner membrane; Multi-pass membrane protein. | S |
| KLMA_10682 | 11.0 | 0.5 | 0.041 | thioredoxin reductase | TRR1 | K00384 | Mitochondrion (Potential). | S |
| KLMA_R416 | 10.6 | 4.5 | 0.000 | Val-tRNA |  |  |  | C |
| KLMA_R519 | 10.5 | 7.2 | 0.013 | Gly-tRNA |  |  |  | 45D |
| KLMA_80091 | 10.3 | 5.9 | 0.011 | exosome complex component RRP42 | RRP42 | K12589 | Cytoplasm. Nucleus, nucleolus. | 45D |
| KLMA_20617 | 10.1 | 0.9 | 0.007 | SAM50-like protein SpAC17C9.06 |  |  |  | 30X |
| KLMA_R127 | 10.0 | 4.9 | 0.010 | Val-tRNA |  |  |  | C |
| KLMA_R322 | 9.8 | 4.0 | 0.027 | Gln-tRNA |  |  |  | C |
| KLMA_60403 | 9.7 | 6.1 | 0.001 | putative elongation factor 1 gamma homolog | CAM1 | K03233 | Cytoplasm. Nucleus. | C |
| KLMA_R608 | 8.9 | 1.7 | 0.015 | Met-tRNA |  |  |  | 45D |
| KLMA_R602 | 8.5 | 2.8 | 0.017 | Ala-tRNA |  |  |  | C |
| KLMA_30511 | 8.0 | 4.1 | 0.002 | ATP-dependent RNA helicase DBP3 | DBP3 | K14811 | Nucleus, nucleolus (By similarity). | 30X |
| KLMA_70304 | 7.8 | 6.3 | 0.000 | transcription elongation factor SPT6 | SPT6 | K11292 | Nucleus (By similarity). | C |
| KLMA_60031 | 7.7 | 2.5 | 0.026 | tRNA (adenine-N(1)-)-methyltransferase catalytic subunit TRM61 | TRM61 | K07442 | Nucleus (By similarity). | S |
| KLMA_10038 | 7.7 | 2.8 | 0.006 | RNA polymerase II-associated protein RBA50 | RBA50 |  | Cytoplasm. | S |
| KLMA_70323 | 7.5 | 6.8 | 0.026 | uncharacterized protein YOL036W |  |  |  | S |
| KLMA_20051 | 7.2 | 4.3 | 0.000 | rRNA-processing protein CGR1 | CGR1 | K14822 | Nucleus, nucleolus (By similarity). | C |
| KLMA_50459 | 6.7 | 2.2 | 0.042 | deoxyuridine 5'-triphosphate nucleotidohydrolase | DUT1 | K01520 |  | S |
| KLMA_30139 | 6.7 | 5.9 | 0.027 | tRNA (cytosine-5-)-methyltransferase NCL1 | NCL1 | K15334 | Nucleus, nucleolus. | S |
| KLMA_50586 | 6.7 | 3.8 | 0.031 | serine/threonine-protein phosphatase 4 regulatory subunit 3 | PSY2 |  | Nucleus (By similarity). | S |
| KLMA_70414 | 6.6 | 8.3 | 0.007 | ribosome biogenesis protein NSA2 | NSA2 | K14842 | Nucleus, nucleolus (By similarity). | S |
| KLMA_20626 | 6.4 | 2.9 | 0.002 | mitochondrial DnaJ homolog 2 | MDJ2 |  | Mitochondrion inner membrane. | C |
| KLMA_60025 | 6.3 | 5.0 | 0.004 | ribosome biogenesis protein ALB1 | ALB1 | K14814 | Cytoplasm (By similarity). Nucleus (By similarity). | S |
| KLMA_60179 | 6.3 | 4.5 | 0.001 | probable ATP-dependent RNA helicase DHR2 | DHR2 | K14781 | Nucleus, nucleolus. | 45D |
| KLMA_70346 | 6.2 | 3.1 | 0.010 | DNA polymerase alpha subunit B | POL12 | K02321 | Nucleus. | 30X |
| KLMA_30593 | 6.2 | 5.1 | 0.001 | spindle pole component SPC72 |  |  |  | S |
| KLMA_60204 | 6.2 | 7.2 | 0.000 | ribosomal RNA-processing protein 8 | RRP8 | K14850 | Nucleus, nucleolus. Chromosome, telomere (Potential). | 30X |
| KLMA_80272 | 6.1 | 2.1 | 0.023 | pumilio homology domain family member 6 | PUF6 | K14844 | Bud tip. Nucleus, nucleolus. | S |
| KLMA_40391 | 6.1 | 6.9 | 0.000 | malate dehydrogenase | MDH2 | K00026 | Cytoplasm. | C |
| KLMA_70395 | 6.1 | 4.5 | 0.000 | tRNA pseudouridine synthase 4 | PUS4 | K03177 | Nucleus. Mitochondrion. | C |
| KLMA_70273 | 6.0 | 3.9 | 0.022 | WD repeat-containing protein JIP5 | JIP5 |  | Nucleus, nucleolus (By similarity). | S |
| KLMA_60172 | 6.0 | 7.2 | 0.000 | ribosomal RNA-processing protein 14 | RRP14 |  | Nucleus, nucleolus. | 30X |
| KLMA_60262 | 6.0 | 3.0 | 0.005 | UPF0195 protein YHR122W |  |  |  | 30X |
| KLMA_40375 | 5.9 | 4.3 | 0.004 | U3 small nucleolar ribonucleoprotein protein IMP4 | IMP4 | K14561 | Nucleus, nucleolus. | 45D |
| KLMA_30060 | 5.8 | 5.2 | 0.003 | mRNA turnover protein 4 | MRT4 | K14815 | Nucleus, nucleolus. | S |
| KLMA_70415 | 5.7 | 8.2 | 0.000 | U3 small nucleolar ribonucleoprotein protein LCP5 | LCP5 | K14765 | Nucleus, nucleolus. | 45D |
| KLMA_30567 | 5.7 | 2.8 | 0.005 | uncharacterized protein YMR098C | ATP25 |  | Mitochondrion inner membrane; Peripheral membrane protein; Matrix side (By similarity). | 45D |
| KLMA_10595 | 5.7 | 6.6 | 0.017 | eukaryotic translation initiation factor 6 | TIF6 | K03264 | Cytoplasm. Nucleus, nucleolus. | S |
| KLMA_70374 | 5.7 | 4.5 | 0.018 | tRNA (guanine-N(1)-)-methyltransferase | TRM5 | K15429 | Mitochondrion matrix. Nucleus. Cytoplasm. | S |
| KLMA_50152 | 5.5 | 2.5 | 0.025 | protein HIR1 | HIR1 | K11293 | Nucleus (By similarity). | S |
| KLMA_80380 | 5.5 | 5.3 | 0.000 | importin subunit beta-4 | KAP123 |  | Cytoplasm. Nucleus. Nucleus, nuclear pore complex. | S |
| KLMA_20725 | 5.4 | 5.6 | 0.007 | NADPH--cytochrome P450 reductase | NCP1 | K00327 | Endoplasmic reticulum membrane; Single-pass membrane protein. Mitochondrion outer membrane; Single-pass membrane protein. Cell membrane; Single-pass membrane protein. Microsome. | 45D |
| KLMA_70219 | 5.4 | 6.9 | 0.011 | fatty acid synthase subunit alpha | FAS2 | K00667 |  | S |
| KLMA_20345 | 5.2 | 9.4 | 0.008 | protein FUN14 |  |  |  | 30X |
| KLMA_80369 | 5.2 | 6.4 | 0.032 | isoleucyl-tRNA synthetase | ILS1 | K01870 | Cytoplasm. | S |
| KLMA_50229 | 5.2 | 5.6 | 0.039 | putative uridine kinase YDR020C | DAS2 |  | Cytoplasm. Nucleus. | 45D |
| KLMA_10031 | 5.0 | 4.2 | 0.001 | adoMet-dependent rRNA methyltransferase SPB1 | SPB1 | K14857 | Nucleus, nucleolus (By similarity). | 45D |
| KLMA_30382 | 5.0 | 7.7 | 0.000 | nuclear localization sequence-binding protein | NSR1 | K11294 | Nucleus. Nucleus, nucleolus (Potential). | S |
| KLMA_80194 | 5.0 | 3.2 | 0.039 | protein PXR1 | PXR1 | K11135 | Nucleus, nucleolus (By similarity). | S |
| KLMA_20128 | 4.9 | 8.1 | 0.032 | ribosome assembly protein 3 | RSA3 | K14854 | Nucleus, nucleolus (By similarity). | S |
| KLMA_10418 | 4.8 | 5.2 | 0.001 | ribosome biogenesis protein RPF2 | RPF2 | K14847 | Nucleus, nucleolus. | 45D |
| KLMA_50291 | 4.8 | 2.8 | 0.018 | rRNA biogenesis protein RRP5 | RRP5 | K14792 | Nucleus, nucleolus. | 45D |
| KLMA_70170 | 4.8 | 7.6 | 0.002 | ribonucleoside-diphosphate reductase large chain 1 | RNR1 | K10807 | Cytoplasm. | 30X |
| KLMA_60324 | 4.8 | 3.7 | 0.019 | sister chromatid cohesion protein PDS5 | PDS5 | K11267 | Nucleus. | S |
| KLMA_60349 | 4.7 | 4.5 | 0.003 | spindle pole body component SPC105 | SPC105 | K11563 | Cytoplasm, cytoskeleton, spindle pole body. Nucleus membrane; Peripheral membrane protein; Nucleoplasmic side. Chromosome, centromere, kinetochore. | C |
| KLMA_80234 | 4.7 | 5.1 | 0.014 | DNA polymerase alpha catalytic subunit A | POL1 | K02320 | Nucleus. | S |
| KLMA_50445 | 4.7 | 4.7 | 0.025 | pescadillo homolog | NOP7 | K14843 | Nucleus, nucleolus (By similarity). Nucleus, nucleoplasm (By similarity). | S |
| KLMA_60396 | 4.6 | 5.8 | 0.001 | nucleolar protein 4 | NOP4 | K14573 | Nucleus, nucleolus. | S |
| KLMA_20139 | 4.5 | 5.0 | 0.037 | ATP-dependent RNA helicase DBP7 | DBP7 | K14806 | Nucleus, nucleolus (By similarity). | S |
| KLMA_30565 | 4.4 | 4.4 | 0.004 | structure-specific endonuclease RAD27 | FEN1 | K04799 | Nucleus, nucleolus (By similarity). Nucleus, nucleoplasm (By similarity). Mitochondrion (By similarity). | S |
| KLMA_70291 | 4.4 | 2.5 | 0.018 | DNA polymerase alpha-binding protein | CTF4 | K11274 | Nucleus. | S |
| KLMA_70238 | 4.4 | 7.5 | 0.002 | something about silencing protein 10 | SAS10 | K14767 | Nucleus, nucleolus. | S |
| KLMA_60250 | 4.4 | 4.8 | 0.048 | pro-apoptotic serine protease NMA111 | NMA111 |  | Nucleus (By similarity). | S |
| KLMA_20683 | 4.3 | 4.1 | 0.007 | nucleolar complex-associated protein 3 | NOC3 | K14834 | Nucleus, nucleolus. | S |
| KLMA_50072 | 4.3 | 4.1 | 0.050 | pre-60S factor REI1 | REI1 | K14816 | Cytoplasm. | S |
| KLMA_20233 | 4.3 | 5.4 | 0.009 | hypothetical protein | NSA1 | K14841 | Nucleus, nucleolus (By similarity). | S |
| KLMA_10597 | 4.2 | 2.5 | 0.026 | chromatin assembly factor 1 subunit p90 | RLF2 |  | Nucleus. | S |
| KLMA_50045 | 4.2 | 7.2 | 0.002 | nucleolar complex protein 2 | NOC2 | K14833 | Nucleus, nucleolus. | S |
| KLMA_50181 | 4.2 | 6.5 | 0.005 | pre-rRNA-processing protein TSR2 | TSR2 | K14800 | Cytoplasm. Nucleus. | S |
| KLMA_40173 | 4.2 | 10.7 | 0.000 | peroxiredoxin type-2 | AHP1 | K14171 | Cytoplasm. | 30X |
| KLMA_50201 | 4.1 | 4.8 | 0.026 | ribonucleases P/MRP protein subunit POP6 |  | K14524 |  | C |
| KLMA_40397 | 4.1 | 7.5 | 0.004 | protein KRI1 | KRI1 | K14786 | Nucleus, nucleolus. | S |
| KLMA_30592 | 4.0 | 5.2 | 0.036 | eukaryotic translation initiation factor 3 subunit B | PRT1 | K03253 | Cytoplasm (By similarity). | S |
| KLMA_10634 | 4.0 | 5.4 | 0.010 | diphthamide biosynthesis protein 2 | DPH2 |  | Cytoplasm (By similarity). | S |
| KLMA_40544 | 4.0 | 4.6 | 0.006 | ATP-dependent RNA helicase MRH4 | MRH4 |  | Mitochondrion (By similarity). | S |
| KLMA_50458 | 3.9 | 6.8 | 0.006 | uncharacterized protein YGR111W |  |  |  | 45D |
| KLMA_20136 | 3.9 | 3.6 | 0.017 | ribosome-releasing factor 2 | MEF2 | K02355 | Mitochondrion (By similarity). | S |
| KLMA_10082 | 3.9 | 5.1 | 0.007 | protein PLM2 | PLM2 |  | Nucleus (Probable). | C |
| KLMA_80170 | 3.8 | 5.3 | 0.011 | putative ribosomal RNA methyltransferase Nop2 | NOP2 | K14835 | Nucleus, nucleolus. | S |
| KLMA_60277 | 3.8 | 5.2 | 0.008 | ATP-dependent RNA helicase DBP10 | DBP10 | K14808 | Nucleus, nucleolus (By similarity). | 45D |
| KLMA_20751 | 3.7 | 5.5 | 0.013 | phosphatidylserine decarboxylase proenzyme 1 | PSD1 | K01613 | Mitochondrion inner membrane. | 45D |
| KLMA_10282 | 3.7 | 3.3 | 0.041 | pre-rRNA-processing protein PNO1 | PNO1 | K11884 | Cytoplasm. Nucleus, nucleolus (By similarity). | S |
| KLMA_60232 | 3.7 | 9.4 | 0.004 | ribosome biogenesis protein 15 | NOP15 | K14838 | Cytoplasm. Nucleus, nucleolus. | S |
| KLMA_10451 | 3.7 | 7.0 | 0.010 | replication factor A protein 1 | RFA1 | K07466 | Nucleus. | S |
| KLMA_10328 | 3.6 | 5.7 | 0.020 | DNA replication ATP-dependent helicase DNA2 | DNA2 | K10742 | Nucleus. Chromosome. | 45D |
| KLMA_30223 | 3.6 | 5.6 | 0.014 | phosphatase PSR1 | PSR1 | K15731 | Cell membrane. | S |
| KLMA_30301 | 3.6 | 5.0 | 0.010 | translation initiation factor RLI1 | RLI1 | K06174 | Cytoplasm. Nucleus. | S |
| KLMA_10509 | 3.6 | 6.5 | 0.031 | elongator complex protein 6 | ELP6 | K11377 | Cytoplasm. Nucleus. | S |
| KLMA_10414 | 3.6 | 3.8 | 0.032 | thymidylate synthase | CDC21 | K00560 | Nucleus. | 45D |
| KLMA_10361 | 3.6 | 8.9 | 0.005 | DNA-directed RNA polymerase I subunit RPA34 | RPA34 | K03003 | Nucleus, nucleolus. | S |
| KLMA_30400 | 3.6 | 5.6 | 0.014 | exosome complex component RRP45 | RRP45 | K03678 | Cytoplasm. Nucleus, nucleolus. | S |
| KLMA_30432 | 3.5 | 5.9 | 0.012 | protein BFR2 | BFR2 | K14782 | Nucleus, nucleolus (By similarity). | S |
| KLMA_50363 | 3.5 | 5.9 | 0.015 | low-affinity glucose transporter | RAG1 | K08139 | Membrane; Multi-pass membrane protein. | S |
| KLMA_20092 | 3.5 | 4.0 | 0.048 | 37S ribosomal protein S22 | RSM22 |  | Mitochondrion. | S |
| KLMA_20752 | 3.5 | 6.5 | 0.037 | deoxycytidylate deaminase | DCD1 | K01493 |  | 45D |
| KLMA_60210 | 3.5 | 6.4 | 0.019 | eukaryotic translation initiation factor 5B | FUN12 | K03243 |  | S |
| KLMA_20478 | 3.5 | 5.8 | 0.026 | centromere/microtubule-binding protein CBF5 | CBF5 | K11131 | Nucleus, nucleolus (By similarity). Chromosome, centromere (By similarity). Cytoplasm, cytoskeleton (By similarity). | S |
| KLMA_40079 | 3.4 | 10.7 | 0.000 | uncharacterized endoplamic reticulum membrane |  |  |  | 30X |
| KLMA_50520 | 3.4 | 5.5 | 0.039 | tRNA (guanine-N(7)-)-methyltransferase subunit TRM82 | TRM82 | K15443 | Nucleus (By similarity). | S |
| KLMA_30219 | 3.4 | 5.5 | 0.033 | ribosome maturation protein SDO1 | SDO1 | K14574 | Cytoplasm. Nucleus. | S |
| KLMA_30229 | 3.4 | 4.3 | 0.026 | ATP-dependent RNA helicase DRS1 | DRS1 | K13181 | Nucleus, nucleolus (By similarity). | S |
| KLMA_70394 | 3.4 | 3.6 | 0.036 | paxillin-like protein 1 | PXL1 |  |  | S |
| KLMA_40555 | 3.4 | 5.2 | 0.020 | ribosome biogenesis protein BMS1 | BMS1 | K14569 | Cytoplasm. Nucleus, nucleolus. | S |
| KLMA_30549 | 3.4 | 3.7 | 0.041 | ribosome biogenesis protein SSF2 | SSF1 | K14859 | Nucleus, nucleolus. | S |
| KLMA_40251 | 3.3 | 5.5 | 0.019 | proteasome-interacting protein CIC1 | CIC1 | K14783 | Nucleus, nucleolus. | S |
| KLMA_20688 | 3.3 | 5.1 | 0.026 | exosome complex component SKI6 | SKI6 | K11600 | Cytoplasm. Nucleus, nucleolus. | S |
| KLMA_40432 | 3.3 | 5.1 | 0.028 | 60S ribosomal export protein NMD3 | NMD3 | K07562 | Cytoplasm. Nucleus, nucleoplasm. | 45D |
| KLMA_20327 | 3.3 | 3.7 | 0.047 | uncharacterized protein YOR305W | RRG7 |  | Mitochondrion (By similarity). | S |
| KLMA_30393 | 3.3 | 5.0 | 0.027 | ATP-dependent RNA helicase MAK5 | MAK5 | K14805 | Nucleus, nucleolus (By similarity). | S |
| KLMA_80399 | 3.3 | 6.6 | 0.027 | pre-rRNA-processing protein ESF1 | ESF1 |  | Nucleus, nucleolus. | S |
| KLMA_60140 | 3.2 | 5.8 | 0.028 | RNA exonuclease 4 | REX4 | K01175 | Nucleus (By similarity). | S |
| KLMA_80126 | 3.2 | 8.1 | 0.017 | uncharacterized protein YIL091C | UTP25 | K14774 | Nucleus, nucleolus (By similarity). | 45D |
| KLMA_60136 | 3.2 | 4.3 | 0.046 | ribosome biogenesis protein BRX1 | BRX1 | K14820 | Nucleus, nucleolus. | 45D |
| KLMA_50487 | 3.1 | 5.2 | 0.030 | U3 small nucleolar RNA-associated protein 12 | DIP2 | K14556 | Nucleus, nucleolus. | S |
| KLMA_50345 | 3.1 | 8.3 | 0.042 | L-asparaginase 1 | ASP1 | K01424 | Cytoplasm. | S |
| KLMA_40012 | 3.1 | 6.9 | 0.043 | nucleolar GTP-binding protein 2 | NOG2 | K14537 | Nucleus, nucleolus (By similarity). | S |
| KLMA_20147 | 3.0 | 4.6 | 0.049 | U3 small nucleolar RNA-associated protein 15 | UTP15 | K14549 | Nucleus, nucleolus. | S |
| KLMA_70373 | 3.0 | 7.0 | 0.039 | exosome complex component RRP4 | RRP4 | K03679 | Cytoplasm. Nucleus, nucleolus. | S |
| KLMA_10199 | 2.9 | 7.8 | 0.034 | rRNA 2'-O-methyltransferase fibrillarin | NOP1 | K14563 | Nucleus, nucleolus (By similarity). | S |
| KLMA_20138 | 2.9 | 5.2 | 0.046 | DNA-directed RNA polymerase III subunit rpc5 | RPC37 | K14721 | Nucleus. | S |
| KLMA_60464 | 2.9 | 5.2 | 0.042 | uridine kinase | URK1 | K00876 | Cytoplasm. Nucleus. | S |
| KLMA_20067 | 2.9 | 7.6 | 0.042 | chromatin assembly factor 1 subunit p60 | CAC2 | K10751 | Nucleus. | S |
| KLMA_50311 | 2.9 | 9.0 | 0.033 | nucleolar protein 56 | NOP56 | K14564 | Nucleus, nucleolus. | S |
| KLMA_20813 | 2.7 | 8.9 | 0.042 | tyrosine-protein phosphatase 2 | PTP2 | K01104 | Cytoplasm. Nucleus. | C |
| KLMA_50350 | 2.5 | 10.5 | 0.026 | 54S ribosomal protein L35 | MRPL35 |  | Mitochondrion. | S |
| KLMA_20403 | 2.4 | 12.5 | 0.003 | ribosomal_L18e super family | RPL28 | K02900 | Cytoplasm (By similarity). | S |

^a^Gene expression was significantly (FDR < 0.05) altered under the following conditions: S, 30DS-specific up-regulation; C, commonly up-regulated under 30DS, 45D and 30X conditions; 45D, up-regulated under 45D and 30DS conditions; 30X, up-regulated under 30X and 30DS conditions.

**Table S11** GO terms enriched in significantly down-regulated genes under 30DS condition

| GO.ID | Term | Annotated  gene^a^ | Significant^b^ | Expected^c^ | P-value^d^ | Genes |
| --- | --- | --- | --- | --- | --- | --- |
| GO:0030437 | ascospore formation | 50 | 9 | 1.38 | 6.2e-06 | CPR1, DIT1, FKS3, FMP45, GLC7, KLMA_50101, OSW2, SPS19, UBI4 |
| GO:0034293 | sexual sporulation | 51 | 9 | 1.4 | 7.4e-06 | CPR1, DIT1, FKS3, FMP45, GLC7, KLMA_50101, OSW2, SPS19, UBI4 |
| GO:0043935 | sexual sporulation resulting in formation of a cellular spore | 51 | 9 | 1.4 | 7.4e-06 | CPR1, DIT1, FKS3, FMP45, GLC7, KLMA_50101, OSW2, SPS19, UBI4 |
| GO:0048468 | cell development | 51 | 9 | 1.4 | 7.4e-06 | CPR1, DIT1, FKS3, FMP45, GLC7, KLMA_50101, OSW2, SPS19, UBI4 |
| GO:0022413 | reproductive process in single-celled organism | 75 | 10 | 2.07 | 3.0e-05 | CPR1, DIT1, DPB11, FKS3, FMP45, GLC7, KLMA_50101, OSW2, SPS19, UBI4 |
| GO:0003006 | developmental process involved in reproduction | 77 | 10 | 2.12 | 3.8e-05 | CPR1, DIT1, DPB11, FKS3, FMP45, GLC7, KLMA_50101, OSW2, SPS19, UBI4 |
| GO:0044702 | single organism reproductive process | 78 | 10 | 2.15 | 4.2e-05 | CPR1, DIT1, DPB11, FKS3, FMP45, GLC7, KLMA_50101, OSW2, SPS19, UBI4 |
| GO:0019953 | sexual reproduction | 107 | 11 | 2.95 | 0.00014 | CPR1, DIT1, FAR1, FKS3, FMP45, GLC7, KLMA_50101, OSW2, RVS161, SPS19, UBI4 |
| GO:0044703 | multi-organism reproductive process | 107 | 11 | 2.95 | 0.00014 | CPR1, DIT1, FAR1, FKS3, FMP45, GLC7, KLMA_50101, OSW2, RVS161, SPS19, UBI4 |
| GO:0022414 | reproductive process | 127 | 12 | 3.5 | 0.00015 | CPR1, DIT1, DPB11, FAR1, FKS3, FMP45, GLC7, KLMA_50101, OSW2, RVS161, SPS19, UBI4 |
| GO:0009081 | branched-chain amino acid metabolic process | 20 | 5 | 0.55 | 0.00016 | ARO10, BAT1, ILV6, LEU4, MMF1 |
| GO:0051704 | multi-organism process | 140 | 12 | 3.86 | 0.00038 | CPR1, DIT1, FAR1, FKS3, FMP45, GLC7, KLMA_50101, MUC1, OSW2, RVS161, SPS19, UBI4 |
| GO:0009082 | branched-chain amino acid biosynthetic process | 14 | 4 | 0.39 | 0.00044 | BAT1, ILV6, LEU4, MMF1 |
| GO:0016054 | organic acid catabolic process | 68 | 8 | 1.87 | 0.00047 | ARO10, BAT1, BNA3, CIT3, KLMA_60382, PDH1, POT1, SPS19 |
| GO:0046395 | carboxylic acid catabolic process | 68 | 8 | 1.87 | 0.00047 | ARO10, BAT1, BNA3, CIT3, KLMA_60382, PDH1, POT1, SPS19 |
| GO:0030435 | sporulation resulting in formation of a cellular spore | 86 | 9 | 2.37 | 0.00050 | CPR1, DIT1, FKS3, FMP45, GLC7, KLMA_50101, OSW2, SPS19, UBI4 |
| GO:0048610 | cellular process involved in reproduction | 233 | 16 | 6.42 | 0.00051 | CDC15, CPR1, DIT1, DPB11, FAR1, FKS3, FMP45, GAC1, GLC7, KLMA_50101, OSW2, REC8, RMI1, RVS161, SPS19, UBI4 |
| GO:0008643 | carbohydrate transport | 26 | 5 | 0.72 | 0.00060 | HGT1, LAC12, MTH1 |
| GO:0043934 | sporulation | 89 | 9 | 2.45 | 0.00065 | CPR1, DIT1, FKS3, FMP45, GLC7, KLMA_50101, OSW2, SPS19, UBI4 |
| GO:0030154 | cell differentiation | 108 | 10 | 2.98 | 0.00066 | CPR1, DIT1, DPB11, FKS3, FMP45, GLC7, KLMA_50101, OSW2, SPS19, UBI4 |
| GO:0048646 | anatomical structure formation involved in morphogenesis | 90 | 9 | 2.48 | 0.00071 | CPR1, DIT1, FKS3, FMP45, GLC7, KLMA_50101, OSW2, SPS19, UBI4 |
| GO:0032505 | reproduction of a single-celled organism | 109 | 10 | 3 | 0.00071 | CPR1, DIT1, DPB11, FKS3, FMP45, GLC7, KLMA_50101, OSW2, SPS19, UBI4 |
| GO:0005975 | carbohydrate metabolic process | 247 | 16 | 6.8 | 0.00098 | ARDH, CIT3, DLD1, DOG2, FKS3, GAC1, GAL1, GAP1, GLC7, INU1, KLMA_60382, MDH1, PCK1, RAG5, SIP4, SOR1 |
| GO:0072329 | monocarboxylic acid catabolic process | 29 | 5 | 0.8 | 0.00101 | CIT3, KLMA_60382, PDH1, POT1, SPS19 |
| GO:0006551 | leucine metabolic process | 8 | 3 | 0.22 | 0.00103 | ARO10, BAT1, LEU4 |
| GO:0009097 | isoleucine biosynthetic process | 9 | 3 | 0.25 | 0.00151 | BAT1, ILV6, MMF1 |
| GO:0044262 | cellular carbohydrate metabolic process | 100 | 9 | 2.75 | 0.00151 | ARDH, CIT3, DLD1, FKS3, GAC1, GLC7, KLMA_60382, MDH1, SIP4 |
| GO:0000003 | reproduction | 265 | 16 | 7.3 | 0.00207 | CDC15, CPR1, DIT1, DPB11, FAR1, FKS3, FMP45, GAC1, GLC7, KLMA_50101, OSW2, REC8, RMI1, RVS161, SPS19, UBI4 |
| GO:0048869 | cellular developmental process | 126 | 10 | 3.47 | 0.00217 | CPR1, DIT1, DPB11, FKS3, FMP45, GLC7, KLMA_50101, OSW2, SPS19, UBI4 |
| GO:0019541 | propionate metabolic process | 3 | 2 | 0.08 | 0.00221 | CIT3, PDH1 |
| GO:0019543 | propionate catabolic process | 3 | 2 | 0.08 | 0.00221 | CIT3, PDH1 |
| GO:0019626 | short-chain fatty acid catabolic process | 3 | 2 | 0.08 | 0.00221 | CIT3, PDH1 |
| GO:0019629 | propionate catabolic process, 2-methylcitrate cycle | 3 | 2 | 0.08 | 0.00221 | CIT3, PDH1 |
| GO:0009062 | fatty acid catabolic process | 21 | 4 | 0.58 | 0.00226 | CIT3, PDH1, POT1, SPS19 |
| GO:0019752 | carboxylic acid metabolic process | 345 | 19 | 9.5 | 0.00226 | ARO10, ARO3, ARO8, BAT1, BIO3, BIO4, BNA3, CIT3, ILV6, KLMA_60382, LEU4, LYS12, MDH1, MMF1, PDH1, PDX3, POT1, SPS19, STR3 |
| GO:0009653 | anatomical structure morphogenesis | 106 | 9 | 2.92 | 0.00228 | CPR1, DIT1, FKS3, FMP45, GLC7, KLMA_50101, OSW2, SPS19, UBI4 |
| GO:0048856 | anatomical structure development | 107 | 9 | 2.95 | 0.00243 | CPR1, DIT1, FKS3, FMP45, GLC7, KLMA_50101, OSW2, SPS19, UBI4 |
| GO:0044282 | small molecule catabolic process | 89 | 8 | 2.45 | 0.00280 | ARO10, BAT1, BNA3, CIT3, KLMA_60382, PDH1, POT1, SPS19 |
| GO:0006549 | isoleucine metabolic process | 11 | 3 | 0.3 | 0.00285 | BAT1, ILV6, MMF1 |
| GO:0043436 | oxoacid metabolic process | 354 | 19 | 9.75 | 0.00305 | ARO10, ARO3, ARO8, BAT1, BIO3, BIO4, BNA3, CIT3, ILV6, KLMA_60382, LEU4, LYS12, MDH1, MMF1, PDH1, PDX3, POT1, SPS19, STR3 |
| GO:0032787 | monocarboxylic acid metabolic process | 111 | 9 | 3.06 | 0.00313 | BIO3, BIO4, BNA3, CIT3, KLMA_60382, PDH1, PDX3, POT1, SPS19 |
| GO:0006082 | organic acid metabolic process | 356 | 19 | 9.81 | 0.00325 | ARO10, ARO3, ARO8, BAT1, BIO3, BIO4, BNA3, CIT3, ILV6, KLMA_60382, LEU4, LYS12, MDH1, MMF1, PDH1, PDX3, POT1, SPS19, STR3 |
| GO:0071265 | L-methionine biosynthetic process | 12 | 3 | 0.33 | 0.00372 | ARO8, BAT1, STR3 |
| GO:0019318 | hexose metabolic process | 74 | 7 | 2.04 | 0.00387 | DOG2, GAL1, GAP1, PCK1, RAG5, SIP4, SOR1 |
| GO:0000076 | DNA replication checkpoint | 4 | 2 | 0.11 | 0.00435 | DPB11, GLC7 |
| GO:0046459 | short-chain fatty acid metabolic process | 4 | 2 | 0.11 | 0.00435 | CIT3, PDH1 |
| GO:0006790 | sulfur compound metabolic process | 97 | 8 | 2.67 | 0.00479 | ARO10, ARO8, BAT1, BIO3, BIO4, OPT1, PHO3, STR3 |
| GO:0044283 | small molecule biosynthetic process | 263 | 15 | 7.25 | 0.00496 | ARO10, ARO3, ARO8, BAT1, BIO3, BIO4, BNA3, ILV6, LEU4, LYS12, MMF1, PDX3, RIB3, STR3, UPC2 |
| GO:0005996 | monosaccharide metabolic process | 81 | 7 | 2.23 | 0.00639 | DOG2, GAL1, GAP1, PCK1, RAG5, SIP4, SOR1 |
| GO:0044767 | single-organism developmental process | 171 | 11 | 4.71 | 0.00673 | CPR1, DIT1, DPB11, FKS3, FMP45, GLC7, KLMA_50101, MDH1, OSW2, SPS19, UBI4 |
| GO:0009099 | valine biosynthetic process | 5 | 2 | 0.14 | 0.00712 | BAT1, ILV6 |
| GO:0006091 | generation of precursor metabolites and energy | 149 | 10 | 4.1 | 0.00726 | CIT3, COX5A, DLD1, FRE1, GAC1, GAP1, GLC7, MDH1, RAG5, RIB3 |
| GO:0032502 | developmental process | 174 | 11 | 4.79 | 0.00765 | CPR1, DIT1, DPB11, FKS3, FMP45, GLC7, KLMA_50101, MDH1, OSW2, SPS19, UBI4 |
| GO:0007093 | mitotic cell cycle checkpoint | 30 | 4 | 0.83 | 0.00857 | BAT1, DPB11, GAC1, GLC7 |
| GO:1901988 | negative regulation of cell cycle phase transition | 30 | 4 | 0.83 | 0.00857 | BAT1, DPB11, GAC1, GLC7 |
| GO:1901991 | negative regulation of mitotic cell cycle phase transition | 30 | 4 | 0.83 | 0.00857 | BAT1, DPB11, GAC1, GLC7 |
| GO:0044723 | single-organism carbohydrate metabolic process | 202 | 12 | 5.56 | 0.00874 | ARDH, DOG2, FKS3, GAC1, GAL1, GAP1, GLC7, KLMA_60382, PCK1, RAG5, SIP4, SOR1 |
| GO:0006766 | vitamin metabolic process | 48 | 5 | 1.32 | 0.00961 | BIO3, BIO4, PDX3, PHO3, RIB3 |
| GO:0006767 | water-soluble vitamin metabolic process | 48 | 5 | 1.32 | 0.00961 | BIO3, BIO4, PDX3, PHO3, RIB3 |

^a^The number of GO term annotated genes in the *K. marxianus* genome.

^b^The number of GO term annotated genes, which were significantly (FDR < 0.05) expressed under the condition.

^c^The expected value of Fisher's exact test.

^d^The P-value of Fisher's exact test.

**Table S12** Summary of significantly down-regulated genes under the 30DS conditions

| Locus_tag | logFC | logCPM | FDR | Product | UniProt gene | KO number | Localization | Specific^a^ |
| --- | --- | --- | --- | --- | --- | --- | --- | --- |
| KLMA_80303 | -17.4 | 7.1 | 0.017 | protein OPY2 |  |  |  | S |
| KLMA_40392 | -15.9 | 5.8 | 0.000 | uncharacterized vacuolar membrane protein YNL305C | BXI1 | K06890 | Endoplasmic reticulum membrane; Multi-pass membrane protein. Vacuole membrane; Multi-pass membrane protein. Mitochondrion membrane; Multi-pass membrane protein. | 30X |
| KLMA_20536 | -15.3 | 4.8 | 0.000 | yjgF_YER057c_UK114_family |  |  |  | C |
| KLMA_80089 | -15.1 | 6.3 | 0.002 | translation machinery-associated protein 17 | TMA17 |  | Cytoplasm. Nucleus. | 45D |
| KLMA_50500 | -14.6 | 4.1 | 0.006 | epsin-like protein | ENT5 |  | Cytoplasm. Endosome membrane; Peripheral membrane protein. | C |
| KLMA_50088 | -14.4 | 5.0 | 0.000 | uncharacterized protein YBR262C | AIM5 |  | Mitochondrion inner membrane; Single-pass membrane protein (By similarity). | 45D |
| KLMA_10139 | -14.3 | 4.9 | 0.002 | pre-mRNA-splicing factor CWC25 | CWC25 |  | Nucleus (By similarity). | 45D |
| KLMA_80058 | -13.4 | 5.7 | 0.007 | UPF0103 protein YJR008W |  | K06990 |  | S |
| KLMA_10732 | -13.0 | 3.3 | 0.049 | mitochondrial import inner membrane translocase subunit TIM9 | TIM9 |  | Mitochondrion inner membrane; Peripheral membrane protein; Intermembrane side (By similarity). | 45D |
| KLMA_20820 | -12.9 | 2.4 | 0.004 | hypothetical protein |  |  |  | 45D |
| KLMA_40601 | -12.2 | 2.0 | 0.016 | UBX domain-containing protein 2 | UBX2 | K14013 | Endoplasmic reticulum membrane; Multi-pass membrane protein (Potential). | S |
| KLMA_50270 | -11.8 | 2.4 | 0.024 | 3,4-dihydroxy-2-butanone 4-phosphate synthase | RIB3 | K02858 |  | S |
| KLMA_50256 | -11.8 | 1.3 | 0.007 | cyclin-dependent kinase inhibitor FAR1 | FAR1 | K06652 |  | 45D |
| KLMA_50406 | -11.8 | 5.4 | 0.025 | peroxiredoxin DOT5 | DOT5 | K03564 | Nucleus. Chromosome, telomere (Potential). | 45D |
| KLMA_20344 | -11.6 | 1.1 | 0.025 | mRNA 3'-end-processing protein YTH1 | YTH1 | K14404 | Nucleus (By similarity). | 45D |
| KLMA_40301 | -11.6 | 1.0 | 0.004 | UPF0067 GAF domain-containing protein YKL069W |  | K08968 | Cytoplasm. Nucleus. | C |
| KLMA_70331 | -11.5 | 2.0 | 0.012 | mitochondrial import inner membrane translocase subunit TIM12 | TIM12 |  | Mitochondrion inner membrane; Peripheral membrane protein. | 45D |
| KLMA_30346 | -11.1 | 4.9 | 0.011 | pre-mRNA-splicing factor CWC21 | CWC21 |  | Cytoplasm (By similarity). Nucleus (By similarity). | S |
| KLMA_60382 | -10.7 | 1.3 | 0.038 | probable gluconokinase |  | K00851 | Cytoplasm. | 45D |
| KLMA_R420 | -10.4 | 3.3 | 0.005 | His-tRNA |  |  |  | S |
| KLMA_50501 | -9.7 | 7.7 | 0.001 | peptidyl-prolyl cis-trans isomerase | CPR1 | K01802 | Cytoplasm. | S |
| KLMA_70444 | -9.1 | 6.1 | 0.001 | citrate synthase 3 | CIT3 | K01647 |  | 45D |
| KLMA_10008 | -8.9 | -0.1 | 0.029 | siderophore iron transporter ARN2 | ARN2 |  | Endosome membrane; Multi-pass membrane protein (By similarity). | S |
| KLMA_20830 | -7.7 | 8.8 | 0.000 | lactose permease | LAC12 |  | Membrane; Multi-pass membrane protein. | 45D |
| KLMA_30142 | -7.2 | 6.0 | 0.000 | serine/threonine-protein phosphatase PP1-2 | GLC7 | K06269 | Cytoplasm. Nucleus. | C |
| KLMA_50531 | -7.0 | 9.1 | 0.000 | uncharacterized transporter YLR152C |  | K07088 | Membrane; Multi-pass membrane protein. | 45D |
| KLMA_60047 | -6.6 | 9.4 | 0.003 | uncharacterized protein YGR235C | MOS2 |  | Mitochondrion inner membrane; Multi-pass membrane protein. | 45D |
| KLMA_70355 | -6.5 | 8.4 | 0.019 | uncharacterized protein YBL029C-A |  |  | Cell membrane; Peripheral membrane protein. | 45D |
| KLMA_10829 | -6.5 | 5.6 | 0.003 | hypothetical protein |  |  |  | 45D |
| KLMA_20832 | -6.2 | 5.9 | 0.000 | dethiobiotin synthetase | BIO4 | K01935 |  | 45D |
| KLMA_30728 | -5.8 | 6.0 | 0.000 | lactose permease | LAC12 |  | Membrane; Multi-pass membrane protein. | S |
| KLMA_10518 | -5.8 | 12.9 | 0.000 | inulinase | INU1 | K01193 | Secreted. | 45D |
| KLMA_30719 | -5.8 | 7.7 | 0.000 | phosphoenolpyruvate carboxykinase [ATP] | PCK1 | K01610 |  | 45D |
| KLMA_40260 | -5.6 | 3.1 | 0.004 | hypothetical protein |  |  |  | 30X |
| KLMA_10299 | -5.6 | 1.2 | 0.036 | spore wall maturation protein DIT1 | DIT1 |  |  | S |
| KLMA_30399 | -5.5 | 5.7 | 0.008 | conserved hypothetical membrane protein |  |  |  | S |
| KLMA_70433 | -5.5 | 1.5 | 0.025 | conserved hypothetical protein |  |  |  | S |
| KLMA_20831 | -5.5 | 1.1 | 0.039 | adenosylmethionine-8-amino-7-oxononanoate aminotransferase | BIO3 | K00833 |  | 30X |
| KLMA_40583 | -5.3 | 5.3 | 0.005 | D-lactate dehydrogenase [cytochrome] 1 | DLD1 | K00102 | Mitochondrion inner membrane. | S |
| KLMA_60263 | -5.3 | 5.5 | 0.026 | cholinephosphotransferase 1 | CPT1 | K00993 | Microsome membrane; Multi-pass membrane protein. Endoplasmic reticulum membrane; Multi-pass membrane protein. Mitochondrion outer membrane; Multi-pass membrane protein. | S |
| KLMA_60492 | -5.2 | 9.0 | 0.017 | ATPase inhibitor |  |  |  | 45D |
| KLMA_20333 | -5.2 | 9.4 | 0.000 | galactokinase | GAL1 | K00849 |  | 45D |
| KLMA_70303 | -5.1 | 4.9 | 0.040 | probable 6-phosphofructo-2-kinase/fructose-2,6-biphosphatase |  |  |  | 45D |
| KLMA_30619 | -5.0 | 9.7 | 0.000 | meiotic recombination protein REC8 | REC8 | K12780 | Nucleus. Chromosome. Chromosome, centromere. | S |
| KLMA_R606 | -5.0 | 7.7 | 0.043 | Thr-tRNA |  |  |  | S |
| KLMA_50151 | -5.0 | 6.1 | 0.025 | aromatic amino acid aminotransferase 1 | ARO8 |  | Cytoplasm. | 30X |
| KLMA_30148 | -5.0 | 6.2 | 0.032 | acetolactate synthase small subunit | ILV6 | K01653 | Mitochondrion. | 45D |
| KLMA_40214 | -5.0 | 2.6 | 0.049 | SUR7 family protein FMP45 | FMP45 |  | Cell membrane; Multi-pass membrane protein. | S |
| KLMA_40132 | -4.9 | 9.4 | 0.014 | transcription activator |  |  |  | S |
| KLMA_40371 | -4.8 | 10.0 | 0.000 | protein TMA108 | TMA108 |  | Cytoplasm. | S |
| KLMA_30398 | -4.8 | 8.5 | 0.015 | 2-deoxyglucose-6-phosphate phosphatase 2 | DOG2 | K01111 |  | 45D |
| KLMA_20829 | -4.8 | 3.7 | 0.042 | repressible acid phosphatase | PHO3 |  |  | S |
| KLMA_10121 | -4.8 | 8.4 | 0.001 | metal homeostasis factor ATX1 | ATX1 | K07213 | Cytoplasm. | S |
| KLMA_30672 | -4.7 | 6.0 | 0.029 | probable metabolite transport protein C1271.09 |  |  | Membrane; Multi-pass membrane protein (Potential). | 30X |
| KLMA_30166 | -4.7 | 4.9 | 0.004 | protein SIP4 | SIP4 |  | Nucleus (Probable). | S |
| KLMA_10827 | -4.7 | 5.6 | 0.001 | MFS_1 |  |  | Membrane; Multi-pass membrane protein. | 45D |
| KLMA_R205 | -4.7 | 5.7 | 0.013 | Leu-tRNA |  |  |  | S |
| KLMA_50449 | -4.6 | 7.6 | 0.006 | fungal_trans super family conserved domain |  |  |  | C |
| KLMA_70178 | -4.5 | 12.0 | 0.000 | uncharacterized protein YIL057C | RGI1 |  | Cell membrane; Peripheral membrane protein (By similarity). | 45D |
| KLMA_40244 | -4.4 | 7.1 | 0.001 | ATP-dependent permease PDR15 | PDR5 |  | Cell membrane; Multi-pass membrane protein. | S |
| KLMA_70044 | -4.3 | 9.8 | 0.000 | sorbitol dehydrogenase 1 | SOR1 | K00008 |  | 45D |
| KLMA_70189 | -4.3 | 8.6 | 0.027 | protein MMF1 | MMF1 |  | Mitochondrion matrix. | 45D |
| KLMA_60300 | -4.3 | 2.6 | 0.023 | outer spore wall protein 2 | OSW2 |  | Cytoplasm. Prospore membrane. | S |
| KLMA_70134 | -4.3 | 6.8 | 0.005 | protein transport protein SFT2 | SFT2 |  | Golgi apparatus membrane; Multi-pass membrane protein. | 30X |
| KLMA_80174 | -4.2 | 8.9 | 0.004 | probable transporter AQR1 | AQR1 |  | Membrane; Multi-pass membrane protein. | C |
| KLMA_30325 | -4.2 | 6.6 | 0.044 | cystathionine beta-lyase | STR3 | K01760 | Cytoplasm. Nucleus. | S |
| KLMA_30497 | -4.1 | 6.4 | 0.004 | 1,3-beta-glucan synthase component GSC2 | FKS3 |  | Mitochondrion. Membrane; Multi-pass membrane protein (Potential). | S |
| KLMA_60171 | -4.1 | 4.1 | 0.044 | protein SOV1 | SOV1 |  | Mitochondrion. | S |
| KLMA_60042 | -4.1 | 8.2 | 0.002 | peroxisomal membrane protein PEX21 | PEX21 |  | Cytoplasm (By similarity). Peroxisome membrane; Peripheral membrane protein; Cytoplasmic side (By similarity). | 45D |
| KLMA_20007 | -4.1 | 5.5 | 0.027 | putative monooxygenase yxeK | yxeK |  |  | S |
| KLMA_30718 | -4.0 | 5.2 | 0.005 | hypothetical protein |  |  |  | S |
| KLMA_80322 | -4.0 | 8.9 | 0.006 | cytochrome c oxidase subunit 7 |  | K02269 |  | 45D |
| KLMA_30331 | -4.0 | 6.5 | 0.006 | hypothetical protein |  |  |  | 45D |
| KLMA_40622 | -4.0 | 11.7 | 0.000 | hypothetical protein |  |  |  | C |
| KLMA_60413 | -4.0 | 6.3 | 0.006 | uncharacterized esterase/lipase C417.12 |  |  | Cytoplasm. Nucleus. | S |
| KLMA_70459 | -4.0 | 5.2 | 0.010 | probable transporter MCH2 | MCH2 |  | Membrane; Multi-pass membrane protein. | S |
| KLMA_10440 | -3.9 | 5.4 | 0.007 | hypothetical protein |  |  | Membrane; Multi-pass membrane protein (Potential). | S |
| KLMA_20004 | -3.9 | 5.7 | 0.009 | oligopeptide transporter 1 | OPT1 |  | Cell membrane; Multi-pass membrane protein. | S |
| KLMA_20489 | -3.8 | 9.0 | 0.008 | GAL4-like Zn2Cys6 binuclear cluster DNA-binding domain |  |  |  | C |
| KLMA_30496 | -3.8 | 6.0 | 0.010 | TPR repeat-containing protein associated with Hsp90 | TAH1 |  | Cytoplasm. Nucleus. | 30X |
| KLMA_50409 | -3.8 | 7.4 | 0.008 | flo11 super family | MUC1 | K01178 | Secreted, cell wall (Probable). Membrane; Lipid-anchor, GPI-anchor (Potential). | 45D |
| KLMA_60412 | -3.8 | 10.7 | 0.001 | hexokinase | RAG5 | K00844 |  | C |
| KLMA_80275 | -3.8 | 7.2 | 0.006 | non-disjunction protein 1 |  |  |  | S |
| KLMA_70443 | -3.8 | 3.9 | 0.044 | probable 2-methylcitrate dehydratase | PDH1 | K01720 |  | C |
| KLMA_30246 | -3.7 | 8.6 | 0.005 | sphingoid long-chain base transporter RSB1 | RSB1 |  | Cell membrane (By similarity); Multi-pass membrane protein. | S |
| KLMA_20704 | -3.7 | 9.8 | 0.001 | CBM_21 super family | GAC1 |  |  | 45D |
| KLMA_40201 | -3.7 | 8.0 | 0.007 | zinc finger protein YPR013C |  |  |  | S |
| KLMA_20222 | -3.6 | 4.4 | 0.017 | GAL4 |  |  |  | S |
| KLMA_30072 | -3.6 | 5.0 | 0.025 | poly(A) polymerase | PAP1 | K14376 | Nucleus. | S |
| KLMA_30640 | -3.5 | 8.3 | 0.043 | D-amino-acid oxidase | dao1 |  |  | C |
| KLMA_20822 | -3.5 | 5.2 | 0.019 | dnaJ-like chaperone JEM1 |  | K09523 |  | S |
| KLMA_20442 | -3.5 | 11.6 | 0.000 | ubiquitin | UBI4 | K08770 | Cytoplasm (By similarity). Nucleus (By similarity). | S |
| KLMA_R128 | -3.5 | 6.0 | 0.014 | Ser-tRNA |  |  |  | S |
| KLMA_30237 | -3.5 | 7.7 | 0.030 | protein MTH1 | MTH1 |  |  | S |
| KLMA_20010 | -3.5 | 4.4 | 0.050 | protein crtK |  |  |  | S |
| KLMA_R530 | -3.5 | 5.1 | 0.039 | Gln-tRNA |  |  |  | 30X |
| KLMA_40341 | -3.5 | 8.3 | 0.008 | hypothetical protein |  |  |  | S |
| KLMA_60520 | -3.4 | 7.8 | 0.017 | ferric/cupric reductase transmembrane component 1 | FRE1 |  | Cell membrane; Multi-pass membrane protein. | S |
| KLMA_40128 | -3.4 | 10.1 | 0.001 | heat shock protein 26 | HSP26 | K13993 |  | 30X |
| KLMA_70213 | -3.4 | 7.1 | 0.026 | dilute domain-containing protein YPR089W |  | K06867 | Golgi apparatus. | S |
| KLMA_50067 | -3.4 | 6.2 | 0.024 | probable kynurenine--oxoglutarate transaminase BNA3 | BNA3 | K14264 | Cytoplasm. Mitochondrion. | S |
| KLMA_30165 | -3.4 | 4.8 | 0.018 | DNA replication regulator DPB11 | DPB11 | K03507 | Nucleus. | S |
| KLMA_60392 | -3.4 | 9.4 | 0.003 | uncharacterized protein YPL039W |  |  |  | C |
| KLMA_40294 | -3.3 | 6.4 | 0.039 | protein SYM1 | SYM1 | K13348 | Mitochondrion inner membrane; Multi-pass membrane protein (By similarity). | S |
| KLMA_30729 | -3.3 | 6.1 | 0.026 | putative uncharacterized oxidoreductase YGL039W |  |  |  | S |
| KLMA_60379 | -3.3 | 6.5 | 0.025 | recQ-mediated genome instability protein 1 | RMI1 | K15364 | Cytoplasm. Nucleus. | 45D |
| KLMA_80130 | -3.3 | 6.8 | 0.020 | homoisocitrate dehydrogenase | LYS12 | K05824 | Mitochondrion. | C |
| KLMA_10187 | -3.3 | 5.0 | 0.025 | cell division control protein 15 | CDC15 | K06683 | Cytoplasm, cytoskeleton, spindle pole. | S |
| KLMA_60414 | -3.3 | 4.1 | 0.040 | allantoate permease | DAL5 |  | Membrane; Multi-pass membrane protein. | S |
| KLMA_70042 | -3.3 | 5.9 | 0.025 | uncharacterized protein YFL042C |  |  | Cytoplasmic vesicle membrane; Single-pass membrane protein (Potential). | S |
| KLMA_10514 | -3.3 | 10.4 | 0.002 | branched-chain-amino-acid aminotransferase | BAT1 | K00826 | Mitochondrion matrix. | C |
| KLMA_30689 | -3.3 | 7.4 | 0.037 | SIT4-associating protein SAP155 | SAP155 | K15457 | Cytoplasm. | S |
| KLMA_50241 | -3.3 | 7.4 | 0.018 | pyridoxamine 5'-phosphate oxidase | PDX3 | K00275 |  | C |
| KLMA_20597 | -3.3 | 5.7 | 0.027 | transaminated amino acid decarboxylase | ARO10 | K12732 | Cytoplasm. | 30X |
| KLMA_40372 | -3.2 | 10.5 | 0.002 | J domain-containing protein APJ1 | APJ1 |  | Cytoplasm. Nucleus. | S |
| KLMA_30644 | -3.2 | 4.4 | 0.038 | protein PNS1 | PNS1 |  | Cell membrane; Multi-pass membrane protein (By similarity). | S |
| KLMA_20826 | -3.2 | 5.0 | 0.027 | pantothenate transporter FEN2 | FEN2 | K03448 | Cell membrane; Multi-pass membrane protein. | S |
| KLMA_10547 | -3.2 | 8.7 | 0.012 | high-affinity glucose transporter | HGT1 |  | Membrane; Multi-pass membrane protein. | 45D |
| KLMA_40126 | -3.2 | 6.9 | 0.026 | UDP-N-acetylglucosamine transferase subunit ALG14 | ALG14 | K07441 | Endoplasmic reticulum membrane; Single-pass membrane protein (By similarity). | S |
| KLMA_20326 | -3.2 | 6.6 | 0.026 | riboflavin transporter MCH5 | MCH5 |  | Cell membrane; Multi-pass membrane protein (Potential). | S |
| KLMA_40057 | -3.1 | 7.7 | 0.033 | interstrand crosslink repair protein | PSO2 | K15340 | Nucleus (Probable). | S |
| KLMA_80005 | -3.1 | 5.7 | 0.028 | high-affinity glucose transporter | LAC12 |  | Membrane; Multi-pass membrane protein. | S |
| KLMA_40149 | -3.1 | 9.1 | 0.011 | hypothetical protein |  |  |  | 45D |
| KLMA_50587 | -3.1 | 5.3 | 0.046 | peroxisomal 2 | SPS19 | K13237 | Peroxisome. | S |
| KLMA_60268 | -3.1 | 7.8 | 0.019 | uncharacterized protein YNL134C |  |  |  | S |
| KLMA_50482 | -3.1 | 11.5 | 0.001 | non-classical export protein 2 | NCE102 |  | Cell membrane; Multi-pass membrane protein. | 45D |
| KLMA_40045 | -3.1 | 11.5 | 0.000 | ATPase-stabilizing factor 15 kDa protein | STF2 |  | Mitochondrion. | 45D |
| KLMA_80246 | -3.1 | 8.7 | 0.036 | nuclear polyadenylated RNA-binding protein 4 | HRP1 | K14411 | Cytoplasm. Nucleus. | S |
| KLMA_10558 | -3.0 | 9.6 | 0.008 | D-arabinitol 2-dehydrogenase [ribulose-forming] | ARDH |  |  | 45D |
| KLMA_30715 | -3.0 | 8.2 | 0.026 | LCB5 |  |  |  | S |
| KLMA_10036 | -3.0 | 5.5 | 0.045 | pantothenate kinase | CAB1 | K09680 | Cytoplasm. Nucleus. | C |
| KLMA_40107 | -3.0 | 8.2 | 0.023 | phospho-2-dehydro-3-deoxyheptonate aldolase | ARO3 | K01626 |  | S |
| KLMA_50125 | -3.0 | 8.8 | 0.039 | sterol regulatory element-binding protein ECM22 | UPC2 |  | Nucleus. | S |
| KLMA_20615 | -3.0 | 8.9 | 0.017 | YCII super family |  | K09780 |  | S |
| KLMA_50402 | -2.9 | 8.5 | 0.032 | 2-isopropylmalate synthase | LEU4 | K01649 | Isoform Cytoplasmic: Cytoplasm.Isoform Mitochondrial: Mitochondrion. | 45D |
| KLMA_20515 | -2.9 | 6.7 | 0.047 | protein ISD11 | ISD11 |  | Mitochondrion. | 45D |
| KLMA_50101 | -2.9 | 7.0 | 0.046 | SWIRM domain-containing protein YOR338W |  |  |  | S |
| KLMA_20220 | -2.9 | 13.9 | 0.000 | 3-ketoacyl-CoA thiolase | POT1 | K00632 | Peroxisome. | 45D |
| KLMA_20042 | -2.8 | 8.7 | 0.026 | acetyl-CoA hydrolase | ACH1 | K01067 | Cytoplasm (By similarity). | 45D |
| KLMA_10487 | -2.8 | 9.6 | 0.023 | probable serine/threonine-protein kinase YMR291W | TDA1 | K08286 | Cytoplasm. Nucleus. | S |
| KLMA_40218 | -2.8 | 13.1 | 0.000 | glyceraldehyde-3-phosphate dehydrogenase 1 | GAP1 | K00134 | Cytoplasm (By similarity). | 45D |
| KLMA_20503 | -2.8 | 10.0 | 0.026 | DNA polymerase epsilon subunit B | DPB2 | K02325 | Nucleus (By similarity). | S |
| KLMA_40270 | -2.8 | 9.1 | 0.026 | dual specificity protein kinase KNS1 | KNS1 | K08287 |  | S |
| KLMA_20475 | -2.8 | 7.4 | 0.049 | protein ROD1 | ROD1 |  | Membrane; Peripheral membrane protein. | S |
| KLMA_50537 | -2.7 | 11.2 | 0.002 | eukaryotic peptide chain release factor GTP-binding subunit | SUP35 | K03267 | Cytoplasm (Probable). | S |
| KLMA_20100 | -2.6 | 8.8 | 0.043 | reduced viability upon starvation protein 161 | RVS161 |  | Cytoplasm, cytoskeleton. | 45D |
| KLMA_80119 | -2.6 | 11.8 | 0.008 | histone H3 | HHT1 | K11253 | Nucleus (By similarity). Chromosome (By similarity). | 45D |
| KLMA_60167 | -2.6 | 12.1 | 0.002 | malate dehydrogenase | MDH1 | K00026 | Mitochondrion matrix. | 45D |
| KLMA_50289 | -2.6 | 9.2 | 0.042 | vacuolar membrane protein YOR292C |  |  | Vacuole membrane; Multi-pass membrane protein. | 45D |
| KLMA_80159 | -2.5 | 9.9 | 0.037 | cytochrome c oxidase polypeptide 5A | COX5A | K02263 | Mitochondrion inner membrane. | S |
| KLMA_20440 | -2.3 | 10.5 | 0.023 | pH-response transcription factor pacC/RIM101 | RIM101 |  | Cytoplasm (By similarity). Nucleus (By similarity). | S |
| KLMA_10040 | -1.9 | 19.0 | 0.000 | uncharacterized protein YDR524C-B |  |  |  | 45D |
| KLMA_10830 | -1.9 | 12.3 | 0.017 | ribosyldihydronicotinamide dehydrogenase [quinone] | Nqo2 |  | Cytoplasm (By similarity). | S |
| KLMA_60129 | -1.8 | 13.0 | 0.010 | histone H3 | HHT1 | K11253 | Nucleus (By similarity). Chromosome (By similarity). | 45D |

^a^Gene expression was significantly (FDR < 0.05) altered under the following conditions: S, 30DS-specific down-regulation; C, commonly down-regulated under 30DS, 45D and 30X conditions; 45D, down-regulated under 45D and 30DS conditions; 30X, down-regulated under 30X and 30DS conditions.

**Table S13** GO terms enriched in significantly down-regulated genes under 45D condition

| GO.ID | Term | Annotated  gene^a^ | Significant^b^ | Expected^c^ | P-value^d^ | Genes |
| --- | --- | --- | --- | --- | --- | --- |
| GO:0005975 | carbohydrate metabolic process | 247 | 44 | 25.86 | 0.0002 | ACN9, ALG3, ARDH, BUD32, BUD7, CAT5, CIT3, CRH1, DFG5, DOG2, ENO, EOS1, GAC1, GAL1, GAP1, GAP3, GAS5, GID8, GLC7, GLO4, GPD1, INU1, KLMA_20548, KLMA_30562, KLMA_40105, KLMA_60382, KTR1, KTR5, MDH1, MIOX5, OCH1, PCK1, PCM1, PGK, PKP2, RAG2, RAG5, RER2, RKI1, RMD5, SOR1, STT3, SVP26, XYL1 |
| GO:0009097 | isoleucine biosynthetic process | 9 | 5 | 0.94 | 0.0011 | BAT1, ILV1, ILV2, ILV6, MMF1 |
| GO:0044281 | small molecule metabolic process | 645 | 90 | 67.52 | 0.0012 | ACP1, ADI1, ALA1, APA2, ARDH, ARO7, ATF1, ATP3, BAT1, BDH1, BIO4, CAB1, CAT2, CAT5, CIT3, COQ1, COQ5, CYB5, DTD1, DUG3, EFT1, FAA2, FMN1, FOL2, FUM1, FUR1, GAD1, GCV1, GDI1, GID8, GLO4, GRS1, HAM1, HIS1, HIS7, IDI1, ILS1, ILV1, ILV2, ILV6, IOC2, IOC4, ISN1, IZH2, KLMA_10627, KLMA_10805, KLMA_30357, KLMA_40483, KLMA_40623, KLMA_60382, LEU4, LYS12, LYS9, MDH1, MEF1, MET17, MET22, MIOX5, MMF1, MRS6, MST1, OCA5, PCD1, PCM1, PDH1, PDR17, PDX3, PHA2, POT1, PRO2, PRS3, RDI1, RER2, RIB4, RKI1, RMD5, SAM4, SER2, SNZ3, TEF, THI20, TRP3, TYR1, URA1, URE2, YFH1, YRB2, gabD, gcp |
| GO:0005996 | monosaccharide metabolic process | 81 | 18 | 8.48 | 0.0014 | ACN9, CAT5, DOG2, ENO, GAL1, GAP1, GAP3, GID8, MIOX5, PCK1, PGK, PKP2, RAG2, RAG5, RKI1, RMD5, SOR1, XYL1 |
| GO:0009082 | branched-chain amino acid biosynthetic process | 14 | 6 | 1.47 | 0.0018 | BAT1, ILV1, ILV2, ILV6, LEU4, MMF1 |
| GO:0006549 | isoleucine metabolic process | 11 | 5 | 1.15 | 0.0033 | BAT1, ILV1, ILV2, ILV6, MMF1 |
| GO:0019318 | hexose metabolic process | 74 | 16 | 7.75 | 0.0034 | ACN9, CAT5, DOG2, ENO, GAL1, GAP1, GAP3, GID8, PCK1, PGK, PKP2, RAG2, RAG5, RKI1, RMD5, SOR1 |
| GO:0042822 | pyridoxal phosphate metabolic process | 4 | 3 | 0.42 | 0.0042 | BUD17, KLMA_70278, SNZ3 |
| GO:0042823 | pyridoxal phosphate biosynthetic process | 4 | 3 | 0.42 | 0.0042 | BUD17, KLMA_70278, SNZ3 |
| GO:0046184 | aldehyde biosynthetic process | 4 | 3 | 0.42 | 0.0042 | BUD17, KLMA_70278, SNZ3 |
| GO:0019752 | carboxylic acid metabolic process | 345 | 51 | 36.12 | 0.0053 | ACP1, ADI1, ALA1, ARO7, BAT1, BIO4, CAT2, CIT3, DTD1, DUG3, FAA2, FOL2, FUM1, GAD1, GCV1, GLO4, GRS1, HIS1, HIS7, ILS1, ILV1, ILV2, ILV6, IZH2, KLMA_10627, KLMA_10805, KLMA_30357, KLMA_40623, KLMA_60382, LEU4, LYS12, LYS9, MDH1, MET17, MET22, MIOX5, MMF1, MST1, PDH1, PDR17, PDX3, PHA2, POT1, PRO2, SAM4, SER2, TRP3, TYR1, YFH1, gabD |
| GO:0006006 | glucose metabolic process | 64 | 14 | 6.7 | 0.0053 | ACN9, CAT5, DOG2, ENO, GAP1, GAP3, GID8, PCK1, PGK, PKP2, RAG2, RAG5, RKI1, RMD5 |
| GO:0044723 | single-organism carbohydrate metabolic process | 202 | 33 | 21.15 | 0.0054 | ACN9, ALG3, ARDH, BUD32, BUD7, CAT5, DOG2, ENO, EOS1, GAC1, GAL1, GAP1, GAP3, GID8, GLC7, KLMA_60382, KTR1, KTR5, MIOX5, OCH1, PCK1, PCM1, PGK, PKP2, RAG2, RAG5, RER2, RKI1, RMD5, SOR1, STT3, SVP26, XYL1 |
| GO:0043436 | oxoacid metabolic process | 354 | 52 | 37.06 | 0.0055 | ACP1, ADI1, ALA1, ARO7, BAT1, BIO4, CAT2, CIT3, DTD1, DUG3, FAA2, FOL2, FUM1, GAD1, GCV1, GLO4, GRS1, HIS1, HIS7, ILS1, ILV1, ILV2, ILV6, IZH2, KLMA_10627, KLMA_10805, KLMA_30357, KLMA_40623, KLMA_60382, LEU4, LYS12, LYS9, MDH1, MET17, MET22, MIOX5, MMF1, MST1, PDH1, PDR17, PDX3, PHA2, POT1, PRO2, SAM4, SER2, TRP3, TYR1, URE2, YFH1, gabD |
| GO:0006082 | organic acid metabolic process | 356 | 52 | 37.27 | 0.0062 | ACP1, ADI1, ALA1, ARO7, BAT1, BIO4, CAT2, CIT3, DTD1, DUG3, FAA2, FOL2, FUM1, GAD1, GCV1, GLO4, GRS1, HIS1, HIS7, ILS1, ILV1, ILV2, ILV6, IZH2, KLMA_10627, KLMA_10805, KLMA_30357, KLMA_40623, KLMA_60382, LEU4, LYS12, LYS9, MDH1, MET17, MET22, MIOX5, MMF1, MST1, PDH1, PDR17, PDX3, PHA2, POT1, PRO2, SAM4, SER2, TRP3, TYR1, URE2, YFH1, gabD |
| GO:0051188 | cofactor biosynthetic process | 80 | 16 | 8.37 | 0.0075 | ACP1, BUD17, CAB1, CAT5, COQ1, COQ5, COX10, COX15, FMN1, FOL2, HEM12, HEM4, KLMA_70278, SNZ3, THI20, YFH1 |
| GO:0044283 | small molecule biosynthetic process | 263 | 40 | 27.53 | 0.0082 | ACP1, ADI1, ARO7, BAT1, BDH1, BIO4, CAT5, COQ1, COQ5, CYB5, FMN1, FOL2, GLO4, HIS1, HIS7, IDI1, ILV1, ILV2, ILV6, KLMA_30357, KLMA_40623, LEU4, LYS12, LYS9, MET17, MET22, MIOX5, MMF1, PDR17, PDX3, PHA2, PRO2, RER2, RIB4, SAM4, SER2, SNZ3, THI20, TRP3, TYR1 |
| GO:0009095 | aromatic amino acid family biosynthetic process, prephenate pathway | 5 | 3 | 0.52 | 0.0097 | ARO7, PHA2, TYR1 |
| GO:0009099 | valine biosynthetic process | 5 | 3 | 0.52 | 0.0097 | BAT1, ILV2, ILV6 |

^a^The number of GO term annotated genes in the *K. marxianus* genome.

^b^The number of GO term annotated genes, which were significantly (FDR < 0.05) expressed under the condition.

^c^The expected value of Fisher's exact test.

^d^The P-value of Fisher's exact test.

**Table S14** Summary of significantly down-regulated genes under the 45D condition

| Locus_tag | logFC | logCPM | FDR | Product | UniProt gene | KO number | Localization | Specific^a^ |
| --- | --- | --- | --- | --- | --- | --- | --- | --- |
| KLMA_60492 | -18.7 | 9.0 | 0.000 | ATPase inhibitor |  |  |  | 30DS |
| KLMA_50402 | -18.6 | 8.5 | 0.000 | 2-isopropylmalate synthase | LEU4 | K01649 | Isoform Cytoplasmic: Cytoplasm.Isoform Mitochondrial: Mitochondrion. | 30DS |
| KLMA_50016 | -18.0 | 9.0 | 0.000 | (2R,3R)-2,3-butanediol dehydrogenase | BDH1 | K00004 | Cytoplasm. | S |
| KLMA_70355 | -17.9 | 8.4 | 0.000 | uncharacterized protein YBL029C-A |  |  | Cell membrane; Peripheral membrane protein. | 30DS |
| KLMA_20783 | -17.6 | 8.2 | 0.000 | 60S ribosomal protein L33-B | RPL33B | K02917 | Cytoplasm. | S |
| KLMA_60402 | -17.1 | 6.6 | 0.000 | pisatin demethylase | PDAT9 | K00493 |  | 30X |
| KLMA_60530 | -16.9 | 6.9 | 0.000 | mitochondrial import receptor subunit TOM7 |  |  |  | S |
| KLMA_50407 | -16.8 | 7.0 | 0.000 | UPF0662 protein YPL260W |  |  | Cytoplasm. Nucleus. | S |
| KLMA_60022 | -16.7 | 7.9 | 0.000 | protein PET10 | PET10 |  | Lipid droplet. Membrane; Peripheral membrane protein. | S |
| KLMA_30148 | -16.5 | 6.2 | 0.000 | acetolactate synthase small subunit | ILV6 | K01653 | Mitochondrion. | 30DS |
| KLMA_40593 | -16.5 | 7.2 | 0.000 | uncharacterized vacuolar membrane protein YML018C |  | K15289 | Vacuole membrane; Multi-pass membrane protein. | S |
| KLMA_20491 | -16.3 | 7.2 | 0.000 | cytochrome c oxidase subunit 4 | COX4 | K02265 | Mitochondrion inner membrane. | S |
| KLMA_20726 | -15.9 | 5.7 | 0.000 | D-tyrosyl-tRNA(Tyr) deacylase | DTD1 | K07560 | Cytoplasm (By similarity). | S |
| KLMA_50231 | -15.9 | 6.6 | 0.000 | aminomethyltransferase | GCV1 | K00605 | Mitochondrion. | S |
| KLMA_50143 | -15.8 | 5.7 | 0.001 | 26S protease regulatory subunit 8 homolog | RPT6 | K03066 | Cytoplasm (Potential). Nucleus (Potential). | S |
| KLMA_80028 | -15.8 | 7.7 | 0.000 | fumarate hydratase | FUM1 | K01679 | Mitochondrion matrix. Cytoplasm. | S |
| KLMA_30554 | -15.7 | 6.0 | 0.000 | cell wall protein ECM33 | ECM33 |  | Cell membrane; Lipid-anchor, GPI-anchor. Secreted, cell wall. | S |
| KLMA_10763 | -15.7 | 7.0 | 0.000 | glucose-6-phosphate isomerase | RAG2 | K01810 | Cytoplasm. | S |
| KLMA_80057 | -15.7 | 7.8 | 0.000 | eukaryotic translation initiation factor 2 subunit alpha | SUI2 | K03237 |  | S |
| KLMA_40016 | -15.6 | 6.0 | 0.000 | saccharopine dehydrogenase [NADP+ | LYS9 | K00293 |  | S |
| KLMA_30643 | -15.6 | 5.6 | 0.000 | IMP-specific 5'-nucleotidase 1 | ISN1 |  |  | S |
| KLMA_80241 | -15.6 | 5.8 | 0.000 | jmjC domain-containing protein 4 |  |  |  | S |
| KLMA_50237 | -15.5 | 6.6 | 0.000 | ATP synthase subunit gamma | ATP3 | K02136 | Mitochondrion. Mitochondrion inner membrane; Peripheral membrane protein (Probable). | S |
| KLMA_60233 | -15.5 | 6.1 | 0.009 | cytochrome b5 | CYB5 |  | Endoplasmic reticulum membrane; Single-pass membrane protein; Cytoplasmic side (By similarity). Microsome membrane; Single-pass membrane protein; Cytoplasmic side (By similarity). | S |
| KLMA_30515 | -15.5 | 6.5 | 0.000 | pre-mRNA-splicing factor CWC2 | CWC2 |  | Nucleus (By similarity). | S |
| KLMA_30259 | -15.4 | 6.1 | 0.000 | altered inheritance rate of mitochondria protein 29 | AIM29 |  | Cytoplasm. | S |
| KLMA_70435 | -15.4 | 6.0 | 0.000 | probable electron transfer flavoprotein subunit alpha | AIM45 | K03522 | Mitochondrion matrix (By similarity). | S |
| KLMA_40572 | -15.3 | 6.1 | 0.000 | HIG1 domain-containing protein YML030W | RCF1 |  | Mitochondrion membrane; Multi-pass membrane protein (By similarity). | S |
| KLMA_70351 | -15.3 | 6.9 | 0.004 | KH domain-containing protein YBL032W | HEK2 |  | Cytoplasm (By similarity). Cytoplasm, P-body (By similarity). Nucleus (By similarity). Chromosome, telomere (By similarity). | S |
| KLMA_20536 | -15.3 | 4.8 | 0.000 | yjgF_YER057c_UK114_family |  |  |  | C |
| KLMA_40148 | -15.3 | 6.6 | 0.001 | putative aryl-alcohol dehydrogenase YPL088W |  |  |  | S |
| KLMA_50202 | -15.2 | 4.9 | 0.000 | DUF3445 super family conserved domain |  |  |  | S |
| KLMA_80089 | -15.1 | 6.3 | 0.000 | translation machinery-associated protein 17 | TMA17 |  | Cytoplasm. Nucleus. | 30DS |
| KLMA_10819 | -15.1 | 6.1 | 0.000 | prephenate dehydrogenase [NADP+] | TYR1 | K00211 |  | S |
| KLMA_80363 | -15.1 | 5.3 | 0.000 | protein AST1 | AST1 |  | Membrane; Peripheral membrane protein. | S |
| KLMA_20756 | -15.0 | 6.7 | 0.000 | bud neck protein 5 | BNI5 |  | Cytoplasm. Bud. | S |
| KLMA_10322 | -14.9 | 5.5 | 0.000 | hypothetical protein | CAF20 | K03261 | Cytoplasm (By similarity). | S |
| KLMA_60026 | -14.9 | 6.3 | 0.000 | UPF0615 protein YJL123C | MTC1 |  | Cytoplasm. Cytoplasmic vesicle, COPI-coated vesicle. | S |
| KLMA_80182 | -14.8 | 5.7 | 0.003 | ER-derived vesicles protein ERV29 | ERV29 |  | Endoplasmic reticulum membrane; Multi-pass membrane protein. | S |
| KLMA_50566 | -14.8 | 5.5 | 0.000 | methionine aminopeptidase 1 | MAP1 | K01265 |  | S |
| KLMA_50134 | -14.7 | 6.2 | 0.000 | GTP-binding nuclear protein GSP1/Ran | GSP1 | K07936 | Nucleus (By similarity). | S |
| KLMA_10811 | -14.7 | 5.3 | 0.000 | translocation protein SEC66 | SEC66 | K12273 | Endoplasmic reticulum membrane; Single-pass type II membrane protein. | S |
| KLMA_30093 | -14.7 | 5.7 | 0.000 | mitochondrial oxaloacetate transport protein | OAC1 | K15117 | Mitochondrion inner membrane; Multi-pass membrane protein (Potential). | S |
| KLMA_80332 | -14.7 | 5.1 | 0.000 | chorismate mutase | ARO7 | K01850 |  | S |
| KLMA_70093 | -14.6 | 4.6 | 0.000 | carboxypeptidase S | CPS1 |  | Vacuole membrane; Single-pass membrane protein. | 30X |
| KLMA_50500 | -14.6 | 4.1 | 0.001 | epsin-like protein | ENT5 |  | Cytoplasm. Endosome membrane; Peripheral membrane protein. | C |
| KLMA_20534 | -14.5 | 5.0 | 0.000 | aspartic proteinase 3 | YPS1 | K06009 | Cell membrane; Lipid-anchor, GPI-anchor. | S |
| KLMA_60542 | -14.5 | 5.4 | 0.000 | NADPH-dependent 1-acyldihydroxyacetone phosphate reductase | AYR1 | K06123 | Lipid droplet. Endoplasmic reticulum. | S |
| KLMA_30030 | -14.5 | 6.6 | 0.000 | cell division control protein 11 | CDC11 |  | Membrane. Bud neck. | S |
| KLMA_20071 | -14.4 | 4.8 | 0.000 | DUP super family |  |  |  | S |
| KLMA_60505 | -14.4 | 4.8 | 0.011 | ADP-ribosylation factor | ARF1 | K07977 | Golgi apparatus (By similarity). | S |
| KLMA_30617 | -14.3 | 6.0 | 0.000 | plasma membrane iron permease | FTR1 | K07243 | Membrane; Multi-pass membrane protein (Potential). | S |
| KLMA_10139 | -14.3 | 4.9 | 0.000 | pre-mRNA-splicing factor CWC25 | CWC25 |  | Nucleus (By similarity). | 30DS |
| KLMA_50247 | -14.3 | 6.8 | 0.000 | 54S ribosomal protein MRP49 | MRP49 |  | Mitochondrion. | S |
| KLMA_30556 | -14.2 | 4.7 | 0.000 | glutamate decarboxylase | GAD1 | K01580 |  | 30X |
| KLMA_40015 | -14.2 | 4.0 | 0.000 | protein OCA4 | OCA4 |  |  | S |
| KLMA_R701 | -14.2 | 5.1 | 0.000 | Gly-tRNA |  |  |  | S |
| KLMA_60501 | -14.1 | 6.3 | 0.000 | rho GDP-dissociation inhibitor | RDI1 | K12462 | Cytoplasm. | S |
| KLMA_50335 | -14.1 | 6.7 | 0.000 | transposon Ty1-H Gag-Pol polyprotein |  |  |  | S |
| KLMA_60028 | -14.0 | 4.8 | 0.004 | sm-like protein LSm1 | LSM1 | K12620 | Nucleus. Cytoplasm. Cytoplasm, P-body. | S |
| KLMA_30420 | -13.9 | 4.4 | 0.001 | v-type proton ATPase subunit G | VMA10 | K02152 |  | S |
| KLMA_60175 | -13.9 | 5.1 | 0.000 | v-type proton ATPase subunit C | VMA5 | K02148 | Vacuole membrane; Peripheral membrane protein. | S |
| KLMA_70356 | -13.9 | 5.3 | 0.000 | hypothetical protein |  |  |  | S |
| KLMA_30069 | -13.9 | 4.9 | 0.000 | DOA4-independent degradation protein 4 | DID4 | K12191 | Cytoplasm. Endosome membrane; Peripheral membrane protein. | S |
| KLMA_60169 | -13.8 | 5.4 | 0.000 | hypothetical protein | AVO2 |  | Cell membrane; Peripheral membrane protein; Cytoplasmic side. Vacuole membrane; Peripheral membrane protein; Cytoplasmic side. | S |
| KLMA_70260 | -13.8 | 6.7 | 0.000 | tubulin alpha-1 chain | TUB1 | K07374 | Cytoplasm, cytoskeleton. | S |
| KLMA_40390 | -13.7 | 4.6 | 0.000 | tRNA guanosine-2'-O-methyltransferase TRM13 | TRM13 | K15446 | Cytoplasm (By similarity). Nucleus, nucleolus (By similarity). | S |
| KLMA_70023 | -13.6 | 4.9 | 0.000 | pheromone-regulated membrane protein 4 | PRM4 |  | Membrane; Single-pass membrane protein (Potential). | S |
| KLMA_80042 | -13.6 | 4.7 | 0.000 | ADIPOR-like receptor IZH2 | IZH2 | K07036 | Membrane; Multi-pass membrane protein. | S |
| KLMA_50307 | -13.6 | 5.8 | 0.001 | predicted solute binding protein |  |  |  | S |
| KLMA_50599 | -13.6 | 4.1 | 0.000 | vacuolar import and degradation protein 27 | VID27 |  | Cytoplasm. | S |
| KLMA_80414 | -13.6 | 3.7 | 0.005 | single-stranded nucleic acid-binding protein | SBP1 |  | Cytoplasm. Nucleus, nucleolus. | 30X |
| KLMA_20413 | -13.5 | 3.1 | 0.001 | imidazole glycerol phosphate synthase hisHF | HIS7 | K01663 |  | S |
| KLMA_80030 | -13.4 | 3.9 | 0.000 | rRNA-processing protein UTP23 | UTP23 | K14773 | Mitochondrion. Nucleus, nucleolus. | S |
| KLMA_30067 | -13.4 | 4.0 | 0.001 | inositol phosphorylceramide synthase | AUR1 |  | Golgi apparatus, Golgi stack membrane; Multi-pass membrane protein. | S |
| KLMA_10233 | -13.4 | 4.5 | 0.000 | hypothetical protein |  |  |  | 30X |
| KLMA_10389 | -13.4 | 5.1 | 0.016 | farnesyl pyrophosphate synthetase | FPS1 | K00787 | Cytoplasm. | S |
| KLMA_70400 | -13.4 | 6.4 | 0.000 | coatomer subunit gamma | SEC21 |  | Cytoplasm. Golgi apparatus membrane; Peripheral membrane protein; Cytoplasmic side. Cytoplasmic vesicle, COPI-coated vesicle membrane; Peripheral membrane protein; Cytoplasmic side. Endosome. | S |
| KLMA_40287 | -13.4 | 6.0 | 0.000 | probable glycosidase CRH1 | CRH1 |  | Secreted, cell wall. Membrane; Lipid-anchor, GPI-anchor. | S |
| KLMA_20050 | -13.3 | 3.1 | 0.001 | inositol oxygenase 1 | MIOX5 | K00469 | Cytoplasm (Probable). | S |
| KLMA_20280 | -13.3 | 3.9 | 0.000 | zinc finger transcription factor YRR1 | YRM1 |  | Cytoplasm. Nucleus. | S |
| KLMA_70403 | -13.2 | 3.3 | 0.006 | 40S ribosomal protein MRP10 | MRP10 |  | Mitochondrion (By similarity). | 30X |
| KLMA_20712 | -13.2 | 3.1 | 0.001 | protein OPY1 | OPY1 |  |  | S |
| KLMA_10713 | -13.2 | 5.4 | 0.007 | vacuolar protein sorting-associated protein 55 | VPS55 |  | Endosome membrane; Multi-pass membrane protein (Potential). | S |
| KLMA_20238 | -13.2 | 4.3 | 0.007 | uncharacterized protein YGL108C |  |  |  | S |
| KLMA_50299 | -13.2 | 4.3 | 0.000 | peroxisomal membrane protein PAS20 | PEX13 | K13344 | Peroxisome membrane; Single-pass membrane protein. | S |
| KLMA_70031 | -13.1 | 4.5 | 0.000 | protein transport protein BOS1 | BOS1 | K08496 | Golgi apparatus membrane; Single-pass type IV membrane protein (By similarity). Endoplasmic reticulum membrane; Single-pass type IV membrane protein (By similarity). | S |
| KLMA_80316 | -13.1 | 3.6 | 0.002 | protein phosphatase 2C homolog 4 | PTC4 | K01090 |  | S |
| KLMA_20441 | -13.0 | 6.6 | 0.000 | cell wall integrity and stress response component 4 |  |  |  | S |
| KLMA_10677 | -13.0 | 3.3 | 0.000 | protein MET17 | MET17 | K01740 | Cytoplasm (By similarity). | S |
| KLMA_20181 | -13.0 | 3.9 | 0.000 | uroporphyrinogen decarboxylase | HEM12 | K01599 | Nucleus. Cytoplasm. | S |
| KLMA_50480 | -13.0 | 4.7 | 0.000 | mitochondrial import inner membrane translocase subunit TIM44 | TIM44 |  | Mitochondrion inner membrane. | S |
| KLMA_10732 | -13.0 | 3.3 | 0.018 | mitochondrial import inner membrane translocase subunit TIM9 | TIM9 |  | Mitochondrion inner membrane; Peripheral membrane protein; Intermembrane side (By similarity). | 30DS |
| KLMA_80017 | -13.0 | 5.0 | 0.002 | APC/C-CDH1 modulator 1 |  |  |  | S |
| KLMA_60156 | -12.9 | 4.3 | 0.000 | actin-related protein 2/3 complex subunit 4 | ARC19 | K05755 | Cytoplasm, cytoskeleton, actin patch. | S |
| KLMA_30042 | -12.9 | 4.3 | 0.007 | protein HAM1 | HAM1 | K01519 | Cytoplasm (By similarity). Nucleus (By similarity). | S |
| KLMA_60047 | -12.9 | 9.4 | 0.000 | uncharacterized protein YGR235C | MOS2 |  | Mitochondrion inner membrane; Multi-pass membrane protein. | 30DS |
| KLMA_60068 | -12.9 | 4.1 | 0.006 | mitochondral 37S ribosomal protein S27 | RSM27 |  | Mitochondrion. | 30X |
| KLMA_20608 | -12.8 | 4.2 | 0.000 | catabolite repression protein CAT5 | CAT5 | K06134 | Mitochondrion inner membrane. | S |
| KLMA_50563 | -12.8 | 3.3 | 0.000 | palmitoyltransferase ERF2 | ERF2 |  | Endoplasmic reticulum membrane; Multi-pass membrane protein (By similarity). | S |
| KLMA_70152 | -12.8 | 3.8 | 0.007 | vacuolar amino acid transporter 6 | AVT6 |  | Vacuole membrane; Multi-pass membrane protein. | S |
| KLMA_10466 | -12.8 | 4.7 | 0.000 | erv26 super family | SVP26 |  | Golgi apparatus, cis-Golgi network membrane; Multi-pass membrane protein. | S |
| KLMA_10735 | -12.8 | 3.8 | 0.000 | uracil phosphoribosyltransferase | FUR1 | K00761 |  | S |
| KLMA_40106 | -12.8 | 4.7 | 0.000 | uncharacterized protein YDR210W |  |  |  | 30X |
| KLMA_50627 | -12.8 | 3.1 | 0.000 | protein YTP1 | YTP1 |  | Membrane; Multi-pass membrane protein. | S |
| KLMA_60318 | -12.8 | 3.1 | 0.000 | transmembrane E3 ubiquitin-protein ligase 1 | TUL1 |  | Golgi apparatus membrane; Multi-pass membrane protein. | S |
| KLMA_50080 | -12.8 | 3.5 | 0.005 | putative carboxypeptidase YOL153C | CPS1 | K01293 | Vacuole membrane; Single-pass membrane protein. | S |
| KLMA_30665 | -12.8 | 2.7 | 0.021 | ACBP super family protein |  |  |  | S |
| KLMA_10064 | -12.7 | 3.8 | 0.016 | acetate non-utilizing protein 9 | ACN9 |  | Mitochondrion intermembrane space (By similarity). | S |
| KLMA_60473 | -12.7 | 2.6 | 0.008 | 54S ribosomal protein IMG1 | IMG1 |  | Mitochondrion. | S |
| KLMA_10588 | -12.7 | 4.4 | 0.000 | signal recognition particle SEC65 subunit | SEC65 | K03105 | Cytoplasm (By similarity). | S |
| KLMA_70126 | -12.7 | 3.1 | 0.002 | uncharacterized protein YJR080C | AIM24 |  | Mitochondrion (By similarity). | 30X |
| KLMA_70046 | -12.7 | 3.6 | 0.000 | elongation factor G | MEF1 | K02355 | Mitochondrion (By similarity). | S |
| KLMA_30439 | -12.6 | 3.3 | 0.000 | peptidyl-prolyl cis-trans isomerase D | CPR5 | K03768 | Endoplasmic reticulum lumen. | S |
| KLMA_30444 | -12.5 | 3.4 | 0.002 | vacuolar membrane protein YPL162C |  |  | Vacuole membrane; Multi-pass membrane protein. | S |
| KLMA_40044 | -12.5 | 4.7 | 0.001 | ethanolamine-phosphate cytidylyltransferase | ECT1 | K00967 | Cytoplasm. Nucleus. | 30X |
| KLMA_30367 | -12.5 | 4.8 | 0.000 | uncharacterized protein YLR257W |  |  | Cytoplasm. | S |
| KLMA_70027 | -12.5 | 3.9 | 0.005 | protein EMP47 | EMP47 |  | Golgi apparatus membrane; Single-pass type I membrane protein. Endoplasmic reticulum membrane; Single-pass type I membrane protein. | S |
| KLMA_60487 | -12.5 | 4.1 | 0.001 | vacuolar calcium ion transporter | VCX1 | K07300 | Vacuole membrane; Multi-pass membrane protein. | S |
| KLMA_70056 | -12.5 | 4.2 | 0.000 | tubulin beta chain | TUB2 | K07375 | Cytoplasm, cytoskeleton. | S |
| KLMA_10671 | -12.5 | 2.4 | 0.003 | uncharacterized protein YHL029C | OCA5 |  | Cytoplasm (By similarity). | S |
| KLMA_30243 | -12.5 | 2.7 | 0.003 | hydroxyacylglutathione hydrolase | GLO4 | K01069 | Mitochondrion matrix. | S |
| KLMA_30427 | -12.4 | 3.1 | 0.001 | HDA1 complex subunit 2 | HDA2 |  | Nucleus (By similarity). | S |
| KLMA_10629 | -12.4 | 3.7 | 0.044 | acyl carrier protein | ACP1 | K03955 | Mitochondrion. | S |
| KLMA_50039 | -12.4 | 3.7 | 0.000 | uncharacterized protein in HIS3 3'region |  |  |  | S |
| KLMA_20478 | -12.4 | 5.8 | 0.000 | centromere/microtubule-binding protein CBF5 | CBF5 | K11131 | Nucleus, nucleolus (By similarity). Chromosome, centromere (By similarity). Cytoplasm, cytoskeleton (By similarity). | S |
| KLMA_40041 | -12.4 | 3.7 | 0.000 | pre-mRNA-splicing factor 18 |  |  |  | S |
| KLMA_40183 | -12.4 | 4.6 | 0.000 | protein transport protein SEC22 | SEC22 | K08517 | Membrane; Single-pass type IV membrane protein (Potential). Endoplasmic reticulum membrane; Single-pass type IV membrane protein (Potential). Golgi apparatus membrane; Single-pass type IV membrane protein (Potential). | S |
| KLMA_50551 | -12.3 | 3.0 | 0.000 | COP9 signalosome complex subunit 9 | CSN9 |  | Cytoplasm (By similarity). Nucleus (By similarity). | S |
| KLMA_30473 | -12.3 | 2.3 | 0.000 | uncharacterized transporter YBR287W |  |  | Membrane; Multi-pass membrane protein (Potential). | S |
| KLMA_50348 | -12.3 | 3.2 | 0.000 | protein phosphatase methylesterase 1 | PPE1 | K13617 |  | S |
| KLMA_30357 | -12.3 | 3.5 | 0.001 | 3-ketoacyl-CoA reductase |  | K10251 | Endoplasmic reticulum membrane (By similarity); Single-pass membrane protein (Potential). | S |
| KLMA_60125 | -12.3 | 2.4 | 0.007 | succinate-semialdehyde dehydrogenase [NADP+] | gabD | K00135 |  | S |
| KLMA_50411 | -12.3 | 4.3 | 0.000 | hypothetical protein | GAS5 |  | Secreted, cell wall. Membrane; Lipid-anchor, GPI-anchor. | S |
| KLMA_30298 | -12.2 | 5.1 | 0.002 | ubiquitin-conjugating enzyme E2 13 | UBC13 | K10580 |  | S |
| KLMA_20546 | -12.2 | 4.1 | 0.001 | vacuolar protein sorting-associated protein 62 | VPS62 |  | Membrane; Single-pass membrane protein. | 30X |
| KLMA_70160 | -12.2 | 2.7 | 0.000 | mediator of RNA polymerase II transcription subunit 7 | MED7 | K15148 | Nucleus (By similarity). | S |
| KLMA_10805 | -12.2 | 2.1 | 0.000 | uncharacterized abhydrolase domain-containing protein YGR015C |  |  | Mitochondrion. | S |
| KLMA_20445 | -12.2 | 2.1 | 0.000 | adenylyltransferase and sulfurtransferase UBA4 | UBA4 | K11996 | Cytoplasm (By similarity). | S |
| KLMA_60399 | -12.2 | 2.2 | 0.003 | sporulation protein RMD5 | RMD5 |  | Cytoplasm. | S |
| KLMA_40157 | -12.2 | 3.2 | 0.000 | F-box protein HRT3 | HRT3 | K10295 |  | S |
| KLMA_40153 | -12.2 | 3.3 | 0.010 | thiol-specific monooxygenase | fmo1 |  |  | S |
| KLMA_40351 | -12.2 | 4.1 | 0.000 | hypothetical protein |  |  |  | S |
| KLMA_30653 | -12.2 | 3.5 | 0.003 | vacuolar protein sorting-associated protein 71 | VPS71 | K11663 | Nucleus. | S |
| KLMA_10581 | -12.2 | 3.5 | 0.002 | ubiquinone biosynthesis methyltransferase COQ5 | COQ5 | K06127 | Mitochondrion. | S |
| KLMA_80109 | -12.1 | 2.7 | 0.011 | frataxin homolog | YFH1 |  | Mitochondrion matrix. | S |
| KLMA_20319 | -12.1 | 2.5 | 0.004 | CTP-dependent diacylglycerol kinase 1 | DGK1 |  | Endoplasmic reticulum membrane; Multi-pass membrane protein. Golgi apparatus membrane; Multi-pass membrane protein. Nucleus membrane; Multi-pass membrane protein. | S |
| KLMA_20068 | -12.1 | 2.0 | 0.011 | coupling of ubiquitin conjugation to ER degradation protein 1 | CUE1 | K14022 | Endoplasmic reticulum membrane; Single-pass membrane protein (By similarity). | S |
| KLMA_30659 | -12.0 | 2.2 | 0.041 | probable aminopeptidase YDR415C |  | K01269 |  | S |
| KLMA_R514 | -12.0 | 2.0 | 0.001 | Glu-tRNA |  |  |  | 30X |
| KLMA_60410 | -12.0 | 4.2 | 0.000 | coatomer subunit delta | RET2 |  | Cytoplasm (By similarity). Golgi apparatus membrane; Peripheral membrane protein; Cytoplasmic side (By similarity). Cytoplasmic vesicle, COPI-coated vesicle membrane; Peripheral membrane protein; Cytoplasmic side (By similarity). | S |
| KLMA_60320 | -12.0 | 3.2 | 0.000 | ribose-phosphate pyrophosphokinase 3 | PRS3 | K00948 | Cytoplasm. | S |
| KLMA_10615 | -12.0 | 4.2 | 0.000 | 54S ribosomal protein L23 | rplM | K02871 |  | S |
| KLMA_30562 | -12.0 | 4.0 | 0.000 | UPF0010 protein YMR099C |  | K01792 |  | S |
| KLMA_60196 | -12.0 | 4.0 | 0.005 | ras-related protein RSR1 | RSR1 | K07974 | Cell membrane; Lipid-anchor; Cytoplasmic side (Potential). | S |
| KLMA_60203 | -12.0 | 2.5 | 0.000 | Golgi apparatus membrane protein TVP23 | TVP23 |  | Golgi apparatus membrane; Multi-pass membrane protein (By similarity). | S |
| KLMA_40118 | -12.0 | 4.4 | 0.000 | pre-mRNA-splicing factor SLT11 | SLT11 |  | Nucleus (By similarity). | S |
| KLMA_50117 | -11.9 | 2.6 | 0.001 | gamma-glutamyl phosphate reductase | PRO2 | K00147 |  | S |
| KLMA_20292 | -11.9 | 2.5 | 0.004 | uncharacterized protein YEL137C |  |  |  | S |
| KLMA_30383 | -11.9 | 3.2 | 0.002 | pH-response regulator protein palF/RIM8 | RIM8 |  |  | S |
| KLMA_30685 | -11.9 | 3.2 | 0.000 | uncharacterized protein YGL226W |  | K02263 |  | S |
| KLMA_20709 | -11.9 | 3.3 | 0.000 | autophagy-related protein 14 | ATG14 | K08335 | Preautophagosomal structure membrane; Peripheral membrane protein (By similarity). Vacuole membrane; Peripheral membrane protein (By similarity). | S |
| KLMA_30533 | -11.9 | 2.4 | 0.014 | 4,5-DOPA dioxygenase extradiol-like protein |  |  | Cytoplasm. Nucleus. | S |
| KLMA_20793 | -11.8 | 1.3 | 0.021 | MTP18 super family |  |  |  | S |
| KLMA_50088 | -11.8 | 5.0 | 0.000 | uncharacterized protein YBR262C | AIM5 |  | Mitochondrion inner membrane; Single-pass membrane protein (By similarity). | 30DS |
| KLMA_50023 | -11.8 | 6.4 | 0.000 | hypothetical protein |  |  |  | S |
| KLMA_10816 | -11.8 | 2.4 | 0.000 | ribonucleases P/MRP protein subunit POP7 | POP7 | K14526 | Nucleus. | S |
| KLMA_60115 | -11.8 | 3.0 | 0.000 | dehydrodolichyl diphosphate synthetase | RER2 | K11778 | Endoplasmic reticulum membrane; Peripheral membrane protein. | S |
| KLMA_40237 | -11.8 | 1.8 | 0.000 | putative glycoprotein endopeptidase KAE1 | gcp | K01409 | Cytoplasm (Potential). | 30X |
| KLMA_40059 | -11.8 | 2.3 | 0.000 | glucose-induced degradation protein 8 | GID8 |  | Nucleus. Cytoplasm. | S |
| KLMA_50406 | -11.8 | 5.4 | 0.007 | peroxiredoxin DOT5 | DOT5 | K03564 | Nucleus. Chromosome, telomere (Potential). | 30DS |
| KLMA_70278 | -11.7 | 4.1 | 0.001 | pyridoxamine 5'-phosphate oxidase homolog |  |  | Cytoplasm. Nucleus. | S |
| KLMA_80124 | -11.7 | 2.4 | 0.000 | RNA polymerase II holoenzyme cyclin-like subunit | SSN8 | K15161 | Nucleus (Probable). | S |
| KLMA_40343 | -11.7 | 2.8 | 0.000 | NADH-cytochrome b5 reductase 1 | CBR1 | K00326 | Endoplasmic reticulum membrane; Single-pass membrane protein (By similarity). Mitochondrion outer membrane; Single-pass membrane protein (By similarity). | S |
| KLMA_40360 | -11.7 | 2.3 | 0.000 | mitochondrial inner membrane magnesium transporter LPE10 | LPE10 |  | Mitochondrion inner membrane; Multi-pass membrane protein (By similarity). | S |
| KLMA_80369 | -11.7 | 6.4 | 0.000 | isoleucyl-tRNA synthetase | ILS1 | K01870 | Cytoplasm. | S |
| KLMA_20344 | -11.6 | 1.1 | 0.007 | mRNA 3'-end-processing protein YTH1 | YTH1 | K14404 | Nucleus (By similarity). | 30DS |
| KLMA_20605 | -11.6 | 2.9 | 0.000 | mitochondrial chaperone BCS1 |  | K08900 |  | S |
| KLMA_40301 | -11.6 | 1.0 | 0.001 | UPF0067 GAF domain-containing protein YKL069W |  | K08968 | Cytoplasm. Nucleus. | C |
| KLMA_10114 | -11.5 | 1.7 | 0.003 | phosphatidylinositol transfer protein PDR17 | PDR17 |  | Cytoplasm. | S |
| KLMA_40514 | -11.5 | 2.7 | 0.000 | hypothetical protein |  |  |  | S |
| KLMA_50619 | -11.5 | 3.4 | 0.001 | protein URE2 | URE2 | K00799 |  | S |
| KLMA_70331 | -11.5 | 2.0 | 0.002 | mitochondrial import inner membrane translocase subunit TIM12 | TIM12 |  | Mitochondrion inner membrane; Peripheral membrane protein. | 30DS |
| KLMA_10453 | -11.5 | 2.2 | 0.002 | conserved oligomeric Golgi complex subunit 7 | COG7 |  | Golgi apparatus membrane; Peripheral membrane protein (By similarity). | S |
| KLMA_40595 | -11.4 | 2.8 | 0.000 | phosphatidylinositol N-acetylglucosaminyltransferase subunit GPI19 | GPI19 | K03861 | Endoplasmic reticulum membrane; Multi-pass membrane protein (By similarity). | S |
| KLMA_20705 | -11.4 | 2.1 | 0.002 | hypothetical protein |  |  |  | S |
| KLMA_70162 | -11.4 | 1.7 | 0.005 | putative prephenate dehydratase | PHA2 | K14170 | Cytoplasm. | S |
| KLMA_60314 | -11.4 | 2.6 | 0.000 | putative GTP-binding protein YLF2 | engD | K06942 |  | S |
| KLMA_10482 | -11.4 | 1.9 | 0.009 | serine/threonine-protein kinase BUD32 | BUD32 | K08851 | Cytoplasm (By similarity). Nucleus (By similarity). Chromosome, telomere (By similarity). | S |
| KLMA_30591 | -11.3 | 2.3 | 0.006 | bolA-like protein 3 | AIM1 |  |  | S |
| KLMA_10812 | -11.3 | 4.5 | 0.000 | nuclear protein localization protein 4 | NPL4 | K14015 | Cytoplasm, perinuclear region (By similarity). Endoplasmic reticulum membrane; Peripheral membrane protein; Cytoplasmic side (By similarity). Nucleus membrane; Peripheral membrane protein; Cytoplasmic side (By similarity). | S |
| KLMA_50428 | -11.3 | 3.6 | 0.000 | cell cycle protein kinase DBF2 | DBF2 | K06684 |  | S |
| KLMA_50302 | -11.3 | 3.2 | 0.033 | eukaryotic translation initiation factor 3 subunit J | HCR1 | K03245 | Cytoplasm (By similarity). | S |
| KLMA_40289 | -11.2 | 4.0 | 0.000 | AP-1 complex subunit sigma-1 | APS1 |  | Golgi apparatus. Cytoplasmic vesicle membrane; Peripheral membrane protein; Cytoplasmic side. Membrane, clathrin-coated pit. | S |
| KLMA_70004 | -11.2 | 2.0 | 0.000 | conserved hypothetical protein |  |  |  | S |
| KLMA_60422 | -11.2 | 2.6 | 0.016 | prefoldin subunit 4 | GIM3 | K09550 |  | S |
| KLMA_60319 | -11.2 | 2.8 | 0.000 | putative uncharacterized hydrolase YKL033W-A |  |  |  | S |
| KLMA_50412 | -11.2 | 2.3 | 0.005 | 1,2-dihydroxy-3-keto-5-methylthiopentene dioxygenase; Acireductone dioxygenase | ADI1 | K08967 | Cytoplasm. Nucleus. | S |
| KLMA_60279 | -11.2 | 2.1 | 0.000 | 3'(2')5'-bisphosphate nucleotidase | MET22 | K01082 | Cytoplasm. Nucleus. | S |
| KLMA_60078 | -11.2 | 2.4 | 0.002 | phosphoserine phosphatase | SER2 | K01079 |  | S |
| KLMA_20279 | -11.2 | 1.6 | 0.009 | uncharacterized membrane protein YBR220C |  |  | Membrane; Multi-pass membrane protein. | S |
| KLMA_30285 | -11.2 | 2.4 | 0.006 | trans-aconitate 3-methyltransferase | TMT1 |  | Cytoplasm (By similarity). | S |
| KLMA_60096 | -11.1 | 3.2 | 0.000 | exodeoxyribonuclease 1 | EXO1 | K10746 | Nucleus (Potential). | S |
| KLMA_20376 | -11.1 | 3.6 | 0.007 | eukaryotic translation initiation factor 3 subunit I | TIF34 | K03246 | Cytoplasm (By similarity). | S |
| KLMA_10265 | -11.1 | 3.1 | 0.001 | mitochondrial distribution and morphology protein 12 | MDM12 |  | Mitochondrion outer membrane; Peripheral membrane protein; Cytoplasmic side (By similarity). Endoplasmic reticulum membrane; Peripheral membrane protein; Cytoplasmic side (By similarity). | S |
| KLMA_20526 | -11.1 | 2.6 | 0.019 | F-actin-capping protein subunit beta | CAP2 | K10365 | Cytoplasm, cytoskeleton (By similarity). Nucleus (By similarity). | S |
| KLMA_20474 | -11.1 | 2.7 | 0.000 | autophagy-related protein 18 | ATG18 |  | Preautophagosomal structure membrane; Peripheral membrane protein (By similarity). Vacuole membrane; Peripheral membrane protein (By similarity). Endosome membrane; Peripheral membrane protein (By similarity). | S |
| KLMA_40196 | -11.1 | 2.2 | 0.000 | protein PTI1 | PTI1 | K14407 | Nucleus. | S |
| KLMA_40598 | -11.0 | 1.5 | 0.001 | transcription initiation factor TFIID subunit 11 | TAF11 | K03135 | Nucleus. | S |
| KLMA_50028 | -11.0 | 2.2 | 0.000 | putative 2-hydroxyacid dehydrogenase YPL113C |  |  |  | S |
| KLMA_50081 | -11.0 | 4.4 | 0.000 | RNA polymerase II-associated protein 1 | PAF1 | K15174 | Nucleus, nucleoplasm. | S |
| KLMA_40444 | -11.0 | 6.7 | 0.002 | FK506-binding protein 3 | FPR3 | K14826 | Nucleus, nucleolus (By similarity). | S |
| KLMA_20055 | -11.0 | 0.5 | 0.037 | hypothetical protein |  |  |  | S |
| KLMA_40078 | -11.0 | 0.5 | 0.038 | v-type ATPase assembly factor PKR1 |  |  |  | S |
| KLMA_30378 | -11.0 | 3.1 | 0.000 | dolichyl-diphosphooligosaccharide--protein glycosyltransferase subunit STT3 | STT3 | K07151 | Endoplasmic reticulum membrane; Multi-pass membrane protein. | S |
| KLMA_30350 | -11.0 | 2.6 | 0.003 | hsp70/Hsp90 co-chaperone CNS1 | CNS1 |  | Cytoplasm. | S |
| KLMA_R704 | -11.0 | 2.3 | 0.021 | Val-tRNA |  |  |  | S |
| KLMA_20287 | -10.9 | 2.5 | 0.000 | hypothetical protein |  |  | Mitochondrion (Potential). | S |
| KLMA_20353 | -10.9 | 1.4 | 0.001 | nucleotide exchange factor SIL1 | SIL1 | K14001 | Endoplasmic reticulum lumen (By similarity). | S |
| KLMA_80270 | -10.9 | 1.8 | 0.008 | 37S ribosomal protein RSM28 | RSM28 |  | Mitochondrion. | S |
| KLMA_50346 | -10.9 | 0.7 | 0.034 | UPF0676 protein C1494.01 |  |  | Cytoplasm. Nucleus. | S |
| KLMA_80259 | -10.9 | 2.1 | 0.001 | DASH complex subunit ASK1 | ASK1 | K11566 | Nucleus (By similarity). Cytoplasm, cytoskeleton, spindle (By similarity). Chromosome, centromere, kinetochore (By similarity). | S |
| KLMA_40267 | -10.9 | 2.8 | 0.001 | initiation-specific alpha-1 | OCH1 | K05528 | Endoplasmic reticulum membrane; Single-pass type II membrane protein. Golgi apparatus membrane; Single-pass type II membrane protein. | S |
| KLMA_40105 | -10.9 | 2.2 | 0.008 | uncharacterized glycosyl hydrolase YBR056W |  | K01210 |  | S |
| KLMA_80331 | -10.9 | 1.6 | 0.001 | carrier protein YMC1 | YMC1 | K15109 | Mitochondrion inner membrane; Multi-pass membrane protein (Potential). | S |
| KLMA_20804 | -10.9 | 1.6 | 0.014 | gatB_Yqey super family | AIM41 | K09117 | Mitochondrion (By similarity). | S |
| KLMA_40517 | -10.9 | 1.4 | 0.012 | uncharacterized protein YML079W |  | K09705 |  | S |
| KLMA_20629 | -10.8 | 2.2 | 0.009 | 6,7-dimethyl-8-ribityllumazine synthase | RIB4 | K00794 |  | S |
| KLMA_40167 | -10.8 | 2.9 | 0.002 | tRNA-splicing endonuclease subunit SEN2 | SEN2 | K15322 |  | S |
| KLMA_60382 | -10.7 | 1.3 | 0.011 | probable gluconokinase |  | K00851 | Cytoplasm. | 30DS |
| KLMA_10626 | -10.7 | 2.3 | 0.001 | threonyl-tRNA synthetase | MST1 | K01868 | Mitochondrion matrix. | S |
| KLMA_20711 | -10.7 | 0.5 | 0.043 | uncharacterized protein YPR085C | ASA1 |  | Nucleus (By similarity). | S |
| KLMA_50246 | -10.7 | 3.8 | 0.007 | ribosomal N-lysine methyltransferase 3 | RKM3 |  | Nucleus. | S |
| KLMA_10224 | -10.7 | 0.5 | 0.006 | translation initiation factor eIF-2B subunit beta | GCD7 | K03754 |  | S |
| KLMA_20759 | -10.7 | 1.4 | 0.005 | protein IBD2 | IBD2 |  | Cytoplasm, cytoskeleton, spindle pole (By similarity). | S |
| KLMA_10591 | -10.6 | 3.8 | 0.014 | protein TMA23 | TMA23 | K14796 | Nucleus, nucleolus. | S |
| KLMA_10138 | -10.6 | 1.6 | 0.009 | vacuolar protein sorting-associated protein 75 | VPS75 |  | Nucleus. | S |
| KLMA_10675 | -10.6 | 1.4 | 0.014 | cytochrome c oxidase assembly protein COX19 | COX19 |  | Cytoplasm (By similarity). Mitochondrion intermembrane space (By similarity). | S |
| KLMA_50525 | -10.6 | 0.1 | 0.046 | transcription initiation factor TFIID subunit 10 | TAF10 | K03134 | Nucleus. | S |
| KLMA_40349 | -10.5 | 2.1 | 0.001 | ER membrane protein complex subunit 5 | EMC5 |  | Endoplasmic reticulum membrane; Multi-pass membrane protein. | S |
| KLMA_20267 | -10.5 | 5.1 | 0.000 | nuclear polyadenylated RNA-binding protein NAB2 | NAB2 |  | Nucleus. | S |
| KLMA_40306 | -10.4 | 1.6 | 0.017 | ISWI one complex protein 4 | IOC4 |  | Nucleus. | S |
| KLMA_20552 | -10.4 | 3.5 | 0.045 | arginine N-methyltransferase 2 | RMT2 | K00599 | Cytoplasm (By similarity). Nucleus (By similarity). | S |
| KLMA_70060 | -10.4 | 1.2 | 0.036 | uncharacterized protein YLR063W |  |  | Cytoplasm. | S |
| KLMA_80181 | -10.4 | 3.8 | 0.003 | zuotin | ZUO1 | K09522 | Cytoplasm. | S |
| KLMA_20164 | -10.4 | 7.8 | 0.000 | hypothetical protein |  |  |  | S |
| KLMA_20548 | -10.4 | 2.4 | 0.012 | kpsF-like protein |  |  |  | S |
| KLMA_10166 | -10.4 | 1.3 | 0.001 | vacuolar protein sorting-associated protein 52 | VPS52 |  | Golgi apparatus, trans-Golgi network membrane; Peripheral membrane protein. Endosome membrane; Peripheral membrane protein. Cytoplasm, cytoskeleton. | S |
| KLMA_50530 | -10.4 | 2.7 | 0.004 | peroxisomal coenzyme A diphosphatase 1 | PCD1 |  | Peroxisome. | S |
| KLMA_70416 | -10.4 | 1.3 | 0.012 | uncharacterized protein YER128W | VFA1 |  | Cytoplasm. Endosome. | S |
| KLMA_80117 | -10.4 | 1.2 | 0.006 | probable mannosyltransferase KTR5 | KTR5 | K03854 | Membrane; Single-pass type II membrane protein (Probable). | S |
| KLMA_70078 | -10.3 | 1.9 | 0.000 | central kinetochore subunit CTF3 | CTF3 | K11501 | Nucleus. Chromosome, centromere, kinetochore. | S |
| KLMA_40056 | -10.3 | 3.7 | 0.010 | GTP-binding protein CIN4 | CIN4 | K07943 |  | S |
| KLMA_50618 | -10.3 | 2.5 | 0.004 | J protein JJJ1 | JJJ1 | K09506 | Nucleus (Potential). | S |
| KLMA_30666 | -10.3 | 1.0 | 0.029 | DNA polymerase eta | RAD30 | K03509 | Nucleus. | S |
| KLMA_80379 | -10.3 | 1.3 | 0.003 | dolichyl-P-Man:Man(5)GlcNAc(2)-PP-dolichyl mannosyltransferase | ALG3 | K03845 | Endoplasmic reticulum membrane; Multi-pass membrane protein (By similarity). | S |
| KLMA_70277 | -10.2 | 1.8 | 0.006 | PWWP domain-containing protein YLR455W |  |  | Nucleus. | S |
| KLMA_40531 | -10.2 | 6.8 | 0.000 | uncharacterized protein YKL187C |  |  | Mitochondrion. | S |
| KLMA_20309 | -10.2 | 3.6 | 0.000 | CCR4-associated factor 16 | CAF16 | K12608 | Cytoplasm. Nucleus. | S |
| KLMA_10488 | -10.2 | 4.0 | 0.001 | GTP cyclohydrolase 1 | FOL2 | K01495 |  | 30X |
| KLMA_60360 | -10.2 | 1.9 | 0.000 | probable endonuclease YGL085W | LCL3 |  | Mitochondrion. Membrane; Single-pass membrane protein (By similarity). | S |
| KLMA_40449 | -10.2 | 1.0 | 0.040 | epsin-3 | ENT3 |  | Cytoplasm. Golgi apparatus, trans-Golgi network membrane; Peripheral membrane protein. Cytoplasmic vesicle, clathrin-coated vesicle membrane; Peripheral membrane protein. | S |
| KLMA_20772 | -10.1 | 0.2 | 0.001 | hypothetical protein |  |  |  | S |
| KLMA_70207 | -10.1 | 0.1 | 0.011 | twinfilin-1 | TWF1 |  | Cytoplasm, cytoskeleton (By similarity). | S |
| KLMA_20727 | -10.1 | 1.1 | 0.036 | probable glutamine amidotransferase DUG3 | DUG3 | K07008 | Cytoplasm. | S |
| KLMA_60400 | -10.1 | 2.6 | 0.048 | SAGA-associated factor 11 | SGF11 | K11363 | Nucleus (By similarity). | S |
| KLMA_60247 | -10.1 | 2.1 | 0.003 | cytochrome c oxidase-assembly factor COX23 | COX23 |  | Mitochondrion intermembrane space (By similarity). | S |
| KLMA_20281 | -10.1 | 2.7 | 0.000 | FIT family protein SCS3 | SCS3 |  | Membrane; Multi-pass membrane protein (Potential). | S |
| KLMA_70063 | -10.0 | 2.5 | 0.005 | peroxisomal membrane protein import receptor PEX19 | PEX19 | K13337 | Cytoplasm (By similarity). Peroxisome membrane; Lipid-anchor; Cytoplasmic side (By similarity). Endoplasmic reticulum membrane (By similarity). | S |
| KLMA_20747 | -10.0 | 4.9 | 0.021 | nucleolar protein 13 | NOP13 |  | Nucleus, nucleolus. | S |
| KLMA_10095 | -10.0 | 1.1 | 0.005 | boron transporter 1 | BOR1 |  | Cell membrane; Multi-pass membrane protein. Vacuole membrane; Multi-pass membrane protein. | S |
| KLMA_30500 | -9.9 | 2.4 | 0.001 | forkhead transcription factor HCM1 | HCM1 | K09413 | Cytoplasm. Nucleus. | S |
| KLMA_60288 | -9.9 | 2.8 | 0.000 | mannan endo-1 | DFG5 | K08257 | Cell membrane; Lipid-anchor, GPI-anchor. | S |
| KLMA_20532 | -9.9 | 1.4 | 0.000 | peroxisomal targeting signal 2 receptor | PEX7 | K13341 | Cytoplasm. Peroxisome. | S |
| KLMA_50567 | -9.9 | 2.0 | 0.007 | T-complex protein 1 subunit zeta | CCT6 | K09498 | Cytoplasm. | S |
| KLMA_10725 | -9.8 | 0.4 | 0.021 | DNA repair and recombination protein RAD26 | RAD26 | K10841 | Nucleus (Probable). | S |
| KLMA_60533 | -9.8 | 1.8 | 0.037 | chromosome segregation in meiosis protein 2 | CSM2 |  | Cytoplasm (By similarity). Nucleus (By similarity). | S |
| KLMA_30523 | -9.8 | 1.6 | 0.007 | riboflavin kinase | FMN1 | K00861 |  | S |
| KLMA_50392 | -9.8 | 1.0 | 0.010 | protein VAB2 | VAB2 |  | Cytoplasmic vesicle (By similarity). Vacuole (By similarity). Cytoplasm (By similarity). | S |
| KLMA_50510 | -9.8 | 2.2 | 0.001 | pre-mRNA-splicing factor CWC15 | CWC15 | K12863 | Nucleus (Probable). | S |
| KLMA_80269 | -9.8 | 0.9 | 0.025 | tRNA 2'-phosphotransferase | TPT1 | K10669 |  | S |
| KLMA_80351 | -9.8 | 2.9 | 0.001 | hypothetical protein | AIM11 |  | Membrane; Multi-pass membrane protein (Potential). | S |
| KLMA_80041 | -9.7 | 0.7 | 0.001 | exosome complex exonuclease RRP6 | RRP6 | K12591 | Nucleus, nucleolus. | S |
| KLMA_80358 | -9.7 | 1.6 | 0.018 | mitochondrial peroxiredoxin PRX1 | PRX1 | K03386 | Mitochondrion. | S |
| KLMA_10627 | -9.7 | 1.1 | 0.028 | uncharacterized protein YNL040W |  | K07050 |  | S |
| KLMA_70128 | -9.6 | 1.1 | 0.001 | uncharacterized protein YBL107C |  |  |  | S |
| KLMA_40241 | -9.6 | 1.6 | 0.001 | uncharacterized GTP-binding protein YDR336W |  |  |  | S |
| KLMA_30180 | -9.5 | 2.0 | 0.023 | clathrin light chain | CLC1 |  | Cytoplasmic vesicle membrane; Peripheral membrane protein; Cytoplasmic side. Membrane, coated pit; Peripheral membrane protein; Cytoplasmic side. | S |
| KLMA_80020 | -9.5 | 1.7 | 0.004 | karyogamy protein KAR9 | KAR9 |  | Nucleus. Cytoplasm, cytoskeleton. | S |
| KLMA_30255 | -9.5 | 4.4 | 0.008 | 20S-pre-rRNA D-site endonuclease NOB1 | NOB1 | K11883 | Cytoplasm. Nucleus, nucleolus. Endoplasmic reticulum. | S |
| KLMA_10282 | -9.5 | 3.3 | 0.000 | pre-rRNA-processing protein PNO1 | PNO1 | K11884 | Cytoplasm. Nucleus, nucleolus (By similarity). | S |
| KLMA_10228 | -9.5 | 2.3 | 0.002 | U1 small nuclear ribonucleoprotein C homolog | YHC1 |  | Nucleus. | S |
| KLMA_10722 | -9.4 | 3.5 | 0.005 | probable E3 ubiquitin-protein ligase HUL4 | HUL4 | K12232 | Nucleus (Probable). | S |
| KLMA_80043 | -9.4 | 0.8 | 0.046 | tRNA A64-2'-O-ribosylphosphate transferase | RIT1 | K15463 |  | S |
| KLMA_30203 | -9.4 | 2.1 | 0.004 | alcohol O-acetyltransferase 1 | ATF1 | K00664 | Membrane; Peripheral membrane protein. | S |
| KLMA_10342 | -9.4 | 1.1 | 0.001 | dihydrosphingosine 1-phosphate phosphatase LCB3 | LCB3 | K04716 | Endoplasmic reticulum membrane; Multi-pass membrane protein. | S |
| KLMA_50607 | -9.3 | 0.7 | 0.006 | DNA-dependent ATPase MGS1 | MGS1 | K07478 | Nucleus (Potential). | S |
| KLMA_50561 | -9.3 | 2.5 | 0.001 | protein ATC1/LIC4 | ATC1 |  | Cytoplasm (By similarity). Nucleus (By similarity). | S |
| KLMA_30159 | -9.3 | 2.5 | 0.036 | 54S ribosomal protein L49 | MRPL49 |  | Mitochondrion. | S |
| KLMA_70173 | -9.3 | 1.1 | 0.028 | ran-specific GTPase-activating protein 2 | YRB2 | K15304 | Nucleus. | S |
| KLMA_40433 | -9.3 | 0.6 | 0.001 | autophagy-related protein 7 | ATG7 | K08337 | Cytoplasm (By similarity). | S |
| KLMA_10162 | -9.2 | 1.1 | 0.012 | vacuolar protein-sorting-associated protein 60 | VPS60 | K12198 | Endosome membrane; Peripheral membrane protein. Vacuole membrane; Peripheral membrane protein. | S |
| KLMA_10202 | -9.2 | 1.7 | 0.003 | uncharacterized protein C12G12.12 |  |  | Membrane; Multi-pass membrane protein (Potential). | S |
| KLMA_30504 | -9.2 | 5.5 | 0.000 | putative pyridoxal kinase BUD17 | BUD17 | K00868 | Cytoplasm. Nucleus. | S |
| KLMA_80106 | -9.2 | 1.8 | 0.010 | solute carrier family 25 member 38 homolog |  | K15118 | Mitochondrion inner membrane; Multi-pass membrane protein (By similarity). | S |
| KLMA_40236 | -9.1 | 1.4 | 0.029 | DASH complex subunit SPC34 | SPC34 | K11573 | Nucleus (By similarity). Cytoplasm, cytoskeleton, spindle (By similarity). Chromosome, centromere, kinetochore (By similarity). | S |
| KLMA_30358 | -9.1 | 2.2 | 0.014 | cell division control protein 28 | CDC28 | K04563 |  | S |
| KLMA_70444 | -9.1 | 6.1 | 0.000 | citrate synthase 3 | CIT3 | K01647 |  | 30DS |
| KLMA_70264 | -9.0 | 1.5 | 0.014 | origin recognition complex subunit 4 | ORC4 | K02606 | Nucleus. | S |
| KLMA_10066 | -9.0 | 1.1 | 0.031 | CAP_GLY super family | BIK1 |  | Cytoplasm, cytoskeleton, spindle pole body. Cytoplasm, cytoskeleton, spindle. Cytoplasm. | S |
| KLMA_60407 | -9.0 | 2.5 | 0.002 | proteasome component PRE4 | PRE4 | K02736 | Cytoplasm. Nucleus. | S |
| KLMA_10429 | -8.9 | 4.2 | 0.000 | NADH dehydrogenase |  | K03885 |  | S |
| KLMA_10316 | -8.9 | 0.5 | 0.042 | uroporphyrinogen-III synthase | HEM4 | K01719 |  | S |
| KLMA_50408 | -8.9 | 8.2 | 0.000 | protein SLG1 |  | K11244 |  | S |
| KLMA_20823 | -8.9 | 4.2 | 0.000 | uncharacterized transporter YBR287W |  |  | Membrane; Multi-pass membrane protein (Potential). | S |
| KLMA_R706 | -8.9 | 0.2 | 0.007 | Ile-tRNA |  |  |  | S |
| KLMA_60142 | -8.8 | 2.6 | 0.030 | N-glycosylation protein EOS1 | EOS1 |  | Endoplasmic reticulum membrane; Multi-pass membrane protein. | S |
| KLMA_60166 | -8.7 | 0.6 | 0.013 | sulfiredoxin | SRX1 | K12260 | Cytoplasm. Nucleus. | S |
| KLMA_10302 | -8.7 | 3.7 | 0.041 | UPF0399 protein YOR287C | RRP36 | K14795 | Nucleus, nucleolus (By similarity). | S |
| KLMA_10688 | -8.6 | 0.0 | 0.037 | protein BIG1 | BIG1 |  | Endoplasmic reticulum membrane; Single-pass type I membrane protein (By similarity). | S |
| KLMA_20655 | -8.6 | 1.4 | 0.027 | polynucleotide 3'-phosphatase | TPP1 | K08075 | Nucleus (Probable). | S |
| KLMA_50107 | -8.6 | 0.1 | 0.006 | GLC7-interacting protein 4 | GIP4 |  | Cytoplasm (By similarity). | S |
| KLMA_20560 | -8.5 | 1.8 | 0.045 | vacuolar protein sorting-associated protein 29 | VPS29 | K07095 |  | S |
| KLMA_40325 | -8.3 | 5.0 | 0.000 | hypothetical protein |  |  |  | S |
| KLMA_40623 | -8.3 | 2.2 | 0.023 | delta(12) fatty acid desaturase |  | K10256 | Membrane; Multi-pass membrane protein (Potential). | S |
| KLMA_50429 | -8.3 | 2.4 | 0.001 | CWF19-like protein DRN1 | DRN1 |  | Nucleus. | S |
| KLMA_40483 | -8.3 | 0.9 | 0.021 | uncharacterized protein YHR202W |  |  | Vacuole. | S |
| KLMA_40588 | -8.3 | 0.9 | 0.007 | histone-lysine N-methyltransferase | DOT1 | K11427 | Nucleus (By similarity). | S |
| KLMA_10383 | -8.2 | 6.9 | 0.001 | histone-lysine N-methyltransferase | SET2 | K11423 | Nucleus (By similarity). Chromosome (By similarity). | S |
| KLMA_70158 | -8.2 | 2.2 | 0.005 | polyadenylation factor subunit 2 | PFS2 | K15542 | Nucleus (By similarity). | S |
| KLMA_50602 | -8.1 | 3.2 | 0.010 | PAPA-1 super family conserved domain |  | K11676 |  | S |
| KLMA_10201 | -8.1 | 0.4 | 0.036 | e3 ubiquitin-protein ligase complex SLX5-SLX8 subunit SLX5 | SLX5 |  | Nucleus, nucleolus. | S |
| KLMA_60289 | -8.0 | 4.0 | 0.021 | protein BCH1 | BUD7 |  | Golgi apparatus, trans-Golgi network membrane; Peripheral membrane protein. | S |
| KLMA_40430 | -8.0 | 2.1 | 0.026 | ATP-dependent RNA helicase DBP8 | DBP8 | K14778 | Nucleus, nucleolus (By similarity). | S |
| KLMA_10456 | -7.9 | 5.9 | 0.000 | tRNA-splicing endonuclease subunit SEN34 | SEN34 | K15323 | Nucleus. Endomembrane system; Peripheral membrane protein. Mitochondrion outer membrane; Peripheral membrane protein; Cytoplasmic side. | S |
| KLMA_80312 | -7.8 | 5.1 | 0.039 | glycyl-tRNA synthetase 1 | GRS1 | K01880 | Isoform Cytoplasmic: Cytoplasm.Isoform Mitochondrial: Mitochondrion matrix. | S |
| KLMA_40271 | -7.8 | 4.6 | 0.035 | zinc finger protein |  |  |  | S |
| KLMA_30468 | -7.8 | 3.4 | 0.005 | protoheme IX farnesyltransferase | COX10 | K02257 | Mitochondrion membrane; Multi-pass membrane protein (By similarity). | S |
| KLMA_10035 | -7.8 | 3.8 | 0.035 | 5',5'''-P-1,P-4-tetraphosphate phosphorylase 2 | APA2 | K00988 |  | 30X |
| KLMA_70129 | -7.8 | 1.7 | 0.000 | protein SNI2 | SRO77 |  |  | S |
| KLMA_20627 | -7.8 | 1.2 | 0.003 | peroxisomal biogenesis factor 6 | PEX6 | K13339 |  | S |
| KLMA_20406 | -7.6 | 5.8 | 0.000 | nucleoporin SEH1 | SEH1 | K14299 | Nucleus, nuclear pore complex. Cell membrane; Peripheral membrane protein; Cytoplasmic side. Vacuole membrane; Peripheral membrane protein. Cell membrane; Peripheral membrane protein; Nucleoplasmic side. | S |
| KLMA_10828 | -7.6 | 4.2 | 0.008 | hypothetical protein |  |  |  | S |
| KLMA_60114 | -7.6 | 1.6 | 0.001 | hexaprenyl pyrophosphate synthetase | COQ1 | K05355 | Mitochondrion inner membrane; Peripheral membrane protein; Matrix side. | S |
| KLMA_10832 | -7.6 | 6.9 | 0.000 | dihydroorotate dehydrogenase | URA1 | K00226 | Cytoplasm. | S |
| KLMA_40299 | -7.4 | 1.7 | 0.001 | transposon Ty2-LR1 Gag-Pol polyprotein | TY2B-LR1 |  | Cytoplasm. Nucleus (By similarity). | S |
| KLMA_80279 | -7.4 | 3.5 | 0.003 | ribonucleases P/MRP protein subunit POP3 |  | K14522 |  | S |
| KLMA_50256 | -7.3 | 1.3 | 0.017 | cyclin-dependent kinase inhibitor FAR1 | FAR1 | K06652 |  | 30DS |
| KLMA_80142 | -7.3 | 4.8 | 0.000 | carbonic anhydrase | NCE103 | K01673 | Cytoplasm. Nucleus. | S |
| KLMA_30654 | -7.3 | 6.0 | 0.001 | carnitine O-acetyltransferase | CAT2 | K00624 | Isoform Mitochondrial: Mitochondrion inner membrane; Peripheral membrane protein; Matrix side.Isoform Peroxisomal: Peroxisome. | S |
| KLMA_70305 | -7.3 | 7.2 | 0.013 | copper transport protein CTR1 | CTR1 |  | Membrane; Multi-pass membrane protein. | S |
| KLMA_60262 | -7.1 | 3.0 | 0.008 | UPF0195 protein YHR122W |  |  |  | S |
| KLMA_40578 | -7.1 | 5.2 | 0.000 | 54S ribosomal protein YmL6 | YML6 |  | Mitochondrion. | S |
| KLMA_50155 | -7.0 | 7.3 | 0.001 | actin cytoskeleton-regulatory complex protein SLA1 | SLA1 |  | Cell membrane; Peripheral membrane protein; Cytoplasmic side (By similarity). Endosome membrane; Peripheral membrane protein; Cytoplasmic side (By similarity). Cytoplasm, cytoskeleton, actin patch (By similarity). | S |
| KLMA_40273 | -6.9 | 6.8 | 0.000 | nuclear pore complex subunit | NUP49 | K14307 | Nucleus, nuclear pore complex. Nucleus membrane; Peripheral membrane protein; Cytoplasmic side. Nucleus membrane; Peripheral membrane protein; Nucleoplasmic side. | S |
| KLMA_30102 | -6.9 | 3.5 | 0.003 | uncharacterized protein YKL128C | PMU1 |  | Cytoplasm. Nucleus. | 30X |
| KLMA_20791 | -6.9 | 5.5 | 0.003 | iron sulfur cluster assembly protein 1 | ISU1 | K04488 | Mitochondrion matrix (By similarity). | S |
| KLMA_50036 | -6.8 | 3.6 | 0.003 | isopentenyl-diphosphate Delta-isomerase | IDI1 | K01823 | Cytoplasm. | S |
| KLMA_10512 | -6.8 | 7.1 | 0.003 | uncharacterized mitochondrial membrane protein FMP10 | FMP10 |  | Mitochondrion membrane; Multi-pass membrane protein (Potential). | S |
| KLMA_50037 | -6.8 | 3.0 | 0.006 | rRNA methyltransferase | MRM1 | K15507 | Mitochondrion. | S |
| KLMA_20540 | -6.8 | 7.0 | 0.003 | proteasome component Y13 | PRE9 | K02728 | Cytoplasm. Nucleus. | S |
| KLMA_30552 | -6.7 | 4.9 | 0.000 | eukaryotic translation initiation factor 3 subunit A | TIF32 | K03254 | Cytoplasm (By similarity). | S |
| KLMA_20648 | -6.7 | 1.3 | 0.030 | uncharacterized protein YMR160W |  |  |  | S |
| KLMA_20820 | -6.7 | 2.4 | 0.032 | hypothetical protein |  |  |  | 30DS |
| KLMA_40220 | -6.6 | 11.3 | 0.000 | alcohol dehydrogenase 2 | ADH2 | K13953 | Cytoplasm. | 30X |
| KLMA_10829 | -6.6 | 5.6 | 0.001 | hypothetical protein |  |  |  | 30DS |
| KLMA_80069 | -6.5 | 4.7 | 0.001 | alpha-agglutinin | SAG1 |  | Secreted, cell wall. Membrane; Lipid-anchor, GPI-anchor. | S |
| KLMA_10378 | -6.5 | 2.5 | 0.002 | protein CASP | COY1 | K09313 | Golgi apparatus membrane; Single-pass type IV membrane protein. | S |
| KLMA_60085 | -6.4 | 5.8 | 0.001 | uncharacterized kinase YGR205W | TDA10 |  | Cytoplasm. Nucleus. | S |
| KLMA_40082 | -6.2 | 5.5 | 0.002 | protein ASI1 | ASI1 |  | Nucleus inner membrane; Multi-pass membrane protein. | S |
| KLMA_50552 | -6.2 | 1.0 | 0.034 | uncharacterized protein YDR179W-A |  |  |  | S |
| KLMA_50218 | -6.2 | 11.0 | 0.009 | protein MBR1 |  |  |  | S |
| KLMA_40453 | -6.2 | 3.2 | 0.001 | tRNA-dihydrouridine synthase 4 | DUS4 | K05545 |  | S |
| KLMA_60111 | -6.2 | 1.2 | 0.005 | sorting assembly machinery 35 kDa subunit | SAM35 |  | Mitochondrion outer membrane. | S |
| KLMA_40624 | -6.1 | 9.6 | 0.008 | alcohol dehydrogenase | adh | K00001 |  | S |
| KLMA_10518 | -6.1 | 12.9 | 0.000 | inulinase | INU1 | K01193 | Secreted. | 30DS |
| KLMA_30392 | -6.0 | 1.7 | 0.003 | UPF0657 nucleolar protein YBR141C |  |  | Nucleus, nucleolus. | S |
| KLMA_70303 | -6.0 | 4.9 | 0.007 | probable 6-phosphofructo-2-kinase/fructose-2,6-biphosphatase |  |  |  | 30DS |
| KLMA_80044 | -5.8 | 1.2 | 0.029 | hypothetical protein | AEP2 |  | Mitochondrion (By similarity). | S |
| KLMA_30136 | -5.8 | 3.3 | 0.002 | putative succinate-semialdehyde dehydrogenase C1002.12c [NADP+] | gabD |  |  | S |
| KLMA_80119 | -5.7 | 11.8 | 0.000 | histone H3 | HHT1 | K11253 | Nucleus (By similarity). Chromosome (By similarity). | 30DS |
| KLMA_30398 | -5.6 | 8.5 | 0.001 | 2-deoxyglucose-6-phosphate phosphatase 2 | DOG2 | K01111 |  | 30DS |
| KLMA_60412 | -5.6 | 10.7 | 0.000 | hexokinase | RAG5 | K00844 |  | C |
| KLMA_50514 | -5.6 | 2.4 | 0.025 | putative ribonuclease YLR143W |  |  | Cytoplasm. | S |
| KLMA_60167 | -5.6 | 12.1 | 0.000 | malate dehydrogenase | MDH1 | K00026 | Mitochondrion matrix. | 30DS |
| KLMA_70178 | -5.5 | 12.0 | 0.000 | uncharacterized protein YIL057C | RGI1 |  | Cell membrane; Peripheral membrane protein (By similarity). | 30DS |
| KLMA_10736 | -5.4 | 3.1 | 0.007 | actin-like protein | ro-4 |  | Cytoplasm, cytoskeleton (By similarity). | S |
| KLMA_30610 | -5.4 | 3.8 | 0.005 | calcium-transporting ATPase 1 | PMR1 | K01537 | Golgi apparatus membrane; Multi-pass membrane protein. | S |
| KLMA_50449 | -5.3 | 7.6 | 0.000 | fungal_trans super family conserved domain |  |  |  | C |
| KLMA_10248 | -5.3 | 6.0 | 0.005 | probable protein kinase YGL059W | PKP2 |  | Mitochondrion matrix. | S |
| KLMA_40185 | -5.3 | 4.8 | 0.000 | UPF0363 protein YOR164C | GET4 |  | Cytoplasm. | S |
| KLMA_30717 | -5.3 | 6.4 | 0.000 | uncharacterized protein YKR096W |  |  |  | S |
| KLMA_40356 | -5.3 | 6.5 | 0.029 | protein ECM14 | ECM14 | K08783 | Vacuole. | S |
| KLMA_20033 | -5.3 | 6.9 | 0.001 | cytochrome c oxidase assembly protein COX15 | COX15 | K02259 | Mitochondrion inner membrane; Multi-pass membrane protein. | S |
| KLMA_50580 | -5.3 | 1.7 | 0.014 | ribosomal N-lysine methyltransferase 2 | RKM2 |  |  | S |
| KLMA_30142 | -5.2 | 6.0 | 0.000 | serine/threonine-protein phosphatase PP1-2 | GLC7 | K06269 | Cytoplasm. Nucleus. | C |
| KLMA_60076 | -5.2 | 2.7 | 0.004 | protein STU2 | STU2 |  | Cytoplasm, cytoskeleton, spindle pole body. Cytoplasm, cytoskeleton, spindle. | S |
| KLMA_30274 | -5.2 | 7.6 | 0.039 | fumarate reductase |  |  | Mitochondrion. Cytoplasm. | S |
| KLMA_40131 | -5.1 | 5.6 | 0.004 | bud site selection protein RAX2 | RAX2 |  | Cell membrane; Single-pass type I membrane protein. Bud neck. Bud tip. | S |
| KLMA_10532 | -5.1 | 7.9 | 0.034 | tryptophan permease | TAT2 |  | Membrane; Multi-pass membrane protein. | S |
| KLMA_60230 | -5.1 | 5.3 | 0.018 | transcription factor tau 55 kDa subunit | TFC7 | K15206 | Nucleus. | S |
| KLMA_10726 | -5.0 | 3.3 | 0.003 | mitochondrial protein PET191 | PET191 |  | Mitochondrion. | 30X |
| KLMA_50494 | -5.0 | 3.3 | 0.013 | structure-specific endonuclease subunit SLX4 | SLX4 | K15079 | Nucleus (By similarity). | S |
| KLMA_30249 | -5.0 | 5.5 | 0.009 | uncharacterized protein YOR051C | ETT1 |  | Nucleus (By similarity). | S |
| KLMA_50409 | -4.9 | 7.4 | 0.000 | flo11 super family | MUC1 | K01178 | Secreted, cell wall (Probable). Membrane; Lipid-anchor, GPI-anchor (Potential). | 30DS |
| KLMA_40162 | -4.9 | 2.3 | 0.034 | WD repeat-containing protein YDR128W | MTC5 |  | Vacuole membrane; Peripheral membrane protein. | S |
| KLMA_80050 | -4.8 | 6.9 | 0.049 | homocysteine S-methyltransferase 2 | SAM4 | K00547 | Cytoplasm. Nucleus. | S |
| KLMA_10683 | -4.8 | 7.2 | 0.040 | NAD(P)H-dependent D-xylose reductase | XYL1 |  |  | S |
| KLMA_30085 | -4.8 | 2.5 | 0.018 | uncharacterized protein YOR296W |  |  | Cytoplasm. | S |
| KLMA_20515 | -4.8 | 6.7 | 0.000 | protein ISD11 | ISD11 |  | Mitochondrion. | 30DS |
| KLMA_10437 | -4.7 | 3.2 | 0.045 | D-amino-acid oxidase | DAO1 | K00273 |  | S |
| KLMA_50415 | -4.7 | 3.6 | 0.006 | uncharacterized oxidoreductase YHL021C | AIM17 |  | Mitochondrion. | S |
| KLMA_80130 | -4.5 | 6.8 | 0.000 | homoisocitrate dehydrogenase | LYS12 | K05824 | Mitochondrion. | C |
| KLMA_20830 | -4.5 | 8.8 | 0.000 | lactose permease | LAC12 |  | Membrane; Multi-pass membrane protein. | 30DS |
| KLMA_20294 | -4.5 | 5.9 | 0.030 | rab GDP-dissociation inhibitor | GDI1 |  | Cytoplasm. | S |
| KLMA_10036 | -4.5 | 5.5 | 0.001 | pantothenate kinase | CAB1 | K09680 | Cytoplasm. Nucleus. | C |
| KLMA_30105 | -4.4 | 8.4 | 0.001 | acetolactate synthase | ILV2 | K01652 | Mitochondrion. | S |
| KLMA_40155 | -4.4 | 2.6 | 0.026 | ISWI one complex protein 2 | IOC2 |  | Nucleus. | S |
| KLMA_20194 | -4.3 | 4.8 | 0.018 | UPF0613 protein PB24D3.06c |  |  | Cytoplasm. Nucleus. | S |
| KLMA_20061 | -4.3 | 4.4 | 0.011 | nucleoporin NUP188 | NUP188 | K14311 | Nucleus, nuclear pore complex. Nucleus membrane; Peripheral membrane protein; Cytoplasmic side. Nucleus membrane; Peripheral membrane protein; Nucleoplasmic side. | S |
| KLMA_30047 | -4.3 | 6.8 | 0.047 | phosphomethylpyrimidine kinase THI20 | THI20 | K00877 |  | S |
| KLMA_20158 | -4.3 | 10.0 | 0.000 | alcohol dehydrogenase 4 | ADH4 | K13953 | Mitochondrion matrix. | 30X |
| KLMA_10257 | -4.3 | 3.9 | 0.006 | ERAD-associated E3 ubiquitin-protein ligase HRD1 | HRD1 | K10601 | Endoplasmic reticulum membrane; Multi-pass membrane protein (By similarity). | 30X |
| KLMA_70189 | -4.1 | 8.6 | 0.017 | protein MMF1 | MMF1 |  | Mitochondrion matrix. | 30DS |
| KLMA_50241 | -4.1 | 7.4 | 0.001 | pyridoxamine 5'-phosphate oxidase | PDX3 | K00275 |  | C |
| KLMA_40575 | -4.1 | 7.7 | 0.032 | endopolyphosphatase | PPN1 | K06018 | Vacuole membrane; Single-pass type II membrane protein. | S |
| KLMA_20723 | -4.1 | 5.9 | 0.046 | maltose O-acetyltransferase | maa |  |  | S |
| KLMA_20704 | -4.1 | 9.8 | 0.000 | CBM_21 super family | GAC1 |  |  | 30DS |
| KLMA_30640 | -4.0 | 8.3 | 0.008 | D-amino-acid oxidase | dao1 |  |  | C |
| KLMA_10462 | -4.0 | 12.4 | 0.000 | enolase | ENO | K01689 | Cytoplasm (By similarity). | S |
| KLMA_30337 | -4.0 | 6.6 | 0.043 | uncharacterized protein YMR196W |  |  |  | S |
| KLMA_20537 | -4.0 | 7.6 | 0.045 | PET20 super family | SUE1 |  | Mitochondrion envelope. | S |
| KLMA_10572 | -4.0 | 5.1 | 0.034 | adenylyl cyclase-associated protein | SRV2 |  | Cytoplasm, cytoskeleton, actin patch. | S |
| KLMA_10540 | -3.9 | 8.7 | 0.001 | phosphoglycerate kinase | PGK | K00927 | Cytoplasm (By similarity). | S |
| KLMA_80074 | -3.9 | 2.3 | 0.050 | thiamine pathway transporter THI73 | THI73 |  | Endoplasmic reticulum membrane; Multi-pass membrane protein. Cell membrane; Multi-pass membrane protein (Probable). | S |
| KLMA_80059 | -3.9 | 13.4 | 0.000 | glyceraldehyde-3-phosphate dehydrogenase 3 | GAP3 | K00134 | Cytoplasm (By similarity). | S |
| KLMA_40149 | -3.9 | 9.1 | 0.000 | hypothetical protein |  |  |  | 30DS |
| KLMA_40218 | -3.9 | 13.1 | 0.000 | glyceraldehyde-3-phosphate dehydrogenase 1 | GAP1 | K00134 | Cytoplasm (By similarity). | 30DS |
| KLMA_70406 | -3.8 | 3.4 | 0.031 | protein NBA1 | NBA1 |  | Bud neck. Cytoplasm. | S |
| KLMA_10514 | -3.8 | 10.4 | 0.000 | branched-chain-amino-acid aminotransferase | BAT1 | K00826 | Mitochondrion matrix. | C |
| KLMA_40338 | -3.8 | 8.2 | 0.047 | ATP phosphoribosyltransferase | HIS1 | K00765 | Cytoplasm (By similarity). | S |
| KLMA_80118 | -3.7 | 11.3 | 0.000 | histone H4 | HHF1 | K11254 | Nucleus (By similarity). Chromosome (By similarity). | S |
| KLMA_50531 | -3.7 | 9.1 | 0.018 | uncharacterized transporter YLR152C |  | K07088 | Membrane; Multi-pass membrane protein. | 30DS |
| KLMA_70312 | -3.6 | 4.5 | 0.033 | long-chain-fatty-acid--CoA ligase 2 | FAA2 | K01897 | Cytoplasm. Mitochondrion. | S |
| KLMA_70068 | -3.6 | 10.0 | 0.018 | 40S ribosomal protein S29 | RPS29 | K02980 |  | S |
| KLMA_80322 | -3.6 | 8.9 | 0.006 | cytochrome c oxidase subunit 7 |  | K02269 |  | 30DS |
| KLMA_50360 | -3.6 | 9.5 | 0.001 | hexose transporter 2 | KHT2 | K08139 | Membrane; Multi-pass membrane protein. | S |
| KLMA_70443 | -3.6 | 3.9 | 0.033 | probable 2-methylcitrate dehydratase | PDH1 | K01720 |  | C |
| KLMA_50104 | -3.5 | 7.3 | 0.028 | alanyl-tRNA synthetase | ALA1 | K01872 | Isoform Cytoplasmic: Cytoplasm.Isoform Mitochondrial: Mitochondrion. | S |
| KLMA_10827 | -3.5 | 5.6 | 0.006 | MFS_1 |  |  | Membrane; Multi-pass membrane protein. | 30DS |
| KLMA_70044 | -3.5 | 9.8 | 0.001 | sorbitol dehydrogenase 1 | SOR1 | K00008 |  | 30DS |
| KLMA_30198 | -3.5 | 5.2 | 0.015 | protein transport protein YIP1 | YIP1 |  | Endoplasmic reticulum membrane; Multi-pass membrane protein. Golgi apparatus membrane; Multi-pass membrane protein. | S |
| KLMA_20148 | -3.5 | 8.0 | 0.008 | actin-interacting protein 1 | AIP1 |  | Cytoplasm, cytoskeleton. Cytoplasm, cytoskeleton, actin patch. | S |
| KLMA_60042 | -3.5 | 8.2 | 0.003 | peroxisomal membrane protein PEX21 | PEX21 |  | Cytoplasm (By similarity). Peroxisome membrane; Peripheral membrane protein; Cytoplasmic side (By similarity). | 30DS |
| KLMA_60129 | -3.4 | 13.0 | 0.000 | histone H3 | HHT1 | K11253 | Nucleus (By similarity). Chromosome (By similarity). | 30DS |
| KLMA_60128 | -3.4 | 11.4 | 0.000 | histone H4 | HHF1 | K11254 | Nucleus (By similarity). Chromosome (By similarity). | S |
| KLMA_70118 | -3.3 | 13.0 | 0.000 | guanine nucleotide-binding protein subunit gamma |  | K07973 | Membrane; Peripheral membrane protein (By similarity). | S |
| KLMA_80013 | -3.3 | 8.0 | 0.008 | uncharacterized dipeptidase C965.12 |  | K01273 |  | S |
| KLMA_20042 | -3.3 | 8.7 | 0.003 | acetyl-CoA hydrolase | ACH1 | K01067 | Cytoplasm (By similarity). | 30DS |
| KLMA_20489 | -3.3 | 9.0 | 0.013 | GAL4-like Zn2Cys6 binuclear cluster DNA-binding domain |  |  |  | C |
| KLMA_40622 | -3.3 | 11.7 | 0.000 | hypothetical protein |  |  |  | C |
| KLMA_20063 | -3.2 | 10.5 | 0.002 | uncharacterized protein RSN1 | RSN1 |  | Membrane; Multi-pass membrane protein (Potential). | S |
| KLMA_10547 | -3.2 | 8.7 | 0.005 | high-affinity glucose transporter | HGT1 |  | Membrane; Multi-pass membrane protein. | 30DS |
| KLMA_70150 | -3.2 | 5.8 | 0.045 | methionine aminopeptidase 2 | MAP2 | K01265 | Cytoplasm (By similarity). | S |
| KLMA_40135 | -3.2 | 7.1 | 0.014 | structural maintenance of chromosomes protein 4 | SMC4 | K06675 | Nucleus. Cytoplasm. Chromosome. | S |
| KLMA_80174 | -3.1 | 8.9 | 0.019 | probable transporter AQR1 | AQR1 |  | Membrane; Multi-pass membrane protein. | C |
| KLMA_10100 | -3.1 | 7.3 | 0.017 | G1/S-specific cyclin CLN1 | CCN1 |  |  | S |
| KLMA_20821 | -3.1 | 6.6 | 0.020 | aminopeptidase Y | APE3 | K01264 | Vacuole. | S |
| KLMA_80225 | -3.1 | 6.3 | 0.042 | alpha-1,2-mannosyltransferase | KTR1 | K10967 | Golgi apparatus membrane; Single-pass type II membrane protein. | S |
| KLMA_40412 | -3.0 | 4.2 | 0.038 | uncharacterized protein YER079W |  |  |  | S |
| KLMA_30172 | -3.0 | 7.0 | 0.038 | UPF0675 protein YJL084C | ALY2 |  | Cytoplasm. | S |
| KLMA_60379 | -3.0 | 6.5 | 0.025 | recQ-mediated genome instability protein 1 | RMI1 | K15364 | Cytoplasm. Nucleus. | 30DS |
| KLMA_10040 | -2.9 | 19.0 | 0.000 | uncharacterized protein YDR524C-B |  |  |  | 30DS |
| KLMA_20832 | -2.9 | 5.9 | 0.035 | dethiobiotin synthetase | BIO4 | K01935 |  | 30DS |
| KLMA_30331 | -2.9 | 6.5 | 0.028 | hypothetical protein |  |  |  | 30DS |
| KLMA_50289 | -2.9 | 9.2 | 0.007 | vacuolar membrane protein YOR292C |  |  | Vacuole membrane; Multi-pass membrane protein. | 30DS |
| KLMA_80026 | -2.9 | 7.7 | 0.021 | phosphoacetylglucosamine mutase | PCM1 | K01836 |  | S |
| KLMA_50297 | -2.9 | 6.4 | 0.039 | threonine dehydratase | ILV1 | K01754 | Mitochondrion. | S |
| KLMA_20828 | -2.9 | 6.6 | 0.040 | rab proteins geranylgeranyltransferase component A | MRS6 |  |  | S |
| KLMA_30029 | -2.9 | 8.3 | 0.020 | anthranilate synthase component 2 | TRP3 | K01656 |  | S |
| KLMA_20582 | -2.8 | 10.7 | 0.001 | elongation factor 2 | EFT1 | K03234 | Cytoplasm (By similarity). | S |
| KLMA_20333 | -2.8 | 9.4 | 0.006 | galactokinase | GAL1 | K00849 |  | 30DS |
| KLMA_20324 | -2.8 | 11.0 | 0.001 | mitochondrial DNA replication protein YHM2 | YHM2 |  | Mitochondrion inner membrane; Multi-pass membrane protein. | S |
| KLMA_10558 | -2.8 | 9.6 | 0.007 | D-arabinitol 2-dehydrogenase [ribulose-forming] | ARDH |  |  | 30DS |
| KLMA_80221 | -2.7 | 8.2 | 0.023 | ribose-5-phosphate isomerase | RKI1 | K01807 | Cytoplasm (Potential). | S |
| KLMA_40045 | -2.7 | 11.5 | 0.001 | ATPase-stabilizing factor 15 kDa protein | STF2 |  | Mitochondrion. | 30DS |
| KLMA_40621 | -2.7 | 8.3 | 0.032 | hypothetical protein |  |  |  | S |
| KLMA_60392 | -2.6 | 9.4 | 0.009 | uncharacterized protein YPL039W |  |  |  | C |
| KLMA_30724 | -2.6 | 9.0 | 0.036 | probable pyridoxine biosynthesis protein SNZ3 | SNZ3 | K06215 |  | S |
| KLMA_50260 | -2.6 | 9.5 | 0.016 | cytochrome c peroxidase | CCP1 | K00428 | Mitochondrion matrix (By similarity). | S |
| KLMA_60075 | -2.6 | 9.4 | 0.027 | pyruvate decarboxylase | PDC1 | K01568 |  | S |
| KLMA_30719 | -2.6 | 7.7 | 0.046 | phosphoenolpyruvate carboxykinase [ATP] | PCK1 | K01610 |  | 30DS |
| KLMA_30575 | -2.6 | 5.8 | 0.049 | actin patches distal protein 1 | APD1 |  | Cytoplasm. Nucleus. | S |
| KLMA_40253 | -2.5 | 9.8 | 0.008 | cytochrome c oxidase subunit 6 | COX6 | K02264 | Mitochondrion inner membrane. | S |
| KLMA_20268 | -2.5 | 10.8 | 0.006 | 40S ribosomal protein S2 | RPS2 | K02981 | Cytoplasm. Nucleus, nucleolus. | S |
| KLMA_20299 | -2.5 | 8.8 | 0.041 | cytochrome c oxidase subunit 6A | COX13 | K02266 | Mitochondrion inner membrane. | S |
| KLMA_10179 | -2.4 | 11.2 | 0.004 | glycerol-3-phosphate dehydrogenase [NAD+] 1 | GPD1 | K00006 |  | S |
| KLMA_60472 | -2.4 | 9.5 | 0.020 | altered inheritance rate of mitochondria protein 38 | RCF2 |  | Mitochondrion membrane; Multi-pass membrane protein. | S |
| KLMA_20100 | -2.3 | 8.8 | 0.047 | reduced viability upon starvation protein 161 | RVS161 |  | Cytoplasm, cytoskeleton. | 30DS |
| KLMA_10425 | -2.3 | 10.2 | 0.027 | pre-mRNA-splicing factor RSE1 | RSE1 | K12830 | Nucleus (By similarity). | S |
| KLMA_70054 | -2.2 | 10.0 | 0.033 | GTP-binding protein YPT1 | YPT1 | K07874 | Endoplasmic reticulum membrane; Peripheral membrane protein. Golgi apparatus membrane; Peripheral membrane protein. Cytoplasm. | S |
| KLMA_50529 | -2.2 | 9.5 | 0.037 | HYALURONIC ACID-BINDING PROTEIN 4 |  |  |  | S |
| KLMA_20220 | -2.1 | 13.9 | 0.000 | 3-ketoacyl-CoA thiolase | POT1 | K00632 | Peroxisome. | 30DS |
| KLMA_60499 | -2.0 | 11.2 | 0.022 | serine/threonine-protein phosphatase PP2A-1 catalytic subunit | PPH21 | K04382 |  | S |
| KLMA_50482 | -1.8 | 11.5 | 0.036 | non-classical export protein 2 | NCE102 |  | Cell membrane; Multi-pass membrane protein. | 30DS |
| KLMA_40174 | -1.8 | 16.3 | 0.000 | uncharacterized cell wall protein YDR134C |  |  |  | S |
| KLMA_70062 | -1.7 | 11.5 | 0.048 | covalently-linked cell wall protein 14 |  |  |  | S |
| KLMA_80309 | -1.3 | 12.8 | 0.029 | elongation factor 1-alpha | TEF | K03231 | Cytoplasm. | S |

^a^Gene expression was significantly (FDR < 0.05) altered under the following conditions: S, 45D-specific down-regulation; C, commonly down-regulated under 30DS, 45D and 30X conditions; 30DS, down-regulated under 45D and 30DS conditions; 30X, down-regulated under 45D and 30X conditions.

**Table S15** GO terms enriched in significantly up-regulated genes under 45D condition

| GO.ID | Term | Annotated  gene^a^ | Significant^b^ | Expected^c^ | P-value^d^ | Genes |
| --- | --- | --- | --- | --- | --- | --- |
| GO:0034470 | ncRNA processing | 259 | 23 | 7.56 | 8.4e-07 | BUD23, CGR1, CSL4, DBP10, DBP9, DHR2, GRC3, IMP4, LCP5, LSM2, PUS4, RPF2, RRP42, RRP5, RRS1, RTT10, SLX9, SPB1, THG1, TRM9, TUM1, UTP25, UTP6 |
| GO:0042254 | ribosome biogenesis | 270 | 23 | 7.88 | 1.8e-06 | BRX1, BUD23, CAM1, CGR1, CSL4, DBP10, DBP9, DHR2, GRC3, IMP4, LCP5, LSM2, NMD3, RPF2, RPL8B, RPS26A, RRP42, RRP5, RRS1, SLX9, SPB1, UTP25, UTP6 |
| GO:0006364 | rRNA processing | 191 | 18 | 5.58 | 6.9e-06 | BUD23, CGR1, CSL4, DBP10, DBP9, DHR2, GRC3, IMP4, LCP5, LSM2, RPF2, RRP42, RRP5, RRS1, SLX9, SPB1, UTP25, UTP6 |
| GO:0022613 | ribonucleoprotein complex biogenesis | 317 | 24 | 9.26 | 8.3e-06 | BRX1, BUD23, CAM1, CGR1, CSL4, DBP10, DBP9, DHR2, GRC3, IMP4, LCP5, LSM2, NMD3, PKH1, RPF2, RPL8B, RPS26A, RRP42, RRP5, RRS1, SLX9, SPB1, UTP25, UTP6 |
| GO:0016072 | rRNA metabolic process | 196 | 18 | 5.72 | 1.0e-05 | BUD23, CGR1, CSL4, DBP10, DBP9, DHR2, GRC3, IMP4, LCP5, LSM2, RPF2, RRP42, RRP5, RRS1, SLX9, SPB1, UTP25, UTP6 |
| GO:0034660 | ncRNA metabolic process | 305 | 23 | 8.91 | 1.4e-05 | BUD23, CGR1, CSL4, DBP10, DBP9, DHR2, GRC3, IMP4, LCP5, LSM2, PUS4, RPF2, RRP42, RRP5, RRS1, RTT10, SLX9, SPB1, THG1, TRM9, TUM1, UTP25, UTP6 |
| GO:0006396 | RNA processing | 405 | 26 | 11.83 | 6.3e-05 | BUD23, CGR1, CSL4, DBP10, DBP9, DHR2, GRC3, IMP4, LCP5, LSM2, PKH1, PUS4, RPF2, RRP42, RRP5, RRS1, RTT10, SLX9, SPB1, SPP2, SYF2, THG1, TRM9, TUM1, UTP25, UTP6 |
| GO:0000466 | maturation of 5.8S rRNA from tricistronic rRNA transcript (SSU-rRNA, 5.8S rRNA, LSU-rRNA) | 46 | 7 | 1.34 | 0.00031 | BUD23, CSL4, RPF2, RRP42, RRP5, RRS1, UTP6 |
| GO:0000460 | maturation of 5.8S rRNA | 47 | 7 | 1.37 | 0.00036 | BUD23, CSL4, RPF2, RRP42, RRP5, RRS1, UTP6 |
| GO:0009451 | RNA modification | 53 | 7 | 1.55 | 0.00076 | BUD23, LCP5, PUS4, SPB1, THG1, TRM9, TUM1 |
| GO:0000469 | cleavage involved in rRNA processing | 39 | 6 | 1.14 | 0.00080 | BUD23, CSL4, RRP42, RRP5, RRS1, UTP6 |
| GO:0006231 | dTMP biosynthetic process | 2 | 2 | 0.06 | 0.00084 | CDC21, DCD1 |
| GO:0046073 | dTMP metabolic process | 2 | 2 | 0.06 | 0.00084 | CDC21, DCD1 |
| GO:0034641 | cellular nitrogen compound metabolic process | 1463 | 59 | 42.72 | 0.00085 | ARG82, ASN1, BUD23, CAM1, CAR1, CDC13, CDC21, CDC9, CDD1, CGR1, CSL4, CTF8, DAL1, DAS2, DBP10, DBP9, DCD1, DFR1, DHR2, DNA2, FMP30, GRC3, HIS5, IMP4, KLMA_20616, LCP5, LSM2, MCM4, MEC3, NMD4, OAF3, PCC1, PHR1, PKH1, POL4, PSD1, PUS4, RKR1, RPC19, RPF2, RRN10, RRP42, RRP5, RRS1, RTT10, SLN1, SLX9, SPB1, SPP2, SPT6, SYF2, TAH11, TEL1, THG1, TRM9, TUM1, UNG1, UTP25, UTP6 |
| GO:0090501 | RNA phosphodiester bond hydrolysis | 40 | 6 | 1.17 | 0.00092 | BUD23, CSL4, RRP42, RRP5, RRS1, UTP6 |
| GO:0006139 | nucleobase-containing compound metabolic process | 1330 | 54 | 38.84 | 0.00158 | ARG82, BUD23, CAM1, CDC13, CDC21, CDC9, CDD1, CGR1, CSL4, CTF8, DAL1, DAS2, DBP10, DBP9, DCD1, DFR1, DHR2, DNA2, GRC3, IMP4, KLMA_20616, LCP5, LSM2, MCM4, MEC3, NMD4, OAF3, PCC1, PHR1, PKH1, POL4, PUS4, RKR1, RPC19, RPF2, RRN10, RRP42, RRP5, RRS1, RTT10, SLN1, SLX9, SPB1, SPP2, SPT6, SYF2, TAH11, TEL1, THG1, TRM9, TUM1, UNG1, UTP25, UTP6 |
| GO:0090304 | nucleic acid metabolic process | 1152 | 48 | 33.64 | 0.00209 | ARG82, BUD23, CAM1, CDC13, CDC9, CGR1, CSL4, CTF8, DBP10, DBP9, DHR2, DNA2, GRC3, IMP4, KLMA_20616, LCP5, LSM2, MCM4, MEC3, NMD4, OAF3, PCC1, PHR1, PKH1, POL4, PUS4, RKR1, RPC19, RPF2, RRN10, RRP42, RRP5, RRS1, RTT10, SLN1, SLX9, SPB1, SPP2, SPT6, SYF2, TAH11, TEL1, THG1, TRM9, TUM1, UNG1, UTP25, UTP6 |
| GO:0046483 | heterocycle metabolic process | 1414 | 56 | 41.29 | 0.00227 | ARG82, BUD23, CAM1, CDC13, CDC21, CDC9, CDD1, CGR1, CSL4, CTF8, DAL1, DAS2, DBP10, DBP9, DCD1, DFR1, DHR2, DNA2, GRC3, HIS5, IMP4, KLMA_20616, LCP5, LIPB, LSM2, MCM4, MEC3, NMD4, OAF3, PCC1, PHR1, PKH1, POL4, PUS4, RKR1, RPC19, RPF2, RRN10, RRP42, RRP5, RRS1, RTT10, SLN1, SLX9, SPB1, SPP2, SPT6, SYF2, TAH11, TEL1, THG1, TRM9, TUM1, UNG1, UTP25, UTP6 |
| GO:0009157 | deoxyribonucleoside monophosphate biosynthetic process | 3 | 2 | 0.09 | 0.00249 | CDC21, DCD1 |
| GO:0009162 | deoxyribonucleoside monophosphate metabolic process | 3 | 2 | 0.09 | 0.00249 | CDC21, DCD1 |
| GO:0009176 | pyrimidine deoxyribonucleoside monophosphate metabolic process | 3 | 2 | 0.09 | 0.00249 | CDC21, DCD1 |
| GO:0009177 | pyrimidine deoxyribonucleoside monophosphate biosynthetic process | 3 | 2 | 0.09 | 0.00249 | CDC21, DCD1 |
| GO:0006807 | nitrogen compound metabolic process | 1621 | 62 | 47.33 | 0.00255 | ARG82, ASN1, BUD23, CAM1, CAR1, CDC13, CDC21, CDC9, CDD1, CGR1, CSL4, CTF8, DAL1, DAS2, DBP10, DBP9, DCD1, DFR1, DHR2, DNA2, FMP30, GRC3, HIS5, IMP4, KEI1, KLMA_20616, LCP5, LSM2, LYS21, MCM4, MEC3, NIT3, NMD4, OAF3, PCC1, PHR1, PKH1, POL4, PSD1, PUS4, RKR1, RPC19, RPF2, RRN10, RRP42, RRP5, RRS1, RTT10, SLN1, SLX9, SPB1, SPP2, SPT6, SYF2, TAH11, TEL1, THG1, TRM9, TUM1, UNG1, UTP25, UTP6 |
| GO:1901360 | organic cyclic compound metabolic process | 1454 | 57 | 42.46 | 0.00258 | ARG82, BUD23, CAM1, CDC13, CDC21, CDC9, CDD1, CGR1, CSL4, CTF8, DAL1, DAS2, DBP10, DBP9, DCD1, DFR1, DHR2, DNA2, GRC3, HIS5, IMP4, KLMA_20616, LCP5, LIPB, LSM2, MCM4, MEC3, NCP1, NMD4, OAF3, PCC1, PHR1, PKH1, POL4, PUS4, RKR1, RPC19, RPF2, RRN10, RRP42, RRP5, RRS1, RTT10, SLN1, SLX9, SPB1, SPP2, SPT6, SYF2, TAH11, TEL1, THG1, TRM9, TUM1, UNG1, UTP25, UTP6 |
| GO:0006725 | cellular aromatic compound metabolic process | 1395 | 55 | 40.74 | 0.00294 | ARG82, BUD23, CAM1, CDC13, CDC21, CDC9, CDD1, CGR1, CSL4, CTF8, DAL1, DAS2, DBP10, DBP9, DCD1, DFR1, DHR2, DNA2, GRC3, HIS5, IMP4, KLMA_20616, LCP5, LSM2, MCM4, MEC3, NMD4, OAF3, PCC1, PHR1, PKH1, POL4, PUS4, RKR1, RPC19, RPF2, RRN10, RRP42, RRP5, RRS1, RTT10, SLN1, SLX9, SPB1, SPP2, SPT6, SYF2, TAH11, TEL1, THG1, TRM9, TUM1, UNG1, UTP25, UTP6 |
| GO:0000447 | endonucleolytic cleavage in ITS1 to separate SSU-rRNA from 5.8S rRNA and LSU-rRNA from tricistronic rRNA transcript (SSU-rRNA, 5.8S rRNA, LSU-rRNA) | 23 | 4 | 0.67 | 0.00396 | BUD23, RRP5, RRS1, UTP6 |
| GO:0000478 | endonucleolytic cleavage involved in rRNA processing | 23 | 4 | 0.67 | 0.00396 | BUD23, RRP5, RRS1, UTP6 |
| GO:0000479 | endonucleolytic cleavage of tricistronic rRNA transcript (SSU-rRNA, 5.8S rRNA, LSU-rRNA) | 23 | 4 | 0.67 | 0.00396 | BUD23, RRP5, RRS1, UTP6 |
| GO:0090502 | RNA phosphodiester bond hydrolysis, endonucleolytic | 24 | 4 | 0.7 | 0.00465 | BUD23, RRP5, RRS1, UTP6 |
| GO:0000301 | retrograde transport, vesicle recycling within Golgi | 4 | 2 | 0.12 | 0.00488 | COG3, RUD3 |
| GO:0009219 | pyrimidine deoxyribonucleotide metabolic process | 4 | 2 | 0.12 | 0.00488 | CDC21, DCD1 |
| GO:0009221 | pyrimidine deoxyribonucleotide biosynthetic process | 4 | 2 | 0.12 | 0.00488 | CDC21, DCD1 |
| GO:0009265 | 2'-deoxyribonucleotide biosynthetic process | 4 | 2 | 0.12 | 0.00488 | CDC21, DCD1 |
| GO:0009394 | 2'-deoxyribonucleotide metabolic process | 4 | 2 | 0.12 | 0.00488 | CDC21, DCD1 |
| GO:0019692 | deoxyribose phosphate metabolic process | 4 | 2 | 0.12 | 0.00488 | CDC21, DCD1 |
| GO:0046385 | deoxyribose phosphate biosynthetic process | 4 | 2 | 0.12 | 0.00488 | CDC21, DCD1 |
| GO:0009129 | pyrimidine nucleoside monophosphate metabolic process | 13 | 3 | 0.38 | 0.00559 | CDC21, DAS2, DCD1 |
| GO:0009130 | pyrimidine nucleoside monophosphate biosynthetic process | 13 | 3 | 0.38 | 0.00559 | CDC21, DAS2, DCD1 |
| GO:0006284 | base-excision repair | 14 | 3 | 0.41 | 0.00697 | CDC9, POL4, UNG1 |
| GO:0000462 | maturation of SSU-rRNA from tricistronic rRNA transcript (SSU-rRNA, 5.8S rRNA, LSU-rRNA) | 44 | 5 | 1.28 | 0.00848 | BUD23, DHR2, RRP5, RRS1, UTP6 |
| GO:0000956 | nuclear-transcribed mRNA catabolic process | 63 | 6 | 1.84 | 0.00948 | CSL4, KLMA_20616, LSM2, NMD4, PKH1, RRP42 |
| GO:0006402 | mRNA catabolic process | 63 | 6 | 1.84 | 0.00948 | CSL4, KLMA_20616, LSM2, NMD4, PKH1, RRP42 |

^a^The number of GO term annotated genes in the *K. marxianus* genome.

^b^The number of GO term annotated genes, which were significantly (FDR < 0.05) expressed under the condition.

^c^The expected value of Fisher's exact test.

^d^The P-value of Fisher's exact test.

**Table S16** Summary of significantly up-regulated genes under the 45D condition

| Locus_tag | logFC | logCPM | FDR | Product | UniProt gene | KO number | Localization | Specific^a^ |
| --- | --- | --- | --- | --- | --- | --- | --- | --- |
| KLMA_R308 | 19.1 | 8.6 | 0.000 | Lys-tRNA |  |  |  | 30DS |
| KLMA_80413 | 18.1 | 9.5 | 0.000 | 60S ribosomal protein L8-B | RPL8B | K02936 | Cytoplasm. | C |
| KLMA_R519 | 17.7 | 7.2 | 0.000 | Gly-tRNA |  |  |  | 30DS |
| KLMA_20492 | 16.5 | 6.0 | 0.000 | 40S ribosomal protein S26-A | RPS26A | K02976 | Cytoplasm. | S |
| KLMA_R413 | 16.0 | 5.5 | 0.001 | Arg-tRNA |  |  |  | S |
| KLMA_70354 | 15.6 | 6.2 | 0.001 | ADP,ATP carrier protein | AAC | K05863 | Mitochondrion inner membrane; Multi-pass membrane protein. | 30DS |
| KLMA_10783 | 15.0 | 7.0 | 0.000 | sorbose reductase SOU1 | SOU1 |  |  | C |
| KLMA_R127 | 15.0 | 4.9 | 0.000 | Val-tRNA |  |  |  | C |
| KLMA_R803 | 15.0 | 4.5 | 0.000 | Gly-tRNA |  |  |  | S |
| KLMA_40221 | 14.8 | 5.4 | 0.002 | 40S ribosomal protein S9 | MRPS9 |  | Mitochondrion (Potential). | 30X |
| KLMA_R416 | 14.8 | 4.5 | 0.000 | Val-tRNA |  |  |  | C |
| KLMA_20616 | 14.7 | 4.4 | 0.001 | uncharacterized protein YDR370C |  |  | Cytoplasm. | 30DS |
| KLMA_30150 | 14.6 | 4.1 | 0.001 | uncharacterized phosphatase YNL010W |  |  | Cytoplasm. Nucleus. | S |
| KLMA_R805 | 14.4 | 4.0 | 0.001 | Leu-tRNA |  |  |  | 30X |
| KLMA_R322 | 14.3 | 4.0 | 0.000 | Gln-tRNA |  |  |  | C |
| KLMA_R404 | 14.3 | 3.8 | 0.000 | Leu-tRNA |  |  |  | S |
| KLMA_R111 | 14.0 | 3.5 | 0.001 | Arg-tRNA |  |  |  | S |
| KLMA_20293 | 14.0 | 4.2 | 0.001 | U6 snRNA-associated Sm-like protein LSm2 | LSM2 | K12621 | Nucleus. Cytoplasm (Probable). | 30X |
| KLMA_R113 | 13.9 | 3.4 | 0.001 | Ala-tRNA |  |  |  | S |
| KLMA_R815 | 13.9 | 3.4 | 0.001 | His-tRNA |  |  |  | S |
| KLMA_R311 | 13.8 | 3.3 | 0.001 | Gln-tRNA |  |  |  | S |
| KLMA_R122 | 13.6 | 3.1 | 0.004 | Met-tRNA |  |  |  | S |
| KLMA_30371 | 13.5 | 5.8 | 0.030 | calcium/calmodulin-dependent protein kinase II | CMK2 | K00908 |  | 30DS |
| KLMA_30641 | 13.4 | 6.2 | 0.004 | probable hydrolase NIT3 | NIT3 |  |  | C |
| KLMA_R806 | 13.2 | 2.7 | 0.001 | Lys-tRNA |  |  |  | S |
| KLMA_R102 | 13.0 | 2.5 | 0.002 | Glu-tRNA |  |  |  | S |
| KLMA_R411 | 12.9 | 2.9 | 0.002 | Leu-tRNA |  |  |  | 30X |
| KLMA_R602 | 12.8 | 2.8 | 0.000 | Ala-tRNA |  |  |  | C |
| KLMA_R220 | 12.5 | 2.1 | 0.001 | Met-tRNA |  |  |  | S |
| KLMA_60474 | 12.4 | 4.5 | 0.001 | putative methyltransferase BUD23 | BUD23 |  | Cytoplasm. Nucleus. | C |
| KLMA_20507 | 12.3 | 2.3 | 0.021 | uncharacterized protein YGR127W |  |  |  | S |
| KLMA_20394 | 12.3 | 1.8 | 0.006 | actin-related protein 2/3 complex subunit 2 | ARC35 | K05758 | Cytoplasm, cytoskeleton, actin patch. | S |
| KLMA_R508 | 12.1 | 1.6 | 0.009 | Asn-tRNA |  |  |  | S |
| KLMA_R608 | 12.1 | 1.7 | 0.000 | Met-tRNA |  |  |  | 30DS |
| KLMA_R225 | 12.0 | 1.5 | 0.006 | Gln-tRNA |  |  |  | S |
| KLMA_R110 | 11.8 | 1.8 | 0.001 | Ala-tRNA |  |  |  | 30X |
| KLMA_R601 | 11.7 | 1.4 | 0.005 | Ala-tRNA |  |  |  | S |
| KLMA_R812 | 11.6 | 1.1 | 0.037 | Arg-tRNA |  |  |  | S |
| KLMA_20496 | 11.6 | 2.5 | 0.005 | hypothetical protein |  | K11098 |  | C |
| KLMA_50300 | 11.5 | 5.9 | 0.001 | hypothetical protein |  |  |  | 30X |
| KLMA_50196 | 11.5 | 1.0 | 0.003 | 1,3-beta-glucanosyltransferase GAS2 | GAS2 |  | Cell membrane; Lipid-anchor, GPI-anchor (By similarity). | S |
| KLMA_20072 | 11.5 | 2.0 | 0.036 | hypothetical conserved protein |  |  |  | S |
| KLMA_80165 | 11.2 | 1.6 | 0.048 | histidinol-phosphate aminotransferase | HIS5 | K00817 |  | S |
| KLMA_60493 | 11.1 | 3.8 | 0.033 | homocitrate synthase | LYS21 | K01655 | Mitochondrion (Potential). | C |
| KLMA_R525 | 10.8 | 5.9 | 0.001 | Leu-tRNA |  |  |  | S |
| KLMA_30167 | 10.7 | 2.1 | 0.001 | hypothetical protein |  |  |  | C |
| KLMA_R814 | 10.5 | 0.1 | 0.026 | Asn-tRNA |  |  |  | S |
| KLMA_80091 | 10.3 | 5.9 | 0.004 | exosome complex component RRP42 | RRP42 | K12589 | Cytoplasm. Nucleus, nucleolus. | 30DS |
| KLMA_20439 | 9.8 | 0.1 | 0.035 | protein GRC3 | GRC3 | K06947 | Nucleus, nucleolus (By similarity). | S |
| KLMA_60403 | 9.7 | 6.1 | 0.000 | putative elongation factor 1 gamma homolog | CAM1 | K03233 | Cytoplasm. Nucleus. | C |
| KLMA_30374 | 8.7 | 3.8 | 0.006 | conserved oligomeric Golgi complex subunit 3 | COG3 |  | Golgi apparatus membrane; Peripheral membrane protein; Cytoplasmic side. | S |
| KLMA_R524 | 8.6 | 3.2 | 0.029 | Thr-tRNA |  |  |  | S |
| KLMA_R406 | 8.5 | 9.3 | 0.000 | Thr-tRNA |  |  |  | S |
| KLMA_30716 | 8.3 | 5.0 | 0.001 | polarized growth chromatin-associated controller 1 | PCC1 |  | Nucleus (Probable). Chromosome, telomere (Probable). | S |
| KLMA_R123 | 7.9 | 2.9 | 0.000 | Ala-tRNA |  |  |  | S |
| KLMA_R117 | 7.8 | 1.3 | 0.039 | Val-tRNA |  |  |  | S |
| KLMA_10516 | 7.6 | 4.0 | 0.019 | polygalacturonase | PGU1 | K01184 |  | C |
| KLMA_R521 | 7.5 | 5.4 | 0.000 | Thr-tRNA |  |  |  | S |
| KLMA_R317 | 7.4 | 8.7 | 0.006 | Ala-tRNA |  |  |  | S |
| KLMA_80402 | 7.3 | 4.4 | 0.008 | probable 26S proteasome complex subunit SEM1 |  | K10881 |  | 30X |
| KLMA_50617 | 7.3 | 6.0 | 0.000 | Golgin IMH1 |  |  |  | S |
| KLMA_20626 | 7.3 | 2.9 | 0.000 | mitochondrial DnaJ homolog 2 | MDJ2 |  | Mitochondrion inner membrane. | C |
| KLMA_R226 | 7.3 | 6.4 | 0.006 | Ala-tRNA |  |  |  | S |
| KLMA_70295 | 7.0 | 3.2 | 0.044 | mitochondrial import receptor subunit TOM5 |  |  |  | S |
| KLMA_20752 | 6.8 | 6.5 | 0.000 | deoxycytidylate deaminase | DCD1 | K01493 |  | 30DS |
| KLMA_20051 | 6.8 | 4.3 | 0.000 | rRNA-processing protein CGR1 | CGR1 | K14822 | Nucleus, nucleolus (By similarity). | C |
| KLMA_10151 | 6.8 | 2.8 | 0.044 | protein ECM25 | ECM25 |  | Cytoplasm. | S |
| KLMA_30567 | 6.7 | 2.8 | 0.000 | uncharacterized protein YMR098C | ATP25 |  | Mitochondrion inner membrane; Peripheral membrane protein; Matrix side (By similarity). | 30DS |
| KLMA_R304 | 6.5 | 5.0 | 0.000 | Val-tRNA |  |  |  | S |
| KLMA_70050 | 6.4 | 0.2 | 0.017 | probable metabolite transport protein YFL040W |  |  | Membrane; Multi-pass membrane protein. | S |
| KLMA_R120 | 6.4 | 4.6 | 0.022 | Gly-tRNA |  |  |  | S |
| KLMA_30140 | 6.3 | 9.2 | 0.041 | factor RRN10 | RRN10 | K15221 | Nucleus, nucleolus. | S |
| KLMA_70041 | 6.2 | 2.2 | 0.012 | regulator of free ubiquitin chains 1 | RFU1 |  | Endosome (By similarity). | S |
| KLMA_R211 | 6.1 | 5.7 | 0.000 | Ala-tRNA |  |  |  | S |
| KLMA_40596 | 6.1 | 6.2 | 0.004 | serine/threonine-protein phosphatase PP-Z1 | PPZ1 | K01090 |  | S |
| KLMA_R516 | 6.1 | 5.3 | 0.002 | Thr-tRNA |  |  |  | S |
| KLMA_R208 | 6.1 | 3.6 | 0.001 | Leu-tRNA |  |  |  | S |
| KLMA_10209 | 6.0 | 5.0 | 0.020 | ATP-dependent RNA helicase DBP9 | DBP9 | K14810 | Nucleus, nucleolus (By similarity). | S |
| KLMA_20271 | 5.9 | 0.2 | 0.049 | zinc finger DNA binding domain |  |  |  | S |
| KLMA_R421 | 5.8 | 1.8 | 0.042 | Lys-tRNA |  |  |  | S |
| KLMA_R809 | 5.8 | 3.4 | 0.000 | Ser-tRNA |  |  |  | S |
| KLMA_40375 | 5.7 | 4.3 | 0.002 | U3 small nucleolar ribonucleoprotein protein IMP4 | IMP4 | K14561 | Nucleus, nucleolus. | 30DS |
| KLMA_10814 | 5.7 | 6.7 | 0.001 | oxidored-like super family |  |  | Mitochondrion. | S |
| KLMA_R804 | 5.7 | 5.1 | 0.014 | Thr-tRNA |  |  |  | S |
| KLMA_R104 | 5.7 | 4.5 | 0.000 | Ser-tRNA |  |  |  | S |
| KLMA_70304 | 5.7 | 6.3 | 0.000 | transcription elongation factor SPT6 | SPT6 | K11292 | Nucleus (By similarity). | C |
| KLMA_R204 | 5.6 | 3.2 | 0.014 | Pro-tRNA |  |  |  | S |
| KLMA_10197 | 5.6 | 8.1 | 0.000 | flocculation protein FLO5 | FLO5 |  | Secreted, cell wall. Membrane; Lipid-anchor, GPI-anchor (Potential). | 30X |
| KLMA_20221 | 5.5 | 4.4 | 0.002 | thiamine transporter | THI72 | K03457 | Membrane; Multi-pass membrane protein. | S |
| KLMA_R103 | 5.4 | 5.3 | 0.002 | Ala-tRNA |  |  |  | S |
| KLMA_R115 | 5.4 | 2.0 | 0.045 | Thr-tRNA |  |  |  | S |
| KLMA_50212 | 5.4 | 5.2 | 0.015 | tRNA(His) guanylyltransferase | THG1 | K10761 |  | S |
| KLMA_10803 | 5.3 | 3.1 | 0.034 | N-acyl-phosphatidylethanolamine-hydrolyzing phospholipase D | FMP30 |  | Mitochondrion membrane; Single-pass membrane protein (Potential). | S |
| KLMA_50229 | 5.3 | 5.6 | 0.016 | putative uridine kinase YDR020C | DAS2 |  | Cytoplasm. Nucleus. | 30DS |
| KLMA_60046 | 5.3 | 7.0 | 0.003 | uncharacterized protein YGR237C |  |  |  | 30X |
| KLMA_R811 | 5.3 | 4.5 | 0.000 | Thr-tRNA |  |  |  | S |
| KLMA_R318 | 5.2 | 3.4 | 0.005 | Pro-tRNA |  |  |  | S |
| KLMA_50201 | 5.2 | 4.8 | 0.001 | ribonucleases P/MRP protein subunit POP6 |  | K14524 |  | C |
| KLMA_R124 | 5.2 | 6.0 | 0.000 | Asn-tRNA |  |  |  | S |
| KLMA_R412 | 5.2 | 5.0 | 0.006 | Asp-tRNA |  |  |  | S |
| KLMA_R301 | 5.2 | 4.7 | 0.000 | Thr-tRNA |  |  |  | S |
| KLMA_30657 | 5.1 | 5.7 | 0.000 | protein ERD1 | ERD1 |  | Endoplasmic reticulum membrane; Multi-pass membrane protein. | S |
| KLMA_R302 | 5.0 | 3.1 | 0.034 | Val-tRNA |  |  |  | S |
| KLMA_R504 | 5.0 | 7.1 | 0.001 | Asp-tRNA |  |  |  | S |
| KLMA_40508 | 5.0 | 2.2 | 0.045 | chromosome transmission fidelity protein 8 | CTF8 | K11270 | Nucleus. | S |
| KLMA_20313 | 4.9 | 1.5 | 0.030 | uncharacterized protein YMR244W |  |  | Membrane; Single-pass membrane protein (Potential). | S |
| KLMA_80396 | 4.8 | 5.2 | 0.034 | UPF0596 Golgi apparatus membrane protein YDR367W | KEI1 |  | Golgi apparatus membrane; Multi-pass membrane protein. | S |
| KLMA_50242 | 4.8 | 0.6 | 0.043 | protein RAD61 |  |  |  | S |
| KLMA_R901 | 4.8 | 2.9 | 0.017 | Tyr-tRNA |  |  |  | S |
| KLMA_40579 | 4.8 | 4.4 | 0.000 | U3 small nucleolar RNA-associated protein 6 | UTP6 | K14557 | Nucleus, nucleolus. | S |
| KLMA_60349 | 4.8 | 4.5 | 0.001 | spindle pole body component SPC105 | SPC105 | K11563 | Cytoplasm, cytoskeleton, spindle pole body. Nucleus membrane; Peripheral membrane protein; Nucleoplasmic side. Chromosome, centromere, kinetochore. | C |
| KLMA_30083 | 4.7 | 4.4 | 0.036 | regulator of ribosome biosynthesis | RRS1 | K14852 | Nucleus. | S |
| KLMA_50574 | 4.7 | 4.6 | 0.026 | uncharacterized membrane protein YLR241W |  |  | Membrane; Multi-pass membrane protein. | S |
| KLMA_90008 | 4.7 | 1.8 | 0.029 | cytochrome c oxidase subunit 1 | COX1 | K02256 | Mitochondrion inner membrane; Multi-pass membrane protein. | S |
| KLMA_60269 | 4.7 | 4.5 | 0.011 | lysine-rich arabinogalactan protein 19 |  |  |  | S |
| KLMA_10192 | 4.6 | 1.2 | 0.041 | allantoinase | DAL1 | K01466 |  | S |
| KLMA_30303 | 4.6 | 5.4 | 0.001 | cell division control protein 54 | MCM4 | K02212 | Nucleus (By similarity). | S |
| KLMA_70154 | 4.5 | 3.5 | 0.006 | serine/threonine-protein kinase TEL1 | TEL1 | K04728 | Nucleus (By similarity). Chromosome, telomere (By similarity). | S |
| KLMA_30711 | 4.5 | 2.3 | 0.031 | osmosensing histidine protein kinase SLN1 | SLN1 | K11231 | Cell membrane; Multi-pass membrane protein (Potential). | S |
| KLMA_60179 | 4.5 | 4.5 | 0.008 | probable ATP-dependent RNA helicase DHR2 | DHR2 | K14781 | Nucleus, nucleolus. | 30DS |
| KLMA_10730 | 4.5 | 7.3 | 0.012 | mito_carr super family |  |  |  | S |
| KLMA_50285 | 4.4 | 3.6 | 0.006 | nonsense-mediated decay protein 4 | NMD4 |  | Cytoplasm (By similarity). | S |
| KLMA_20751 | 4.4 | 5.5 | 0.001 | phosphatidylserine decarboxylase proenzyme 1 | PSD1 | K01613 | Mitochondrion inner membrane. | 30DS |
| KLMA_50024 | 4.3 | 5.6 | 0.001 | arginase | CAR1 | K01476 | Cytoplasm (By similarity). | S |
| KLMA_10711 | 4.3 | 4.8 | 0.004 | hypothetical protein | TAH11 | K12414 | Cytoplasm. Nucleus. | S |
| KLMA_50291 | 4.3 | 2.8 | 0.017 | rRNA biogenesis protein RRP5 | RRP5 | K14792 | Nucleus, nucleolus. | 30DS |
| KLMA_60236 | 4.2 | 4.3 | 0.025 | DNA-directed RNA polymerases I and III subunit RPAC2 | RPC19 | K03020 | Nucleus, nucleolus. | S |
| KLMA_20604 | 4.2 | 4.1 | 0.004 | isoamyl acetate-hydrolyzing esterase | IAH1 |  |  | S |
| KLMA_50097 | 4.2 | 2.6 | 0.018 | hypothetical protein |  |  |  | S |
| KLMA_20725 | 4.2 | 5.6 | 0.019 | NADPH--cytochrome P450 reductase | NCP1 | K00327 | Endoplasmic reticulum membrane; Single-pass membrane protein. Mitochondrion outer membrane; Single-pass membrane protein. Cell membrane; Single-pass membrane protein. Microsome. | 30DS |
| KLMA_10363 | 4.2 | 6.6 | 0.001 | uncharacterized transcriptional regulatory protein YKR064W | OAF3 |  | Cytoplasm (By similarity). Nucleus (By similarity). Mitochondrion (By similarity). | S |
| KLMA_50581 | 4.1 | 2.8 | 0.011 | octanoyltransferase | LIPB | K03801 | Mitochondrion (Probable). | S |
| KLMA_40600 | 4.1 | 5.4 | 0.027 | tRNA (uracil-5-)-methyltransferase TRM9 | TRM9 | K15444 | Cytoplasm. Nucleus. | S |
| KLMA_40391 | 4.1 | 6.9 | 0.005 | malate dehydrogenase | MDH2 | K00026 | Cytoplasm. | C |
| KLMA_40320 | 4.0 | 6.1 | 0.009 | uncharacterized protein YOL098C |  | K06972 |  | S |
| KLMA_50622 | 4.0 | 5.7 | 0.002 | exosome complex component CSL4 | CSL4 | K07573 | Cytoplasm. Nucleus, nucleolus. | S |
| KLMA_30442 | 4.0 | 6.2 | 0.005 | F-box protein YDR306C |  |  |  | S |
| KLMA_30691 | 4.0 | 4.9 | 0.005 | conserved hypothetical protein | KEG1 |  | Endoplasmic reticulum membrane; Multi-pass membrane protein. | S |
| KLMA_50600 | 4.0 | 6.0 | 0.001 | uncharacterized protein YNL213C | RRG9 |  | Mitochondrion (By similarity). | S |
| KLMA_70395 | 3.9 | 4.5 | 0.005 | tRNA pseudouridine synthase 4 | PUS4 | K03177 | Nucleus. Mitochondrion. | C |
| KLMA_80126 | 3.8 | 8.1 | 0.001 | uncharacterized protein YIL091C | UTP25 | K14774 | Nucleus, nucleolus (By similarity). | 30DS |
| KLMA_R224 | 3.8 | 5.3 | 0.003 | Gln-tRNA |  |  |  | S |
| KLMA_60359 | 3.8 | 6.7 | 0.003 | spindle assembly checkpoint component MAD1 | MAD1 | K06679 | Nucleus (By similarity). | S |
| KLMA_60500 | 3.8 | 4.7 | 0.003 | probable allantoinase 1 |  |  |  | S |
| KLMA_R105 | 3.7 | 5.1 | 0.011 | Leu-tRNA |  |  |  | S |
| KLMA_40589 | 3.7 | 4.3 | 0.006 | uracil-DNA glycosylase | UNG1 | K03648 | Mitochondrion. Nucleus. | S |
| KLMA_R209 | 3.7 | 4.6 | 0.049 | Gly-tRNA |  |  |  | S |
| KLMA_10284 | 3.7 | 3.4 | 0.020 | hypothetical protein | SPP2 |  | Nucleus (By similarity). | S |
| KLMA_10366 | 3.7 | 5.6 | 0.014 | hypothetical protein |  |  |  | S |
| KLMA_40529 | 3.7 | 7.4 | 0.046 | transcriptional regulatory protein ASH1 |  |  |  | S |
| KLMA_20410 | 3.7 | 6.6 | 0.013 | protein transport protein USE1 | USE1 | K08507 | Endoplasmic reticulum membrane; Single-pass type IV membrane protein. | S |
| KLMA_80011 | 3.6 | 6.0 | 0.004 | deoxyribodipyrimidine photo-lyase | PHR1 | K01669 | Nucleus. Mitochondrion. | 30X |
| KLMA_60322 | 3.6 | 3.9 | 0.027 | TEL2-interacting protein 1 | TTI1 |  | Cytoplasm. Nucleus (Probable). | S |
| KLMA_40215 | 3.6 | 7.0 | 0.006 | cell division control protein 13 | CDC13 | K11115 | Chromosome, telomere. | 30X |
| KLMA_20803 | 3.5 | 4.0 | 0.031 | GRIP domain-containing protein RUD3 | RUD3 |  | Golgi apparatus lumen. | S |
| KLMA_50458 | 3.5 | 6.8 | 0.006 | uncharacterized protein YGR111W |  |  |  | 30DS |
| KLMA_10290 | 3.5 | 5.3 | 0.005 | IBN_N super family | SXM1 |  | Cytoplasm. Nucleus, nuclear pore complex. | S |
| KLMA_30020 | 3.5 | 5.7 | 0.017 | AP-1 accessory protein LAA1 |  |  |  | S |
| KLMA_20813 | 3.5 | 8.9 | 0.002 | tyrosine-protein phosphatase 2 | PTP2 | K01104 | Cytoplasm. Nucleus. | C |
| KLMA_10418 | 3.5 | 5.2 | 0.009 | ribosome biogenesis protein RPF2 | RPF2 | K14847 | Nucleus, nucleolus. | 30DS |
| KLMA_80364 | 3.5 | 6.7 | 0.009 | diphthamide biosynthesis protein 3 | DPH3 | K15455 | Cytoplasm (By similarity). Nucleus (By similarity). | S |
| KLMA_50277 | 3.5 | 3.8 | 0.019 | conserved hypothetical protein |  |  | Nucleus (Potential). | 30X |
| KLMA_20193 | 3.5 | 5.4 | 0.009 | peroxisomal long-chain fatty acid import protein 2 | PXA1 | K15628 | Peroxisome membrane; Multi-pass membrane protein. | 30X |
| KLMA_10277 | 3.4 | 4.9 | 0.012 | hypothetical protein |  |  |  | S |
| KLMA_30272 | 3.4 | 5.6 | 0.014 | DUF676 super family[cl10636] |  |  | Lipid droplet. Membrane; Single-pass membrane protein (Potential). | S |
| KLMA_60347 | 3.4 | 2.5 | 0.033 | uncharacterized WD repeat-containing protein YPL183C | RTT10 |  | Cytoplasm. Endosome. | S |
| KLMA_10414 | 3.4 | 3.8 | 0.026 | thymidylate synthase | CDC21 | K00560 | Nucleus. | 30DS |
| KLMA_20779 | 3.4 | 7.0 | 0.007 | dihydrofolate reductase | DFR1 | K00287 |  | S |
| KLMA_50131 | 3.3 | 6.7 | 0.015 | uncharacterized protein YLR290C |  |  | Mitochondrion. | S |
| KLMA_30560 | 3.3 | 6.8 | 0.011 | RING finger protein YMR247C | RKR1 |  | Nucleus. | S |
| KLMA_30086 | 3.3 | 6.7 | 0.019 | meiotically up-regulated gene 14 protein | mug14 |  | Cytoplasm. Nucleus. | S |
| KLMA_40432 | 3.3 | 5.1 | 0.015 | 60S ribosomal export protein NMD3 | NMD3 | K07562 | Cytoplasm. Nucleus, nucleoplasm. | 30DS |
| KLMA_20509 | 3.2 | 6.5 | 0.012 | pre-mRNA-splicing factor SYF2 | SYF2 | K12868 | Nucleus (By similarity). | S |
| KLMA_20535 | 3.2 | 6.6 | 0.011 | ubiquitin-conjugating enzyme E2-21 kDa |  | K10689 |  | S |
| KLMA_30507 | 3.2 | 5.1 | 0.014 | ribosome biogenesis protein SLX9 | SLX9 | K14804 | Nucleus, nucleolus (By similarity). | S |
| KLMA_60277 | 3.1 | 5.2 | 0.014 | ATP-dependent RNA helicase DBP10 | DBP10 | K14808 | Nucleus, nucleolus (By similarity). | 30DS |
| KLMA_10031 | 3.1 | 4.2 | 0.026 | adoMet-dependent rRNA methyltransferase SPB1 | SPB1 | K14857 | Nucleus, nucleolus (By similarity). | 30DS |
| KLMA_10328 | 3.1 | 5.7 | 0.025 | DNA replication ATP-dependent helicase DNA2 | DNA2 | K10742 | Nucleus. Chromosome. | 30DS |
| KLMA_60136 | 3.0 | 4.3 | 0.035 | ribosome biogenesis protein BRX1 | BRX1 | K14820 | Nucleus, nucleolus. | 30DS |
| KLMA_20219 | 3.0 | 6.7 | 0.037 | BNI1-related protein 1 | BNR1 |  |  | 30X |
| KLMA_50518 | 3.0 | 3.8 | 0.036 | ribonuclease MRP protein subunit RMP1 |  | K14532 |  | S |
| KLMA_10221 | 3.0 | 3.7 | 0.046 | DNA damage checkpoint control protein MEC3 | MEC3 | K02544 | Nucleus (Potential). | S |
| KLMA_30690 | 3.0 | 5.9 | 0.017 | ER-localized J domain-containing protein 5 | ERJ5 |  | Endoplasmic reticulum membrane; Single-pass type I membrane protein. | S |
| KLMA_10542 | 2.9 | 7.6 | 0.038 | HAD_like super family |  |  |  | S |
| KLMA_50565 | 2.9 | 5.9 | 0.049 | cytidine deaminase | CDD1 | K01489 |  | S |
| KLMA_10541 | 2.9 | 7.7 | 0.034 | DNA polymerase IV | POL4 | K10981 | Nucleus. | S |
| KLMA_50536 | 2.9 | 4.1 | 0.046 | inositol polyphosphate multikinase | ARG82 | K00328 | Nucleus. | S |
| KLMA_40337 | 2.8 | 6.8 | 0.039 | protein SYG1 | SYG1 |  | Cell membrane; Multi-pass membrane protein. | S |
| KLMA_70281 | 2.8 | 10.9 | 0.019 | asparagine synthetase [glutamine-hydrolyzing] 1 | ASN1 | K01953 |  | S |
| KLMA_30143 | 2.7 | 10.4 | 0.011 | protein YIP4 | YIP4 |  | Golgi apparatus membrane; Multi-pass membrane protein. | S |
| KLMA_10082 | 2.7 | 5.1 | 0.041 | protein PLM2 | PLM2 |  | Nucleus (Probable). | C |
| KLMA_70415 | 2.7 | 8.2 | 0.036 | U3 small nucleolar ribonucleoprotein protein LCP5 | LCP5 | K14765 | Nucleus, nucleolus. | 30DS |
| KLMA_10608 | 2.7 | 6.6 | 0.045 | hypothetical protein |  |  |  | S |
| KLMA_50322 | 2.6 | 9.2 | 0.038 | DNA ligase 1 | CDC9 | K10747 | Isoform Mitochondrial: Mitochondrion. | S |
| KLMA_30602 | 2.6 | 7.7 | 0.048 | acetyl-coenzyme A synthetase 1 | ACS1 | K01895 | Microsome (Potential). Endoplasmic reticulum (Potential). | 30X |
| KLMA_30449 | 2.5 | 7.1 | 0.047 | putative thiosulfate sulfurtransferase | TUM1 | K01011 | Mitochondrion. Cytoplasm. | S |
| KLMA_80265 | 2.5 | 7.3 | 0.047 | probable serine/threonine-protein kinase YOL100W | PKH1 |  |  | S |
| KLMA_30555 | 2.4 | 11.3 | 0.003 | conserved hypothetical transmembrane protein |  |  |  | 30X |
| KLMA_20427 | 2.2 | 10.5 | 0.030 | hypothetical protein |  |  |  | 30X |

^a^Gene expression was significantly (FDR < 0.05) altered under the following conditions: S, 45D-specific up-regulation; C, commonly up-regulated under 30DS, 45D and 30X conditions; 30DS, up-regulated under 45D and 30DS conditions; 30X, up-regulated under 45D and 30X conditions.

**Table S17** Subcellular localization of products of significantly expressed genes

| Description | 30DS_up | | 30DS_down | | 45D_up | | 45D_down | | 30X_up | | 30X_down | |
| --- | --- | --- | --- | --- | --- | --- | --- | --- | --- | --- | --- | --- |
| Nucleus | 64 | 40.3% | 12 | 7.8% | 33 | 16.6% | 55 | 10.8% | 11 | 12.4% | 3 | 3.8% |
| Cytoplasm, nucleus | 23 | 14.5% | 14 | 9.1% | 15 | 7.5% | 47 | 9.3% | 6 | 6.7% | 11 | 13.9% |
| Membrane | 3 | 1.9% | 26 | 16.9% | 9 | 4.5% | 53 | 10.4% | 10 | 11.2% | 8 | 10.1% |
| Cytoplasm | 15 | 9.4% | 13 | 8.4% | 8 | 4.0% | 66 | 13.0% | 7 | 7.9% | 6 | 7.6% |
| Mitochondrion | 7 | 4.4% | 8 | 5.2% | 7 | 3.5% | 39 | 7.7% | 4 | 4.5% | 9 | 11.4% |
| Peroxisome, ER, golgi (membrane) | 1 | 0.6% | 3 | 1.9% | 7 | 3.5% | 30 | 5.9% | 1 | 1.1% | 3 | 3.8% |
| Mitochondrion (membrane) | 9 | 5.7% | 8 | 5.2% | 6 | 3.0% | 25 | 4.9% | 4 | 4.5% | 2 | 2.5% |
| Mitochondrion, nucleus | 2 | 1.3% |  | 0.0% | 3 | 1.5% | 1 | 0.2% | 2 | 2.2% |  | 0.0% |
| Peroxisome, ER, golgi |  | 0.0% | 3 | 1.9% | 2 | 1.0% | 6 | 1.2% | 4 | 4.5% |  | 0.0% |
| Cytoplasm (membrane) |  | 0.0% | 3 | 1.9% | 1 | 0.5% | 7 | 1.4% | 1 | 1.1% | 1 | 1.3% |
| Cytoplasm, mitochondrion |  | 0.0% | 2 | 1.3% | 1 | 0.5% | 6 | 1.2% |  | 0.0% | 1 | 1.3% |
| Cytoplasm, mitochondrion, nucleus | 1 | 0.6% |  | 0.0% | 1 | 0.5% |  | 0.0% |  | 0.0% |  | 0.0% |
| Cytoplasm, nucleus (membrane) | 1 | 0.6% |  | 0.0% | 1 | 0.5% | 3 | 0.6% | 1 | 1.1% |  | 0.0% |
| Cytoplasm, peroxisome, ER, golgi (membrane) |  | 0.0% | 1 | 0.6% | 1 | 0.5% | 9 | 1.8% |  | 0.0% |  | 0.0% |
| Mitochondrion, peroxisome, ER, golgi (membrane) | 1 | 0.6% | 2 | 1.3% | 1 | 0.5% | 2 | 0.4% | 1 | 1.1% | 1 | 1.3% |
| Cytoplasm, mitochondrion (membrane) |  | 0.0% |  | 0.0% |  | 0.0% | 1 | 0.2% |  | 0.0% |  | 0.0% |
| Cytoplasm, mitochondrion, nucleus (membrane) |  | 0.0% |  | 0.0% |  | 0.0% | 1 | 0.2% |  | 0.0% |  | 0.0% |
| Cytoplasm, mitochondrion, peroxisome, ER, golgi (membrane) | 1 | 0.6% |  | 0.0% |  | 0.0% | 1 | 0.2% |  | 0.0% |  | 0.0% |
| Cytoplasm, nucleus, peroxisome, ER, golgi |  | 0.0% |  | 0.0% |  | 0.0% | 1 | 0.2% |  | 0.0% |  | 0.0% |
| Cytoplasm, nucleus, peroxisome, ER, golgi (membrane) |  | 0.0% |  | 0.0% |  | 0.0% | 1 | 0.2% |  | 0.0% |  | 0.0% |
| Cytoplasm, peroxisome, ER, golgi |  | 0.0% |  | 0.0% |  | 0.0% | 1 | 0.2% |  | 0.0% |  | 0.0% |
| Nucleus (membrane) |  | 0.0% |  | 0.0% |  | 0.0% | 1 | 0.2% |  | 0.0% |  | 0.0% |
| Nucleus, peroxisome, ER, golgi (membrane) | 1 | 0.6% |  | 0.0% |  | 0.0% | 1 | 0.2% |  | 0.0% |  | 0.0% |
| tRNA | 7 | 4.4% | 5 | 3.2% | 57 | 28.6% | 4 | 0.8% | 7 | 7.9% | 2 | 2.5% |
| Not annotated | 23 | 14.5% | 54 | 35.1% | 46 | 23.1% | 147 | 28.9% | 30 | 33.7% | 32 | 40.5% |
| Total | 159 |  | 154 |  | 199 |  | 508 |  | 89 |  | 79 |  |

**Table S18** HSP and oxidative stress response genes

| **Heat shock protein genes** | | | |  | |  | |  |
| --- | --- | --- | --- | --- | --- | --- | --- | --- |
| Locus_tag | Product | | | UniProt | | UniProt_gene | | KO_number |
| KLMA_10712 | heat shock protein SSC1 | | | P12398 | |  | | K04043 |
| KLMA_10728 | peptidyl-prolyl cis-trans isomerase CYP7 | | | P47103 | | CPR7 | | K01802 |
| KLMA_10813 | heat shock protein homolog SSE1 | | | Q875P5 | | SSE1 | | K09485 |
| KLMA_20079 | hsp70 nucleotide exchange factor FES1 | | | Q6CNM7 | | FES1 | |  |
| KLMA_20282 | heat shock protein 60 | | | P19882 | | HSP60 | | K04077 |
| KLMA_20459 | heat shock protein SSA2 | | | P10592 | | SSA2 | | K03283 |
| KLMA_20771 | 12 kDa heat shock protein | | | P22943 | | HSP12 | |  |
| KLMA_30257 | protein SGT1 | | | Q08446 | | SGT1 | | K12795 |
| KLMA_30350 | hsp70/Hsp90 co-chaperone CNS1 | | | P33313 | | CNS1 | |  |
| KLMA_30434 | protein interacting with Hsp90 1 | | | P38768 | | PIH1 | |  |
| KLMA_30496 | TPR repeat-containing protein associated with Hsp90 | | | P25638 | | TAH1 | |  |
| KLMA_30546 | ribosome-associated complex subunit SSZ1 | | | P38788 | | SSZ1 | |  |
| KLMA_30551 | vesicular-fusion protein SEC18 | | | P18759 | | SEC18 | | K06027 |
| KLMA_30561 | co-chaperone protein SBA1 | | | P28707 | | SBA1 | |  |
| KLMA_40099 | heat shock protein 104 | | | P31539 | | HSP104 | |  |
| KLMA_40128 | heat shock protein 26 | | | P15992 | | HSP26 | | K13993 |
| KLMA_50124 | hsp90 co-chaperone AHA1 | | | Q12449 | | AHA1 | |  |
| KLMA_50253 | cell wall mannoprotein HSP150 | | | Q03178 | | PIR1 | |  |
| KLMA_50254 | cell wall mannoprotein HSP150 | | | A6ZZG0 | | PIR1 | |  |
| KLMA_50255 | cell wall mannoprotein HSP150 | | | Q03180 | | PIR3 | |  |
| KLMA_50274 | heat shock protein SSQ1 | | | Q05931 | | SSQ1 | |  |
| KLMA_50344 | ATP-dependent molecular chaperone HSC82 | | | P02829 | | HSP82 | | K04079 |
| KLMA_50469 | HSP70 co-chaperone SNL1 | | |  | |  | | K14017 |
| KLMA_50526 | hsp90 co-chaperone Cdc37 | | | P06101 | | CDC37 | | K09554 |
| KLMA_50592 | heat shock protein SSB | | | P41770 | | SSB | | K03283 |
| KLMA_60101 | heat shock protein 78 | | | P33416 | | HSP78 | | K03695 |
| KLMA_60191 | heat shock protein 70 homolog LHS1 | | | P36016 | | LHS1 | | K09486 |
| KLMA_60248 | mitochondrial import receptor subunit TOM70 | | | P07213 | | TOM70 | |  |
| KLMA_60484 | cell division control protein 48 | | | P25694 | | CDC48 | | K13525 |
| KLMA_60511 | peptidyl-prolyl cis-trans isomerase D | | | Q6CL78 | | CPR6 | | K05864 |
| KLMA_70283 | serine/threonine-protein phosphatase T | | | P53043 | | PPT1 | | K04460 |
| KLMA_80019 | probable chaperone protein HSP31 | | | Q04432 | | HSP31 | |  |
| KLMA_80087 | 10 kDa heat shock protein | | | P38910 | | HSP10 | | K04078 |
| KLMA_80278 | hsp90 co-chaperone HCH1 | | | P53834 | | HCH1 | |  |
| KLMA_80367 | heat shock protein SSA3 | | | P09435 | | SSA3 | | K03283 |
| **Oxidative stress response genes** | |  |  | |  | |  | |
| Locus_tag | Product | UniProt | UniProt gene | | KO number | | UniProt location | |
| KLMA_10058 | glutaredoxin-1 | P17695 | GRX2 | | K03676 | | Cytoplasm. Mitochondrion. | |
| KLMA_10060 | selR super family | P25566 | MXR2 | | K00391 | |  | |
| KLMA_10250 | YAP1-binding protein 1 | P38315 | YBP1 | |  | | Cytoplasm. | |
| KLMA_10673 | glutathione peroxidase 2 | P38143 | GPX2 | |  | |  | |
| KLMA_10682 | thioredoxin reductase | Q6HA24 | TRR1 | | K00384 | | Mitochondrion (Potential). | |
| KLMA_10787 | glutathione reductase | Q6HA23 | GLR1 | | K00383 | | Cytoplasm (By similarity). | |
| KLMA_20243 | thioredoxin-3 | P25372 | TRX3 | | K03671 | | Mitochondrion. | |
| KLMA_20370 | glutathione synthetase | Q08220 | GSH2 | | K01920 | |  | |
| KLMA_20407 | peroxiredoxin HYR1 | P40581 | HYR1 | | K00432 | | Cytoplasm. | |
| KLMA_20553 | superoxide dismutase [Mn] | P00447 | SOD2 | | K04564 | | Mitochondrion matrix. | |
| KLMA_30154 | glutamate--cysteine ligase | P32477 | GSH1 | | K11204 | |  | |
| KLMA_30286 | monothiol glutaredoxin-3 | Q03835 | GRX3 | |  | |  | |
| KLMA_30366 | monothiol glutaredoxin-5 | Q6YFE4 | GRX5 | | K07390 | | Mitochondrion matrix (By similarity). | |
| KLMA_30632 | peroxiredoxin-like protein DDB_G0282517 | P34227 | PRX1 | |  | | Mitochondrion. | |
| KLMA_30684 | peptide methionine sulfoxide reductase | P40029 | MXR1 | | K07304 | |  | |
| KLMA_40173 | peroxiredoxin type-2 | P38013 | AHP1 | | K14171 | | Cytoplasm. | |
| KLMA_40176 | metal resistance protein YCF1 | P39109 | YCF1 | |  | | Vacuole membrane; Multi-pass membrane protein. | |
| KLMA_40301 | UPF0067 GAF domain-containing protein YKL069W | P36088 |  | | K08968 | | Cytoplasm. Nucleus. | |
| KLMA_40313 | superoxide dismutase 1 copper chaperone | Q6CIG2 | CCS1 | |  | | Cytoplasm (By similarity). | |
| KLMA_40574 | peroxiredoxin TSA1 | P34760 | TSA1 | | K03386 | | Cytoplasm. | |
| KLMA_50005 | glutathione S-transferase 1 | P40582 | GTT1 | | K00799 | | Endoplasmic reticulum membrane; Peripheral membrane protein. | |
| KLMA_50279 | glutaredoxin-like protein YLR364W | Q05926 | GRX8 | |  | | Cytoplasm. | |
| KLMA_50406 | peroxiredoxin DOT5 | P40553 | DOT5 | | K03564 | | Nucleus. Chromosome, telomere (Potential). | |
| KLMA_50419 | catalase T | A6ZV70 | CTT1 | | K03781 | | Cytoplasm (By similarity). | |
| KLMA_60077 | thioredoxin-2 | P22803 | TRX2 | | K03671 | | Cytoplasm. Golgi apparatus membrane; Peripheral membrane protein. Nucleus. | |
| KLMA_60133 | monothiol glutaredoxin-7 | P38068 | GRX7 | |  | |  | |
| KLMA_60401 | peroxisomal catalase A | P15202 | CTA1 | | K03781 | | Peroxisome. | |
| KLMA_70087 | superoxide dismutase [Cu-Zn] | Q6CPE2 | SOD1 | | K04565 | | Cytoplasm (By similarity). | |
| KLMA_70261 | D-arabinono-1 | Q6CSY3 | ALO1 | | K00107 | | Mitochondrion membrane (By similarity). | |
| KLMA_80358 | mitochondrial peroxiredoxin PRX1 | P34227 | PRX1 | | K03386 | | Mitochondrion. | |

**Table S19** GO terms enriched in significantly up-regulated genes under 30X condition

| GO.ID | Term | Annotated  gene^a^ | Significant^b^ | Expected^c^ | P-value^d^ | Genes |
| --- | --- | --- | --- | --- | --- | --- |
| GO:0009062 | fatty acid catabolic process | 21 | 7 | 0.32 | 1.4e-08 | CIT3, FOX2, ICL2, POT1, POX1, PXA1, SOU1 |
| GO:0072329 | monocarboxylic acid catabolic process | 29 | 7 | 0.45 | 1.7e-07 | CIT3, FOX2, ICL2, POT1, POX1, PXA1, SOU1 |
| GO:0044242 | cellular lipid catabolic process | 38 | 7 | 0.59 | 1.2e-06 | CIT3, FOX2, ICL2, POT1, POX1, PXA1, SOU1 |
| GO:0006635 | fatty acid beta-oxidation | 16 | 5 | 0.25 | 2.8e-06 | FOX2, POT1, POX1, PXA1, SOU1 |
| GO:0019395 | fatty acid oxidation | 17 | 5 | 0.26 | 3.9e-06 | FOX2, POT1, POX1, PXA1, SOU1 |
| GO:0034440 | lipid oxidation | 17 | 5 | 0.26 | 3.9e-06 | FOX2, POT1, POX1, PXA1, SOU1 |
| GO:0006631 | fatty acid metabolic process | 64 | 8 | 0.99 | 4.4e-06 | CAT2, CIT3, FOX2, ICL2, POT1, POX1, PXA1, SOU1 |
| GO:0032787 | monocarboxylic acid metabolic process | 111 | 10 | 1.71 | 5.2e-06 | ACS1, ALD4, CAT2, CIT3, FOX2, ICL2, POT1, POX1, PXA1, SOU1 |
| GO:0016042 | lipid catabolic process | 51 | 7 | 0.79 | 9.7e-06 | CIT3, FOX2, ICL2, POT1, POX1, PXA1, SOU1 |
| GO:0044282 | small molecule catabolic process | 89 | 8 | 1.37 | 5.2e-05 | CIT3, DAK1, FOX2, ICL2, POT1, POX1, PXA1, SOU1 |
| GO:0030258 | lipid modification | 29 | 5 | 0.45 | 6.5e-05 | FOX2, POT1, POX1, PXA1, SOU1 |
| GO:0016054 | organic acid catabolic process | 68 | 7 | 1.05 | 6.6e-05 | CIT3, FOX2, ICL2, POT1, POX1, PXA1, SOU1 |
| GO:0046395 | carboxylic acid catabolic process | 68 | 7 | 1.05 | 6.6e-05 | CIT3, FOX2, ICL2, POT1, POX1, PXA1, SOU1 |
| GO:0055114 | oxidation-reduction process | 160 | 10 | 2.47 | 0.00013 | ALD4, CIT3, DAK1, FOX2, MDH2, POT1, POX1, PXA1, SOL3, SOU1 |
| GO:0019541 | propionate metabolic process | 3 | 2 | 0.05 | 0.00069 | CIT3, ICL2 |
| GO:0019543 | propionate catabolic process | 3 | 2 | 0.05 | 0.00069 | CIT3, ICL2 |
| GO:0019626 | short-chain fatty acid catabolic process | 3 | 2 | 0.05 | 0.00069 | CIT3, ICL2 |
| GO:0019629 | propionate catabolic process, 2-methylcitrate cycle | 3 | 2 | 0.05 | 0.00069 | CIT3, ICL2 |
| GO:0046459 | short-chain fatty acid metabolic process | 4 | 2 | 0.06 | 0.00138 | CIT3, ICL2 |
| GO:0019752 | carboxylic acid metabolic process | 345 | 13 | 5.32 | 0.00177 | ACS1, ALD4, ARG1, CAT2, CIT3, FOX2, ICL2, LYS21, MDH2, POT1, POX1, PXA1, SOU1 |
| GO:0043436 | oxoacid metabolic process | 354 | 13 | 5.46 | 0.00223 | ACS1, ALD4, ARG1, CAT2, CIT3, FOX2, ICL2, LYS21, MDH2, POT1, POX1, PXA1, SOU1 |
| GO:0019405 | alditol catabolic process | 5 | 2 | 0.08 | 0.00227 | DAK1, SOU1 |
| GO:0006082 | organic acid metabolic process | 356 | 13 | 5.49 | 0.00235 | ACS1, ALD4, ARG1, CAT2, CIT3, FOX2, ICL2, LYS21, MDH2, POT1, POX1, PXA1, SOU1 |
| GO:0016052 | carbohydrate catabolic process | 63 | 5 | 0.97 | 0.00259 | DAK1, GLK1, PGU1, SOL3, SOU1 |
| GO:0006083 | acetate metabolic process | 6 | 2 | 0.09 | 0.00337 | ACS1, ALD4 |
| GO:0016233 | telomere capping | 6 | 2 | 0.09 | 0.00337 | CDC13, POL12 |
| GO:0046174 | polyol catabolic process | 7 | 2 | 0.11 | 0.00467 | DAK1, SOU1 |
| GO:0007155 | cell adhesion | 8 | 2 | 0.12 | 0.00617 | FLO5, HSP12 |
| GO:0022610 | biological adhesion | 8 | 2 | 0.12 | 0.00617 | FLO5, HSP12 |
| GO:0019321 | pentose metabolic process | 10 | 2 | 0.15 | 0.00972 | SOL3, SOU1 |
| GO:0044712 | single-organism catabolic process | 282 | 10 | 4.35 | 0.00983 | CIT3, DAK1, FOX2, GLK1, ICL2, POT1, POX1, PXA1, SOL3, SOU1 |

^a^The number of GO term annotated genes in the *K. marxianus* genome.

^b^The number of GO term annotated genes, which were significantly (FDR < 0.05) expressed under the condition.

^c^The expected value of Fisher's exact test.

^d^The P-value of Fisher's exact test.

**Table S20** Summary of significantly up-regulated genes under the 30X condition

| Locus_tag | logFC | logCPM | FDR | Product | UniProt gene | KO number | Localization | Specific^a^ |
| --- | --- | --- | --- | --- | --- | --- | --- | --- |
| KLMA_30482 | 18.3 | 7.9 | 0.000 | glyoxalase super family protein |  |  |  | 30DS |
| KLMA_80413 | 18.3 | 9.5 | 0.000 | 60S ribosomal protein L8-B | RPL8B | K02936 | Cytoplasm. | C |
| KLMA_40373 | 17.4 | 7.0 | 0.001 | hypothetical protein | OM45 |  | Mitochondrion outer membrane. | 30DS |
| KLMA_10783 | 17.1 | 7.0 | 0.000 | sorbose reductase SOU1 | SOU1 |  |  | C |
| KLMA_30641 | 16.4 | 6.2 | 0.002 | probable hydrolase NIT3 | NIT3 |  |  | C |
| KLMA_50300 | 16.4 | 5.9 | 0.000 | hypothetical protein |  |  |  | 45D |
| KLMA_10180 | 15.8 | 7.3 | 0.015 | argininosuccinate synthase | ARG1 | K01940 | Cytoplasm. | 30DS |
| KLMA_10331 | 15.8 | 5.3 | 0.000 | 6-phosphogluconolactonase 3 | SOL3 | K01057 | Cytoplasm. Nucleus. | S |
| KLMA_10267 | 15.4 | 5.2 | 0.000 | SAP super family | AIM34 |  | Mitochondrion membrane; Single-pass membrane protein (By similarity). | 30DS |
| KLMA_10225 | 15.2 | 5.3 | 0.000 | GTP-binding nuclear protein GSP1/Ran | GSP1 | K07936 | Nucleus (By similarity). | 30DS |
| KLMA_40221 | 15.0 | 5.4 | 0.008 | 40S ribosomal protein S9 | MRPS9 |  | Mitochondrion (Potential). | 45D |
| KLMA_20293 | 13.3 | 4.2 | 0.008 | U6 snRNA-associated Sm-like protein LSm2 | LSM2 | K12621 | Nucleus. Cytoplasm (Probable). | 45D |
| KLMA_60474 | 13.2 | 4.5 | 0.003 | putative methyltransferase BUD23 | BUD23 |  | Cytoplasm. Nucleus. | C |
| KLMA_60493 | 13.1 | 3.8 | 0.040 | homocitrate synthase | LYS21 | K01655 | Mitochondrion (Potential). | C |
| KLMA_R127 | 13.1 | 4.9 | 0.001 | Val-tRNA |  |  |  | C |
| KLMA_R416 | 12.0 | 4.5 | 0.000 | Val-tRNA |  |  |  | C |
| KLMA_20460 | 11.9 | 1.4 | 0.037 | uncharacterized mitochondrial carrier YPR011C |  | K14684 | Mitochondrion inner membrane; Multi-pass membrane protein. | S |
| KLMA_R411 | 11.5 | 2.9 | 0.016 | Leu-tRNA |  |  |  | 45D |
| KLMA_20496 | 11.2 | 2.5 | 0.021 | hypothetical protein |  | K11098 |  | C |
| KLMA_R602 | 11.1 | 2.8 | 0.001 | Ala-tRNA |  |  |  | C |
| KLMA_30167 | 11.0 | 2.1 | 0.002 | hypothetical protein |  |  |  | C |
| KLMA_20617 | 10.7 | 0.9 | 0.005 | SAM50-like protein SpAC17C9.06 |  |  |  | 30DS |
| KLMA_R322 | 10.4 | 4.0 | 0.025 | Gln-tRNA |  |  |  | C |
| KLMA_R805 | 10.3 | 4.0 | 0.046 | Leu-tRNA |  |  |  | 45D |
| KLMA_R110 | 10.2 | 1.8 | 0.011 | Ala-tRNA |  |  |  | 45D |
| KLMA_60403 | 10.1 | 6.1 | 0.000 | putative elongation factor 1 gamma homolog | CAM1 | K03233 | Cytoplasm. Nucleus. | C |
| KLMA_10516 | 10.0 | 4.0 | 0.002 | polygalacturonase | PGU1 | K01184 |  | C |
| KLMA_20220 | 9.9 | 13.9 | 0.000 | 3-ketoacyl-CoA thiolase | POT1 | K00632 | Peroxisome. | S |
| KLMA_80402 | 8.4 | 4.4 | 0.010 | probable 26S proteasome complex subunit SEM1 |  | K10881 |  | 45D |
| KLMA_20263 | 8.4 | 8.4 | 0.000 | acyl-coenzyme A oxidase | POX1 | K00232 | Peroxisome (By similarity). | S |
| KLMA_50012 | 8.0 | 8.0 | 0.000 | potassium-activated aldehyde dehydrogenase | ALD4 | K00128 | Mitochondrion matrix. | S |
| KLMA_70429 | 7.8 | 4.5 | 0.001 | mitochondrial 2-methylisocitrate lyase | ICL2 | K01637 | Mitochondrion matrix. | S |
| KLMA_10740 | 7.7 | 2.7 | 0.016 | esterase_lipase super family |  |  |  | S |
| KLMA_70444 | 7.5 | 6.1 | 0.000 | citrate synthase 3 | CIT3 | K01647 |  | S |
| KLMA_40391 | 7.3 | 6.9 | 0.000 | malate dehydrogenase | MDH2 | K00026 | Cytoplasm. | C |
| KLMA_20771 | 6.9 | 5.7 | 0.001 | 12 kDa heat shock protein | HSP12 |  |  | S |
| KLMA_20626 | 6.7 | 2.9 | 0.001 | mitochondrial DnaJ homolog 2 | MDJ2 |  | Mitochondrion inner membrane. | C |
| KLMA_20009 | 6.7 | 5.2 | 0.005 | hypothetical protein | ADY2 | K07034 | Cell membrane; Multi-pass membrane protein. Vacuole membrane; Multi-pass membrane protein. | S |
| KLMA_70304 | 6.5 | 6.3 | 0.000 | transcription elongation factor SPT6 | SPT6 | K11292 | Nucleus (By similarity). | C |
| KLMA_30511 | 5.7 | 4.1 | 0.033 | ATP-dependent RNA helicase DBP3 | DBP3 | K14811 | Nucleus, nucleolus (By similarity). | 30DS |
| KLMA_20345 | 5.6 | 9.4 | 0.006 | protein FUN14 |  |  |  | 30DS |
| KLMA_50277 | 5.3 | 3.8 | 0.001 | conserved hypothetical protein |  |  | Nucleus (Potential). | 45D |
| KLMA_70346 | 5.3 | 3.1 | 0.042 | DNA polymerase alpha subunit B | POL12 | K02321 | Nucleus. | 30DS |
| KLMA_10197 | 5.2 | 8.1 | 0.001 | flocculation protein FLO5 | FLO5 |  | Secreted, cell wall. Membrane; Lipid-anchor, GPI-anchor (Potential). | 45D |
| KLMA_30654 | 5.2 | 6.0 | 0.037 | carnitine O-acetyltransferase | CAT2 | K00624 | Isoform Mitochondrial: Mitochondrion inner membrane; Peripheral membrane protein; Matrix side.Isoform Peroxisomal: Peroxisome. | S |
| KLMA_60323 | 5.2 | 8.3 | 0.000 | uncharacterized transporter YHL008C |  |  | Membrane; Multi-pass membrane protein (Probable). | S |
| KLMA_70426 | 5.2 | 7.7 | 0.000 | peroxisomal hydratase-dehydrogenase-epimerase | FOX2 | K14729 | Peroxisome. | S |
| KLMA_50201 | 5.1 | 4.8 | 0.006 | ribonucleases P/MRP protein subunit POP6 |  | K14524 |  | C |
| KLMA_20587 | 5.1 | 4.8 | 0.025 | ammonia transport outward protein 3 | ATO3 | K07034 | Cell membrane; Multi-pass membrane protein. | S |
| KLMA_40214 | 5.1 | 2.6 | 0.013 | SUR7 family protein FMP45 | FMP45 |  | Cell membrane; Multi-pass membrane protein. | S |
| KLMA_30101 | 5.0 | 5.8 | 0.003 | uncharacterized protein YMR107W | SPG4 |  |  | S |
| KLMA_50023 | 5.0 | 6.4 | 0.001 | hypothetical protein |  |  |  | S |
| KLMA_30656 | 5.0 | 5.7 | 0.001 | ribosomal RNA-processing protein 17 | RRP17 | K14851 | Nucleus, nucleolus. | S |
| KLMA_40173 | 4.9 | 10.7 | 0.000 | peroxiredoxin type-2 | AHP1 | K14171 | Cytoplasm. | 30DS |
| KLMA_20193 | 4.7 | 5.4 | 0.002 | peroxisomal long-chain fatty acid import protein 2 | PXA1 | K15628 | Peroxisome membrane; Multi-pass membrane protein. | 45D |
| KLMA_10715 | 4.7 | 5.6 | 0.048 | sorbose reductase homolog SOU2 | SOU2 |  |  | S |
| KLMA_10051 | 4.7 | 6.7 | 0.001 | glucokinase-1 | GLK1 | K00844 |  | S |
| KLMA_20051 | 4.6 | 4.3 | 0.016 | rRNA-processing protein CGR1 | CGR1 | K14822 | Nucleus, nucleolus (By similarity). | C |
| KLMA_60074 | 4.6 | 5.6 | 0.002 | dicarboxylic amino acid permease | DIP5 | K03293 | Membrane; Multi-pass membrane protein. | S |
| KLMA_70395 | 4.5 | 4.5 | 0.004 | tRNA pseudouridine synthase 4 | PUS4 | K03177 | Nucleus. Mitochondrion. | C |
| KLMA_60262 | 4.5 | 3.0 | 0.050 | UPF0195 protein YHR122W |  |  |  | 30DS |
| KLMA_60046 | 4.5 | 7.0 | 0.040 | uncharacterized protein YGR237C |  |  |  | 45D |
| KLMA_60204 | 4.4 | 7.2 | 0.004 | ribosomal RNA-processing protein 8 | RRP8 | K14850 | Nucleus, nucleolus. Chromosome, telomere (Potential). | 30DS |
| KLMA_20219 | 4.4 | 6.7 | 0.007 | BNI1-related protein 1 | BNR1 |  |  | 45D |
| KLMA_60349 | 4.3 | 4.5 | 0.007 | spindle pole body component SPC105 | SPC105 | K11563 | Cytoplasm, cytoskeleton, spindle pole body. Nucleus membrane; Peripheral membrane protein; Nucleoplasmic side. Chromosome, centromere, kinetochore. | C |
| KLMA_70361 | 4.1 | 4.4 | 0.017 | conserved hypothetical protein | TEC1 | K09450 | Nucleus. | S |
| KLMA_60172 | 4.1 | 7.2 | 0.010 | ribosomal RNA-processing protein 14 | RRP14 |  | Nucleus, nucleolus. | 30DS |
| KLMA_30602 | 4.0 | 7.7 | 0.005 | acetyl-coenzyme A synthetase 1 | ACS1 | K01895 | Microsome (Potential). Endoplasmic reticulum (Potential). | 45D |
| KLMA_80142 | 4.0 | 4.8 | 0.013 | carbonic anhydrase | NCE103 | K01673 | Cytoplasm. Nucleus. | S |
| KLMA_30555 | 3.9 | 11.3 | 0.000 | conserved hypothetical transmembrane protein |  |  |  | 45D |
| KLMA_70145 | 3.9 | 5.4 | 0.016 | conserved hypothetical membrane protein | ywtG |  | Cell membrane; Multi-pass membrane protein (Potential). | S |
| KLMA_20435 | 3.9 | 5.3 | 0.011 | KH domain-containing protein YLL032C |  |  | Cytoplasm. | S |
| KLMA_20658 | 3.8 | 3.3 | 0.037 | uncharacterized membrane protein YMR155W |  |  | Membrane; Multi-pass membrane protein. | S |
| KLMA_70170 | 3.8 | 7.6 | 0.017 | ribonucleoside-diphosphate reductase large chain 1 | RNR1 | K10807 | Cytoplasm. | 30DS |
| KLMA_60246 | 3.8 | 4.1 | 0.026 | cytoplasmic tRNA 2-thiolation protein 2 | NCS2 | K14169 | Cytoplasm (By similarity). | S |
| KLMA_50161 | 3.6 | 7.6 | 0.040 | dihydroxyacetone kinase 1 | DAK1 | K00863 |  | S |
| KLMA_80011 | 3.5 | 6.0 | 0.017 | deoxyribodipyrimidine photo-lyase | PHR1 | K01669 | Nucleus. Mitochondrion. | 45D |
| KLMA_40215 | 3.4 | 7.0 | 0.028 | cell division control protein 13 | CDC13 | K11115 | Chromosome, telomere. | 45D |
| KLMA_80196 | 3.4 | 8.8 | 0.015 | beta-1,3-glucanosyltransferase | GAS1 |  | Cell membrane; Lipid-anchor, GPI-anchor. Secreted, cell wall. | S |
| KLMA_70240 | 3.4 | 6.3 | 0.038 | G2/mitotic-specific cyclin-4 | CLB4 |  |  | S |
| KLMA_30360 | 3.4 | 7.3 | 0.023 | protein TOS1 | TOS1 |  | Secreted (Potential). | S |
| KLMA_40245 | 3.4 | 5.9 | 0.038 | pleiotropic ABC efflux transporter of multiple drugs | PDR5 |  | Cell membrane; Multi-pass membrane protein. | S |
| KLMA_20088 | 3.2 | 6.2 | 0.044 | myosin-1 | MYO1 | K10352 |  | S |
| KLMA_10082 | 3.2 | 5.1 | 0.043 | protein PLM2 | PLM2 |  | Nucleus (Probable). | C |
| KLMA_80261 | 3.0 | 10.4 | 0.012 | uncharacterized protein YMR031C | EIS1 |  | Cytoplasmic granule (By similarity). Cell membrane; Peripheral membrane protein; Cytoplasmic side (By similarity). | S |
| KLMA_20427 | 2.8 | 10.5 | 0.013 | hypothetical protein |  |  |  | 45D |
| KLMA_20813 | 2.8 | 8.9 | 0.047 | tyrosine-protein phosphatase 2 | PTP2 | K01104 | Cytoplasm. Nucleus. | C |
| KLMA_40079 | 2.5 | 10.7 | 0.016 | uncharacterized endoplamic reticulum membrane |  |  |  | 30DS |
| KLMA_40174 | 1.3 | 16.3 | 0.004 | uncharacterized cell wall protein YDR134C |  |  |  | S |

^a^Gene expression was significantly (FDR < 0.05) altered under the following conditions: S, 30X-specific up-regulation; C, commonly up-regulated under 30DS, 45D and 30X conditions; 30DS, up-regulated under 30X and 30DS conditions; 45D, up-regulated under 30X and 45D conditions.

**Table S21** GO terms enriched in significantly down-regulated genes under 30X condition

| GO.ID | Term | Annotated  gene^a^ | Significant^b^ | Expected^c^ | P-value^d^ | Genes |
| --- | --- | --- | --- | --- | --- | --- |
| GO:1901605 | alpha-amino acid metabolic process | 154 | 9 | 2.12 | 0.00020 | ARO10, ARO8, BAT1, FOL2, GAD1, HIS2, HOM2, LYS12, ppr1 |
| GO:0019752 | carboxylic acid metabolic process | 345 | 13 | 4.75 | 0.00056 | ALD5, ARO10, ARO8, BAT1, BIO3, FOL2, GAD1, HIS2, HOM2, LYS12, PDH1, PDX3, ppr1 |
| GO:0009085 | lysine biosynthetic process | 13 | 3 | 0.18 | 0.00064 | ARO8, HOM2, LYS12 |
| GO:0044283 | small molecule biosynthetic process | 263 | 11 | 3.62 | 0.00069 | ALD5, ARO10, ARO8, BAT1, BIO3, FOL2, HIS2, HOM2, LYS12, PDX3, ppr1 |
| GO:0043436 | oxoacid metabolic process | 354 | 13 | 4.88 | 0.00072 | ALD5, ARO10, ARO8, BAT1, BIO3, FOL2, GAD1, HIS2, HOM2, LYS12, PDH1, PDX3, ppr1 |
| GO:0006082 | organic acid metabolic process | 356 | 13 | 4.9 | 0.00076 | ALD5, ARO10, ARO8, BAT1, BIO3, FOL2, GAD1, HIS2, HOM2, LYS12, PDH1, PDX3, ppr1 |
| GO:0016053 | organic acid biosynthetic process | 191 | 9 | 2.63 | 0.00098 | ALD5, ARO8, BAT1, BIO3, FOL2, HIS2, HOM2, LYS12, ppr1 |
| GO:0046394 | carboxylic acid biosynthetic process | 191 | 9 | 2.63 | 0.00098 | ALD5, ARO8, BAT1, BIO3, FOL2, HIS2, HOM2, LYS12, ppr1 |
| GO:0006553 | lysine metabolic process | 15 | 3 | 0.21 | 0.00100 | ARO8, HOM2, LYS12 |
| GO:1901607 | alpha-amino acid biosynthetic process | 119 | 7 | 1.64 | 0.00106 | ARO8, BAT1, FOL2, HIS2, HOM2, LYS12, ppr1 |
| GO:0044282 | small molecule catabolic process | 89 | 6 | 1.23 | 0.00122 | ALD5, ARO10, BAT1, GAD1, PDH1, ppr1 |
| GO:0009066 | aspartate family amino acid metabolic process | 60 | 5 | 0.83 | 0.00125 | ARO10, ARO8, BAT1, HOM2, LYS12 |
| GO:0044281 | small molecule metabolic process | 645 | 18 | 8.88 | 0.00153 | ALD5, APA2, ARO10, ARO8, BAT1, BIO3, CAB1, FOL2, FPS1, GAD1, HIS2, HOM2, KLMA_40628, LYS12, PDH1, PDX3, gcp, ppr1 |
| GO:0006555 | methionine metabolic process | 38 | 4 | 0.52 | 0.00167 | ARO10, ARO8, BAT1, HOM2 |
| GO:0016054 | organic acid catabolic process | 68 | 5 | 0.94 | 0.00219 | ARO10, BAT1, GAD1, PDH1, ppr1 |
| GO:0046395 | carboxylic acid catabolic process | 68 | 5 | 0.94 | 0.00219 | ARO10, BAT1, GAD1, PDH1, ppr1 |
| GO:0044272 | sulfur compound biosynthetic process | 69 | 5 | 0.95 | 0.00234 | ARO10, ARO8, BAT1, BIO3, HOM2 |
| GO:0009081 | branched-chain amino acid metabolic process | 20 | 3 | 0.28 | 0.00238 | ARO10, BAT1, HOM2 |
| GO:0009063 | cellular amino acid catabolic process | 43 | 4 | 0.59 | 0.00265 | ARO10, BAT1, GAD1, ppr1 |
| GO:0006558 | L-phenylalanine metabolic process | 6 | 2 | 0.08 | 0.00269 | ARO10, ARO8 |
| GO:0006570 | tyrosine metabolic process | 6 | 2 | 0.08 | 0.00269 | ARO10, ARO8 |
| GO:0009083 | branched-chain amino acid catabolic process | 6 | 2 | 0.08 | 0.00269 | ARO10, BAT1 |
| GO:1902221 | erythrose 4-phosphate/phosphoenolpyruvate family amino acid metabolic process | 6 | 2 | 0.08 | 0.00269 | ARO10, ARO8 |
| GO:0008652 | cellular amino acid biosynthetic process | 142 | 7 | 1.96 | 0.00294 | ARO8, BAT1, FOL2, HIS2, HOM2, LYS12, ppr1 |
| GO:0000096 | sulfur amino acid metabolic process | 45 | 4 | 0.62 | 0.00313 | ARO10, ARO8, BAT1, HOM2 |
| GO:0009067 | aspartate family amino acid biosynthetic process | 49 | 4 | 0.67 | 0.00428 | ARO8, BAT1, HOM2, LYS12 |
| GO:0006520 | cellular amino acid metabolic process | 241 | 9 | 3.32 | 0.00489 | ARO10, ARO8, BAT1, FOL2, GAD1, HIS2, HOM2, LYS12, ppr1 |
| GO:0006551 | leucine metabolic process | 8 | 2 | 0.11 | 0.00494 | ARO10, BAT1 |
| GO:0034599 | cellular response to oxidative stress | 52 | 4 | 0.72 | 0.00530 | GAD1, KLMA_40301, SCH9, ppr1 |
| GO:1901566 | organonitrogen compound biosynthetic process | 292 | 10 | 4.02 | 0.00551 | ARO8, BAT1, BIO3, CAB1, FOL2, HIS2, HOM2, LYS12, PDX3, ppr1 |
| GO:0009097 | isoleucine biosynthetic process | 9 | 2 | 0.12 | 0.00629 | BAT1, HOM2 |
| GO:0019509 | L-methionine salvage from methylthioadenosine | 9 | 2 | 0.12 | 0.00629 | ARO8, BAT1 |
| GO:0043102 | amino acid salvage | 9 | 2 | 0.12 | 0.00629 | ARO8, BAT1 |
| GO:1901606 | alpha-amino acid catabolic process | 29 | 3 | 0.4 | 0.00698 | ARO10, GAD1, ppr1 |
| GO:0006895 | Golgi to endosome transport | 10 | 2 | 0.14 | 0.00780 | ENT5, SFT2 |
| GO:0071267 | L-methionine salvage | 10 | 2 | 0.14 | 0.00780 | ARO8, BAT1 |
| GO:0006979 | response to oxidative stress | 59 | 4 | 0.81 | 0.00831 | GAD1, KLMA_40301, SCH9, ppr1 |
| GO:0006549 | isoleucine metabolic process | 11 | 2 | 0.15 | 0.00945 | BAT1, HOM2 |
| GO:0019878 | lysine biosynthetic process via aminoadipic acid | 11 | 2 | 0.15 | 0.00945 | ARO8, LYS12 |

^a^The number of GO term annotated genes in the *K. marxianus* genome.

^b^The number of GO term annotated genes, which were significantly (FDR < 0.05) expressed under the condition.

^c^The expected value of Fisher's exact test.

^d^The P-value of Fisher's exact test.

**Table S22** Summary of significantly down-regulated genes under the 30X condition

| Locus_tag | logFC | logCPM | FDR | Product | UniProt gene | KO number | Localization | Specific^a^ |
| --- | --- | --- | --- | --- | --- | --- | --- | --- |
| KLMA_50503 | -18.5 | 8.5 | 0.000 | aspartate-semialdehyde dehydrogenase | HOM2 | K00133 |  | S |
| KLMA_30481 | -17.9 | 7.6 | 0.000 | glyoxalase super family protein |  |  |  | S |
| KLMA_50017 | -16.8 | 7.0 | 0.004 | (2R,3R)-2,3-butanediol dehydrogenase | BDH2 | K00004 | Cytoplasm. Nucleus. | S |
| KLMA_10235 | -16.4 | 6.8 | 0.000 | WW domain-containing protein YFL010C |  |  |  | S |
| KLMA_80090 | -15.8 | 6.2 | 0.005 | uncharacterized protein YGL146C |  |  |  | S |
| KLMA_20536 | -15.3 | 4.8 | 0.000 | yjgF_YER057c_UK114_family |  |  |  | C |
| KLMA_20195 | -14.8 | 5.0 | 0.001 | acetyltransferases | ppr1 |  |  | S |
| KLMA_60491 | -14.6 | 7.1 | 0.001 | 60S acidic ribosomal protein P1-beta | RPP1B | K02942 | Cytoplasm. | S |
| KLMA_70093 | -14.6 | 4.6 | 0.004 | carboxypeptidase S | CPS1 |  | Vacuole membrane; Single-pass membrane protein. | 45D |
| KLMA_50500 | -14.6 | 4.1 | 0.011 | epsin-like protein | ENT5 |  | Cytoplasm. Endosome membrane; Peripheral membrane protein. | C |
| KLMA_30124 | -14.4 | 4.7 | 0.025 | succinate dehydrogenase [ubiquinone] cytochrome b subunit |  | K00236 | Mitochondrion inner membrane; Multi-pass membrane protein (By similarity). | S |
| KLMA_30672 | -14.3 | 6.0 | 0.000 | probable metabolite transport protein C1271.09 |  |  | Membrane; Multi-pass membrane protein (Potential). | 30DS |
| KLMA_30556 | -14.2 | 4.7 | 0.000 | glutamate decarboxylase | GAD1 | K01580 |  | 45D |
| KLMA_70443 | -14.2 | 3.9 | 0.000 | probable 2-methylcitrate dehydratase | PDH1 | K01720 |  | C |
| KLMA_50308 | -14.0 | 6.1 | 0.000 | glycylpeptide N-tetradecanoyltransferase | NMT1 | K00671 | Cytoplasm (By similarity). | S |
| KLMA_80414 | -13.6 | 3.7 | 0.034 | single-stranded nucleic acid-binding protein | SBP1 |  | Cytoplasm. Nucleus, nucleolus. | 45D |
| KLMA_10035 | -13.5 | 3.8 | 0.016 | 5',5'''-P-1,P-4-tetraphosphate phosphorylase 2 | APA2 | K00988 |  | 45D |
| KLMA_10233 | -13.4 | 4.5 | 0.000 | hypothetical protein |  |  |  | 45D |
| KLMA_60021 | -13.2 | 3.7 | 0.040 | carboxypeptidase S | CPS1 | K01293 | Vacuole membrane; Single-pass membrane protein. | S |
| KLMA_70403 | -13.2 | 3.3 | 0.040 | 40S ribosomal protein MRP10 | MRP10 |  | Mitochondrion (By similarity). | 45D |
| KLMA_10726 | -13.2 | 3.3 | 0.000 | mitochondrial protein PET191 | PET191 |  | Mitochondrion. | 45D |
| KLMA_20149 | -12.9 | 3.7 | 0.009 | chromatin structure-remodeling complex subunit RSC7 | NPL6 | K11761 | Nucleus. | S |
| KLMA_60068 | -12.9 | 4.1 | 0.044 | mitochondral 37S ribosomal protein S27 | RSM27 |  | Mitochondrion. | 45D |
| KLMA_40106 | -12.8 | 4.7 | 0.001 | uncharacterized protein YDR210W |  |  |  | 45D |
| KLMA_70126 | -12.7 | 3.1 | 0.019 | uncharacterized protein YJR080C | AIM24 |  | Mitochondrion (By similarity). | 45D |
| KLMA_40044 | -12.5 | 4.7 | 0.009 | ethanolamine-phosphate cytidylyltransferase | ECT1 | K00967 | Cytoplasm. Nucleus. | 45D |
| KLMA_20546 | -12.2 | 4.1 | 0.014 | vacuolar protein sorting-associated protein 62 | VPS62 |  | Membrane; Single-pass membrane protein. | 45D |
| KLMA_R514 | -12.0 | 2.0 | 0.015 | Glu-tRNA |  |  |  | 45D |
| KLMA_40237 | -11.8 | 1.8 | 0.007 | putative glycoprotein endopeptidase KAE1 | gcp | K01409 | Cytoplasm (Potential). | 45D |
| KLMA_40301 | -11.6 | 1.0 | 0.009 | UPF0067 GAF domain-containing protein YKL069W |  | K08968 | Cytoplasm. Nucleus. | C |
| KLMA_20702 | -11.5 | 3.7 | 0.046 | small nuclear ribonucleoprotein Sm D2 | SMD2 | K11096 | Nucleus. | S |
| KLMA_10757 | -11.3 | 2.0 | 0.016 | probable 26S protease subunit YTA6 | YTA6 | K01509 |  | S |
| KLMA_60545 | -10.7 | 5.4 | 0.031 | hypothetical protein |  |  |  | S |
| KLMA_40392 | -10.2 | 5.8 | 0.001 | uncharacterized vacuolar membrane protein YNL305C | BXI1 | K06890 | Endoplasmic reticulum membrane; Multi-pass membrane protein. Vacuole membrane; Multi-pass membrane protein. Mitochondrion membrane; Multi-pass membrane protein. | 30DS |
| KLMA_10488 | -10.2 | 4.0 | 0.012 | GTP cyclohydrolase 1 | FOL2 | K01495 |  | 45D |
| KLMA_40266 | -10.2 | 1.6 | 0.025 | conserved hypothetical protein |  | K13621 |  | S |
| KLMA_10549 | -10.1 | 6.2 | 0.027 | 1,4-alpha-glucan-branching enzyme | GLC3 | K00700 |  | S |
| KLMA_40374 | -8.9 | 5.3 | 0.019 | negative regulator of RAS-cAMP pathway | MKS1 |  | Nucleus. | S |
| KLMA_60402 | -8.9 | 6.6 | 0.034 | pisatin demethylase | PDAT9 | K00493 |  | 45D |
| KLMA_20165 | -8.5 | 5.5 | 0.009 | adenylate kinase 1 | ADK1 | K00939 | Cytoplasm, cytosol (By similarity). Mitochondrion intermembrane space (By similarity). | S |
| KLMA_30338 | -8.3 | 5.1 | 0.002 | protein ICY2 |  |  |  | S |
| KLMA_40404 | -8.1 | 8.8 | 0.000 | aldehyde dehydrogenase 5 | ALD5 | K00128 | Mitochondrion matrix. | S |
| KLMA_30142 | -7.4 | 6.0 | 0.000 | serine/threonine-protein phosphatase PP1-2 | GLC7 | K06269 | Cytoplasm. Nucleus. | C |
| KLMA_30640 | -7.3 | 8.3 | 0.000 | D-amino-acid oxidase | dao1 |  |  | C |
| KLMA_40220 | -6.8 | 11.3 | 0.001 | alcohol dehydrogenase 2 | ADH2 | K13953 | Cytoplasm. | 45D |
| KLMA_10257 | -6.3 | 3.9 | 0.001 | ERAD-associated E3 ubiquitin-protein ligase HRD1 | HRD1 | K10601 | Endoplasmic reticulum membrane; Multi-pass membrane protein (By similarity). | 45D |
| KLMA_20831 | -6.0 | 1.1 | 0.044 | adenosylmethionine-8-amino-7-oxononanoate aminotransferase | BIO3 | K00833 |  | 30DS |
| KLMA_30102 | -5.6 | 3.5 | 0.044 | uncharacterized protein YKL128C | PMU1 |  | Cytoplasm. Nucleus. | 45D |
| KLMA_40260 | -5.6 | 3.1 | 0.006 | hypothetical protein |  |  |  | 30DS |
| KLMA_50151 | -5.3 | 6.1 | 0.025 | aromatic amino acid aminotransferase 1 | ARO8 |  | Cytoplasm. | 30DS |
| KLMA_20158 | -5.1 | 10.0 | 0.000 | alcohol dehydrogenase 4 | ADH4 | K13953 | Mitochondrion matrix. | 45D |
| KLMA_80411 | -4.7 | 4.7 | 0.001 | glycerol uptake/efflux facilitator protein | FPS1 | K03441 | Membrane; Multi-pass membrane protein (Probable). | S |
| KLMA_60412 | -4.5 | 10.7 | 0.000 | hexokinase | RAG5 | K00844 |  | C |
| KLMA_50248 | -4.4 | 8.0 | 0.001 | cAMP-dependent protein kinase type 3 | TPK1 | K04345 | Cytoplasm. Nucleus. | S |
| KLMA_40480 | -4.4 | 6.3 | 0.004 | serine/threonine-protein kinase SCH9 | SCH9 | K08286 |  | S |
| KLMA_50449 | -4.3 | 7.6 | 0.013 | fungal_trans super family conserved domain |  |  |  | C |
| KLMA_40622 | -4.3 | 11.7 | 0.000 | hypothetical protein |  |  |  | C |
| KLMA_10404 | -4.2 | 5.7 | 0.013 | zinc/iron permease | ATX2 | K14715 | Golgi apparatus membrane; Multi-pass membrane protein. | S |
| KLMA_40628 | -4.2 | 4.9 | 0.008 | NADPH-dependent methylglyoxal reductase GRE2 |  |  |  | S |
| KLMA_10436 | -4.1 | 2.6 | 0.030 | histidinol-phosphatase | HIS2 | K04486 |  | S |
| KLMA_10514 | -4.1 | 10.4 | 0.000 | branched-chain-amino-acid aminotransferase | BAT1 | K00826 | Mitochondrion matrix. | C |
| KLMA_20489 | -4.0 | 9.0 | 0.007 | GAL4-like Zn2Cys6 binuclear cluster DNA-binding domain |  |  |  | C |
| KLMA_20152 | -3.8 | 4.7 | 0.016 | probable ADP-ribose 1''-phosphate phosphatase YML087W |  |  |  | S |
| KLMA_30404 | -3.7 | 4.9 | 0.016 | family of serine hydrolases 1 | FSH1 |  | Cytoplasm. Nucleus. | S |
| KLMA_50338 | -3.7 | 4.7 | 0.018 | uncharacterized protein YPL245W |  | K09384 | Cytoplasm. Nucleus. | S |
| KLMA_70134 | -3.7 | 6.8 | 0.019 | protein transport protein SFT2 | SFT2 |  | Golgi apparatus membrane; Multi-pass membrane protein. | 30DS |
| KLMA_20282 | -3.7 | 10.7 | 0.005 | heat shock protein 60 | HSP60 | K04077 | Mitochondrion matrix. | S |
| KLMA_20597 | -3.7 | 5.7 | 0.016 | transaminated amino acid decarboxylase | ARO10 | K12732 | Cytoplasm. | 30DS |
| KLMA_R530 | -3.6 | 5.1 | 0.048 | Gln-tRNA |  |  |  | 30DS |
| KLMA_40627 | -3.6 | 6.0 | 0.019 | siderophore iron transporter ARN1 | GEX1 |  | Cell membrane; Multi-pass membrane protein. Vacuole membrane; Multi-pass membrane protein. | S |
| KLMA_10036 | -3.5 | 5.5 | 0.025 | pantothenate kinase | CAB1 | K09680 | Cytoplasm. Nucleus. | C |
| KLMA_40128 | -3.5 | 10.1 | 0.001 | heat shock protein 26 | HSP26 | K13993 |  | 30DS |
| KLMA_80174 | -3.4 | 8.9 | 0.034 | probable transporter AQR1 | AQR1 |  | Membrane; Multi-pass membrane protein. | C |
| KLMA_50241 | -3.3 | 7.4 | 0.021 | pyridoxamine 5'-phosphate oxidase | PDX3 | K00275 |  | C |
| KLMA_80130 | -3.3 | 6.8 | 0.028 | homoisocitrate dehydrogenase | LYS12 | K05824 | Mitochondrion. | C |
| KLMA_30496 | -3.3 | 6.0 | 0.046 | TPR repeat-containing protein associated with Hsp90 | TAH1 |  | Cytoplasm. Nucleus. | 30DS |
| KLMA_40075 | -3.1 | 4.8 | 0.050 | uncharacterized membrane protein YMR126C | DLT1 |  | Membrane; Multi-pass membrane protein (Potential). | S |
| KLMA_30284 | -3.1 | 8.0 | 0.031 | cytochrome c | CYCK | K08738 | Mitochondrion intermembrane space. | S |
| KLMA_60392 | -2.7 | 9.4 | 0.026 | uncharacterized protein YPL039W |  |  |  | C |

^a^Gene expression was significantly (FDR < 0.05) altered under the following conditions: S, 30X-specific down-regulation; C, commonly down-regulated under 30DS, 45D and 30X conditions; 30DS, down-regulated under 30X and 30DS conditions; 45D, down-regulated under 30X and 45D conditions.
